# A comparison of the effectiveness of cognitive behavioural interventions based on delivery features for elevated symptoms of depression in adolescents: A systematic review

**DOI:** 10.1002/cl2.1376

**Published:** 2024-01-07

**Authors:** Gretchen Bjornstad, Shreya Sonthalia, Benjamin Rouse, Leanne Freeman, Natasha Hessami, Jo Hickman Dunne, Nick Axford

**Affiliations:** ^1^ NIHR Applied Research Collaboration South West Peninsula (PenARC) University of Exeter Medical School Exeter UK; ^2^ Dartington Service Design Lab Buckfastleigh UK; ^3^ MRC/CSO Social and Public Health Sciences Unit University of Glasgow Glasgow UK; ^4^ Center for Clinical Evidence and Guidelines, ECRI Institute Plymouth Meeting Pennsylvania USA; ^5^ The Centre for Youth Impact London UK; ^6^ University of Manchester Manchester UK; ^7^ NIHR Applied Research Collaboration South West Peninsula (PenARC) University of Plymouth Plymouth UK

## Abstract

**Background:**

Depression is a public health problem and common amongst adolescents. Cognitive behavioural therapy (CBT) is widely used to treat adolescent depression but existing research does not provide clear conclusions regarding the relative effectiveness of different delivery modalities.

**Objectives:**

The primary aim is to estimate the relative efficacy of different modes of CBT delivery compared with each other and control conditions for reducing depressive symptoms in adolescents. The secondary aim is to compare the different modes of delivery with regard to intervention completion/attrition (a proxy for intervention acceptability).

**Search Methods:**

The Cochrane Depression, Anxiety and Neurosis Clinical Trials Register was searched in April 2020. MEDLINE, PsycInfo, EMBASE, four other electronic databases, the CENTRAL trial registry, Google Scholar and Google were searched in November 2020, together with reference checking, citation searching and hand‐searching of two databases.

**Selection Criteria:**

Randomised controlled trials (RCTs) of CBT interventions (irrespective of delivery mode) to reduce symptoms of depression in young people aged 10–19 years with clinically relevant symptoms or diagnosis of depression were included.

**Data Collection and Analysis:**

Screening and data extraction were completed by two authors independently, with discrepancies addressed by a third author. CBT interventions were categorised as follows: group CBT, individual CBT, remote CBT, guided self‐help, and unguided self‐help. Effect on depressive symptom score was estimated across validated self‐report measures using Hedges' *g* standardised mean difference. Acceptability was estimated based on loss to follow‐up as an odds ratio. Treatment rankings were developed using the surface under the cumulative ranking curve (SUCRA). Pairwise meta‐analyses were conducted using random effects models where there were two or more head‐to‐head trials. Network analyses were conducted using random effects models.

**Main Results:**

Sixty‐eight studies were included in the review. The mean age of participants ranged from 10 to 19.5 years, and on average 60% of participants were female. The majority of studies were conducted in schools (28) or universities (6); other settings included primary care, clinical settings and the home. The number of CBT sessions ranged from 1 to 16, the frequency of delivery from once every 2 weeks to twice a week and the duration of each session from 20 min to 2 h. The risk of bias was low across all domains for 23 studies, 24 studies had some concerns and the remaining 21 were assessed to be at high risk of bias. Sixty‐two RCTs (representing 6435 participants) were included in the pairwise and network meta‐analyses for post‐intervention depressive symptom score at post‐intervention. All pre‐specified treatment and control categories were represented by at least one RCT. Although most CBT approaches, except remote CBT, demonstrated superiority over no intervention, no approaches performed clearly better than or equivalent to another. The highest and lowest ranking interventions were guided self‐help (SUCRA 83%) and unguided self‐help (SUCRA 51%), respectively (very low certainty in treatment ranking). Nineteen RCTs (3260 participants) were included in the pairwise and network meta‐analyses for 6 to 12 month follow‐up depressive symptom score. Neither guided self‐help nor remote CBT were evaluated in the RCTs for this time point. Effects were generally attenuated for 6‐ to 12‐month outcomes compared to posttest. No interventions demonstrated superiority to no intervention, although unguided self‐help and group CBT both demonstrated superiority compared to TAU. No CBT approach demonstrated clear superiority over another. The highest and lowest ranking approaches were unguided self‐help and individual CBT, respectively. Sixty‐two RCTs (7347 participants) were included in the pairwise and network meta‐analyses for intervention acceptability. All pre‐specified treatment and control categories were represented by at least one RCT. Although point estimates tended to favour no intervention, no active treatments were clearly inferior. No CBT approach demonstrated clear superiority over another. The highest and lowest ranking active interventions were individual CBT and group CBT respectively. Pairwise meta‐analytic findings were similar to those of the network meta‐analysis for all analyses. There may be age‐based subgroup effects on post‐intervention depressive symptoms. Using the no intervention control group as the reference, the magnitudes of effects appear to be larger for the oldest age categories compared to the other subgroups for each given comparison. However, they were generally less precise and formal testing only indicated a significant difference for group CBT. Findings were robust to pre‐specified sensitivity analyses separating out the type of placebo and excluding cluster‐RCTs, as well as an additional analysis excluding studies where we had imputed standard deviations.

**Authors' Conclusions:**

At posttreatment, all active treatments (group CBT, individual CBT, guided self‐help, and unguided self‐help) except for remote CBT were more effective than no treatment. Guided self‐help was the most highly ranked intervention but only evaluated in trials with the oldest adolescents (16–19 years). Moreover, the studies of guided self‐help vary in the type and amount of therapist support provided and longer‐term results are needed to determine whether effects persist. The magnitude of effects was generally attenuated for 6‐ to 12‐month outcomes. Although unguided self‐help was the lowest‐ranked active intervention at post‐intervention, it was the highest ranked at follow‐up. This suggests the need for further research into whether interventions with self‐directed elements enable young people to maintain effects by continuing or revisiting the intervention independently, and whether therapist support would improve long‐term outcomes. There was no clear evidence that any active treatments were more acceptable to participants than any others. The relative effectiveness of intervention delivery modes must be taken into account in the context of the needs and preferences of individual young people, particularly as the differences between effect sizes were relatively small. Further research into the type and amount of therapist support that is most acceptable to young people and most cost‐effective would be particularly useful.

## 1 PLAIN LANGUAGE SUMMARY

### 1.1 Cognitive behavioural therapy is likely to reduce depression in young people, regardless of how it's delivered

Cognitive behavioural therapy (CBT) can be delivered in various ways, all of which are likely to reduce depression in young people. Self‐help with therapist support is the most highly ranked, but the differences between the different types of therapy are small. It is unclear whether therapy delivered remotely (such as online) is effective.

Six to twelve months after treatment, self‐help was the highest ranked for reducing depression. However, fewer types of delivery were tested over the long term, and there is no clear evidence of a difference between them.

No types of delivery are more acceptable to young people than any others.

### 1.2 What is this review about?

Depression is a common mental health problem in young people and is often treated with CBT. CBT is a talking therapy that aims to help people by changing negative patterns in the connections between thoughts, feelings and actions.

CBT can be delivered in different ways: face‐to‐face individually or in a group, via an app or website, using a book, remotely with a therapist online or on the phone or a combination of self‐help and therapist support.

It is not clear which of these types of delivery are the most effective for young people.

### 1.3 What is the aim of this review?

This Campbell systematic review aims to collect information from studies of CBT for depression in young people to find out whether some delivery types are more effective than others for reducing depression, and whether any are more acceptable to young people.

### 1.4 What studies are included?

After searching for studies of CBT for depression in young people, 68 studies were included in the review. All studies are randomised controlled trials.

### 1.5 What are the main findings of this review?

Most types of CBT reduce depression in young people. Self‐help with support from a therapist is the highest ranked for effectiveness, although the differences are small. We only found one study of CBT delivered remotely.

None of the delivery types are more effective than any others 6‐12 months after treatment. Self‐help CBT without therapist support is the highest ranking in the long term. No studies examined self‐help with support from a therapist or CBT delivered remotely over the long term.

No delivery types are likely to be more acceptable to young people than any others.

### 1.6 What do the findings of this review mean?

All types of CBT are likely to reduce depression in young people, although more research is needed for remote CBT.

Self‐help with therapist support is the highest ranked, but it was only tested with young people aged 16–19 years; the results may not apply to younger age groups.

Self‐help without support is the highest ranked in the longer term. Further research is needed to learn whether self‐help enables young people to maintain improvement and whether therapist support would further improve long‐term outcomes.

### 1.7 How up‐to‐date is this review?

The review authors searched for studies up to November 2020.

## 2 SUMMARY OF FINDINGS

Summary of findings Table 1.

**Table 1:** Summary of findings: Post‐test depression score.

Intervention	Comparator	Number of studies (patients) with direct comparison	Relative effect (SMD [95% CI])	Certainty of the evidence	Interpretation^a^
Group	Unguided	2 (247)	−0.20 (−0.77, 0.38)	**Low** Due to concerns with within‐study bias^b^ (some) and heterogeneity^e^ (some)	1.8 fewer points (from 7 fewer to 3.5 more) on the BDI
Individual	Remote	1 (28)	0.54 (−1.28, 2.35)	**Very low** Due to concerns with within‐study bias^b^ (major), indirectness^c^ (some), and imprecision^d^ (major)	4.9 more points (from 11.6 fewer to 21.4 more) on the BDI
Group	Guided	0 (0)	0.38 (−0.56, 1.33)	**Low** Due to concerns with within‐study bias^b^ (some) and heterogeneity^e^ (some)	3.5 more points (from 5.1 fewer to 12.1 more) on the BDI
Group	Individual	0 (0)	0.02 (−0.65, 0.68)	**Very low** Due to concerns with within‐study bias^b^ (some) and heterogeneity^e^ (major)	0.2 more points (from 5.9 fewer to 6.2 more) on the BDI
Group	Remote	0 (0)	0.55 (−1.38, 2.48)	**Very low** Due to concerns with within‐study bias^b^ (some) and heterogeneity^e^ (major)	5 more points (from 12.5 fewer to 22.5 more) on the BDI
Guided	Individual	0 (0)	−0.37 (−1.41, 0.68)	**Very low** Due to concerns with within‐study bias^b^ (some) and heterogeneity^e^ (major)	3.4 fewer points (from 12.8 fewer to 6.2 more) on the BDI
Guided	Remote	0 (0)	0.17 (−1.92, 2.26)	**Very low** Due to concerns with within‐study bias^b^ (some), indirectness^c^ (some), and imprecision^d^ (major)	1.5 more points (from 17.5 fewer to 20.5 more) on the BDI
Guided	Unguided	0 (0)	−0.58 (−1.58, 0.43)	**Low** Due to concerns with imprecision^d^ (major)	5.3 fewer points (from 14.4 fewer to 3.9 more) on the BDI
Individual	Unguided	0 (0)	−0.21 (−0.98, 0.56)	**Very low** Due to concerns with within‐study bias^b^ (some) and imprecision^d^ (major)	1.9 fewer points (from 8.9 fewer to 5.1 more) on the BDI
Remote	Unguided	0 (0)	−0.75 (−2.72, 1.22)	**Very low** Due to concerns with within‐study bias^b^ (some) and imprecision^d^ (major)	6.8 fewer points (from 24.7 fewer to 11.1 more) on the BDI
*Ranking of treatments*			*Intervention (SUCRA value)* *Guided (83%); Remote (77%); Group: (67%); Individual (66%); Unguided (51%)*	**Very low** *Due to concerns with within‐study bias* f *(some), heterogeneity* g *(some), and publication bias* h *(some)*	

**Patient or population**: adolescents aged 10 to 19 years with elevated, clinically relevant symptoms of depression.

**Intervention**: Any cognitive behavioural therapy approach.

**Comparator**: Another cognitive behavioural therapy approach.

**Outcome**: Self‐reported post‐test depression score.

Abbreviations: BDI, Beck's Depression Inventory; CBT, cognitive behavioural therapy; CI, confidence interval; Group, group CBT; Guided, guided self‐help; Individual, individual CBT; PI, prediction interval; Remote, remote CBT; SMD, standardised mean difference; SUCRA, surface under the cumulative ranking curve; Unguided, unguided self‐help.

Network includes 62 randomised controlled trials (6435 participants). Although the table only presents comparisons between active treatments, comparisons with inactive controls did contribute to the analysis.

a Interpretation on BDI scale was estimated from multiplying the standardised relative effects by the median post‐test standard deviation for active interventions evaluated on the BDI scale (9.1 points).

^b^ Studies were at unclear (some concerns) or high (major concerns) risk of bias based on weighted average percentage contribution to effect estimate.

^c^ Studies were moderately (some concerns) or highly (major concerns) indirect based on weighted average percentage contribution to effect estimate. Studies were considered moderately indirect if they included interventions that were evaluated for only younger or older age groups (Araki, 2019; Topooco, 2017, 2019), if they evaluated incarcerated populations (Fischer, 1996; Rohde, 2004; Saranya, 2017), or if they served as the only representative for a direct comparison (Nelson, 2003).

^d^ 95% CI crossed line of no effect and extended past minimum clinically important effect threshold (−3 to 3 points on the BDI) in one (some concerns) or both (major concerns) directions. The minimum clinically important threshold was selected based on previously reported values (Hengartner, 2021).

^e^ 95% PI crossed minimum clinically important effect threshold (−3 to 3 points on the BDI) in one (some concerns) or both (major concerns) directions. Concerns with both heterogenity and imprecision were jointly downgraded, as the two domains are interconnected (e.g., heterogeneity may be the source of imprecision).

^f^ 66% of the information is from studies at unclear risk of bias, 23% from studies at low risk of bias, and 10% from studies at high risk of bias.

^g^ Network heterogeneity parameter (*τ* ^2^ = 0.71) suggests moderate heterogeneity compared to the empirical distribution for non‐pharmacological interventions with mental health outcomes measured on a continuous scale (median *τ* ^2^ = 0.058, 95% range = 0.001 to 2.58) (Rhodes, 2015).

^h^ Publication bias suspected for comparisons with control conditions.

## 3 BACKGROUND

### 3.1 Description of the condition

Depression is a public health problem and common amongst adolescents. In the UK, the rate of mental illness in young people has been rising in recent years and this is expected to continue, particularly due to the COVID‐19 pandemic (The Lancet Child and Adolescent Health, 2021). The Mental Health of Children and Young People (MHCYP) Survey in England 2017 found that 2.1% of children and young people aged 5–19 have a depressive disorder, with the prevalence increasing with age to 4.8% in 17–19‐year olds (NHS Digital, 2018). It is estimated that around one in ten adolescents in the US experiences at least one major depressive episode per year (CBHSQ, 2015). In Europe, the prevalence of depression in adolescents has been reported using baseline data from a randomised controlled trial, which found the prevalence ranging from 7.1% to 19.4% across 11 countries (Balázs, 2012). Research is ongoing to assess how the COVID‐19 pandemic and related restrictions have affected young people's mental health, but a recent analysis indicates that the percentage of young people aged 11–16 years in England with a probable mental disorder increased from 12.6% in 2017 to 17.6% in 2020 (Vizard, 2020).

A diagnosis of major depressive disorder, according to the DSM‐V criteria, is characterised by the presence of five or more symptoms, such as a persistent depressed mood, a loss of interest or pleasure in daily activities, sleep problems, change in appetite or weight, fatigue, feelings of worthlessness or guilt, diminished concentration, and suicidal thoughts, consistently for at least a 2‐week period (American Psychiatric Association, 2013). Clinically significant distress or impairment in social, occupational, or other important areas of functioning must also be present.

The incidence of major depressive disorder in children and adolescents is associated with lifetime psychiatric comorbidity, risk of suicidality, functional impairments, and recurrence (Rohde, 2013). An analysis of global data found that neuropsychiatric disorders were the main cause of disease burden for young people aged 10–24 years, with most of these accounted for by unipolar depressive disorders (Gore, 2011).

In addition, a history of depressive episodes or elevated symptoms of depression are significant risk indicators of later major depressive disorder and can have a negative impact on quality of life (Bertha, 2013). An analysis from the Christchurch Health and Development Study birth cohort in New Zealand found that subthreshold depression in young people aged 17 to 18 years was associated with later depression and suicidal tendencies up to age 25 (Fergusson, 2005). However, a prospective longitudinal cohort study in Australia found that although diagnosis of depression in adolescence predicts diagnosis in young adulthood, the rates of disorder dropped by the late 20s (Patton, 2014). Remission was most likely in cases where adolescent depression was brief in duration. López‐López (2020) analysed data from the Avon Longitudinal Study of Parents and Children (ALSPAC) in the UK and were able to distinguish five different trajectories of depressive symptoms. They found that clinically relevant symptoms at any age between 11 and 24 years, regardless of trajectory, are associated with poor education and employment outcomes in early adulthood, indicating that monitoring of symptoms over the course of adolescence is needed.

The evidence for the incidence, impact, and prognosis of adolescent depression continues to indicate that it is important to identify the most effective interventions to reduce depressive symptoms and limit the duration of diagnoses to improve quality of life in adolescence and into adulthood.

### 3.2 Description of the intervention

This review focusses on interventions that are based on cognitive behavioural therapy (CBT) and delivered through various modalities. CBT is widely used to treat depression amongst children, adolescents and adults and is one of several interventions recommended for treating depression in children and adolescents by the National Institute for Health and Care Excellence in the UK and the American Academy of Child and Adolescent Psychiatry in the US (Birmaher, 2007; NICE, 2017). CBT was also found to have the strongest evidence of clinical effectiveness in a review of digital health interventions for child and adolescent mental health, including for treatment of depression, with non‐CBT digital health interventions having weaker evidence (Hollis, 2017).

CBT is a psychotherapy based on the premise that cognitions, behaviour patterns and emotions are linked, and that cognitive and behavioural techniques can produce changes in these links (Kendall, 1995). According to this model, adolescents with depressive symptomology have negative perceptions about themselves, the world and the future, which affect their behaviours and sustain their feelings of low self‐esteem and hopelessness (Dobson, 2001). For example, depressed individuals will be selective in choosing the evidence for their performance, such that only those instances that support poor performance are remembered, which leads to behaviours that contribute to the development and persistence of depression, such as reduced engagement in activities. This diminished engagement reduces the chance of positive reinforcement. A reduction in positive reinforcement for healthy behaviours may lead to depressive symptomology, and depressed individuals often give up activities that they value. Thus, CBT aims to modify the relationship between thoughts, behaviours and emotions.

### 3.3 How the intervention might work

This review is concerned with examining various delivery modalities of CBT. CBT can be delivered by a therapist, whether in groups or individually, or through self‐help. Therapist support may be delivered face‐to‐face or remotely via video or telephone calls or text messages with a therapist. Further, CBT can be delivered through web‐based programmes that may or may not include communication with a therapist (Rathbone, 2017). Virtual appointments may improve accessibility to therapy and reduce costs. The dose (number, duration and frequency of sessions) of CBT varies and the dose–response relationship is not well understood (Girlanda, 2016).

Self‐help delivery is independent of professional contact and can be delivered via books, computer programmes, or other media. The content and dose are often similar between face‐to‐face and remote therapy, although it may be more difficult to assess the amount of self‐help content received as this relies on self‐report or tracking through technology and uptake and engagement are likely to vary widely (Bergin, 2020; Fleming, 2018). Therapist support can be provided alongside self‐help to guide the patient through the intervention (Cuijpers, 2010). Self‐help CBT can be standardised (i.e., the material is not tailored to individuals and is the same package for all) or personalised (i.e., the material is tailored to individual needs), and it may or may not be interactive.

Digital technologies provide the potential to particularly increase access to treatments for young people, although rates of uptake and the cost‐effectiveness of digital health interventions remains unclear (Hollis, 2017). A recent scoping review of preventive digital mental health interventions for children and young people found that most interventions were delivered in secondary schools and that mental health risk factors were not reported, which may indicate limitations of the reach and applicability of these interventions for young people at risk (Bergin, 2020). In the current COVID‐19 pandemic, the need for remote delivery of mental health interventions has greatly increased, and NHS guidance has been provided to enable practitioners to conduct assessments and therapy remotely (NHS England, 2021).

### 3.4 Why it is important to do this review

Existing research does not provide clear conclusions regarding the relative effectiveness of the different delivery modalities of CBT for depression in children and adolescents. Amongst adults with depression, a previous network meta‐analysis found no statistically significant differences between guided self‐help, individual, group, and telephone delivery of CBT, and those formats were more effective than unguided self‐help (Cuijpers, 2019). However, guided self‐help was found to be less acceptable based on study drop‐out.

A network meta‐analysis of psychotherapies for depression in children and adolescents found that only interpersonal therapy and CBT were significantly more effective than control conditions and were more effective in reducing depressive symptoms than alternative psychotherapies such as play therapies and problem‐solving therapy (Zhou, 2015). However, the review included different delivery modalities of each psychotherapy in the same node and therefore could not draw conclusions about the relative effectiveness of these modalities. Similarly, a recent systematic review of psychological treatments of subthreshold depression in children and adolescents included individual, group, and guided self‐help interventions, finding a small to moderate effect on reducing depressive symptoms, but did not compare the effects of different delivery modes (Cuijpers, 2021).

A systematic review looking at the effectiveness of computerised therapies for anxiety and depression in children and young people identified studies testing three programmes for depression and two programmes aimed at both anxiety and depression; these included interactive games and standardised educational programmes (Pennant, 2015). All of the programmes for depression were rated by the authors as having low therapist input. One programme for depression and anxiety in the general population was rated as having low therapist input, but the remaining two programmes were for populations at risk of anxiety and depression and involved some therapist input. The review found that computerised CBT was more effective than non‐therapeutic controls, but that face‐to‐face therapy was more effective than computerised CBT. A limitation of the review is that it looked at computerised therapies as a whole, rather than categorising them according to whether they were solely self‐help interventions or included therapist support. Similarly, a review of online and social networking interventions for depression in young people found that online interventions with a cognitive behavioural focus were promising in terms of reducing depression (Rice, 2014). This review included studies with varying levels of support, usually from moderators or tutors. It also found a lot of variation between interventions in terms of dropout rates, and it was unclear whether level of support was related to attrition.

Another review that found computerised CBT interventions to be effective in reducing depressive symptoms in children and young people up to age 25 did not differentiate between therapist‐guided and unguided self‐help formats of CBT interventions (Ebert, 2015). Similarly, a review of computerised CBT for anxiety and depression found that included studies varied considerably in terms of therapist support (Richardson, 2010), and a review of internet‐based CBT for children and adolescents found that most interventions involved some therapist support, mainly in the form of written messages or telephone calls (Vigerland, 2016). This is potentially important because there is some evidence based on an analysis of computerised psychotherapies with adults that the effect on depressive symptoms is moderated by the level of therapist support, with larger effects associated with therapist involvement (Andersson, 2009). A survey of young people using Child and Adolescent Mental Health Services in the UK also found that most young people would prefer to talk to a therapist, with only 9% preferring to use a computer programme on their own (Stallard, 2010).

Existing reviews are limited by the lack of primary research comparing the effectiveness of multiple modes of delivering CBT directly, with most studies comparing computerised forms of CBT (either purely self‐help or self‐help with therapist support) with waitlist, no treatment, or treatment as usual (TAU) controls (Calear, 2010; Fleming, 2014). While there are studies that compare the effectiveness of a particular mode of delivery of CBT to no intervention (e.g., van der Zanden, 2012) or another non‐CBT control condition (e.g., Reynolds, 1986), few studies conduct a head‐to‐head evaluation of two different modes of delivering CBT. Existing reviews (Calear, 2010; Ebert, 2015; Fleming, 2014; Pennant, 2015; Rice, 2014) all combine self‐help with therapist support and self‐help without therapist report, making it impossible to determine the relative effectiveness of these two delivery modes and leaving open the question as to whether the addition of therapist support leads to greater effectiveness or patient engagement.

To address this gap, this review utilises network meta‐analysis, a method that includes direct and indirect evidence of the relative effectiveness of different interventions and thus allows comparison of pairs of interventions where there are few or no studies that have tested the two interventions in a head‐to‐head trial. This method also allows for examination of the ranking probabilities of competing modes of delivering CBT based on their relative effectiveness for reducing depression amongst adolescents (Salanti, 2011).

## 4 OBJECTIVES

The current review aims to estimate the relative efficacy of different modes of CBT delivery compared with each other and control conditions for reducing depressive symptoms in adolescents. It provides relative effect estimates and ranking probabilities on the effectiveness of CBT interventions to reduce depressive symptoms in adolescents based on intervention delivery mode.

Primary Question

1. In terms of reducing depressive symptoms in adolescents with elevated symptoms of depression, how do cognitive behavioural interventions differentiated by delivery modes compare to one another and to control groups?
  Secondary Question
2. With regard to intervention completion/attrition (used as a proxy for intervention acceptability), how do cognitive behavioural interventions (for depressive symptoms in adolescents with elevated symptoms of depression) differentiated by delivery modes compare to one another and to control groups?

## 5 METHODS

### 5.1 Criteria for considering studies for this review

#### 5.1.1 Types of studies

The methods for this review were published as a protocol in the Campbell Library (Bjornstad, 2020). This review looked exclusively at randomised controlled trials (RCTs) with pre and post data, including cluster RCTs (i.e., where groups of participants, such as a classroom, rather than individuals, are the unit of random allocation). Cross‐over studies (i.e., where study groups receive two or more interventions in different sequences) were eligible for inclusion if they were RCTs and if they provided data at the end of the first stage. Multi‐arm trials were included. Quasi‐randomised trials (i.e., use of quasi‐random methods of allocation such as alternation, date of birth, case record number), and controlled clinical trials were ineligible to minimise bias which could threaten the validity of the network meta‐analysis. Studies were included irrespective of publication status and language.

#### 5.1.2 Types of participants

The population of interest is adolescents with elevated, clinically relevant symptoms of depression as measured by validated self‐reported measures or diagnostic instruments. Studies including adolescents who meet diagnostic criteria for major depressive disorder were included.

#### 5.1.3 Age

All studies conducted with adolescents aged 10–19 years were included, in line with the WHO definition of adolescence. Studies that included participants who were over age 19 were included in this review only if the mean age of the sample was less than 20 years. Studies conducted with secondary, middle, or high school students were also included (where the age range may differ slightly). Studies conducted with university students were only included where the mean age of the sample was less than 20 years.

#### 5.1.4 Specific characteristics

Studies that include participants of a specific characteristic (e.g., participants of a particular ethnicity or those in families where parents have divorced) were included unless the intervention had been designed specifically for the population or had made adaptations to the content of the intervention and a threat to the transitivity assumption was therefore present.

#### 5.1.5 Diagnosis

This review focused on adolescents with clinically relevant symptoms of depression or who meet diagnostic criteria for major depressive disorder. Symptoms of depression could have been established using diagnostic instruments or scores on self‐report measures.

Table 1 lists self‐reported measures that were included and their cut points. Studies with participants scoring in the clinical range of symptoms of depression based on these self‐reported measures were included. The eligibility criteria were considered first, followed by the baseline scores to identify if the mean score was above the cut point. If other measures were used, they were considered for inclusion based on their validity as measures of depression in adolescents.

**Table 1:** Validated measures of depression.

Measure	Score range	Score cut point
Beck Depression Inventory Second Edition (BDI‐II)	0–63	≥14 (http://academicdepartments.musc.edu/family_medicine/rcmar/beck.htm)
Centre for Epidemiologic Studies Depression Scale Revised (CESD‐R)	0–60	≥16 (http://cesd-r.com/cesdr/)
Children's Depression Inventory (CDI)	0–54 (*t*‐score 34–100)	≥16 (Ivarsson, 2006; Roelofs, 2010)
Children's Depression Rating Scale‐Revised (CDRS‐R)	17–113	≥30 (based on author correspondence)
Children's Depression Scale (CDS)		≥135 (Tisher, 1992)
Hamilton Depression Rating Scale (HDRS)	0–62	≥8 (Hamilton, 1960; Sharp, 2015) *Dependent on the version of the HDRS used in the study
Mood and Feelings Questionnaire (MFQ)	0–66	≥27 (based on author correspondence) ≥5 on the short version (SMFQ); range 0–26 (Thapar, 1998)
Reynolds Adolescent Depression Scale (RCDS)	30–120	*t*‐score of 61 equivalent to a raw score of 76 (Reynolds, 2004)
Patient Health Questionnaire – 9 (PHQ‐9)	0–27	≥5 (Kroenke, 2002)
Patient Health Questionnaire for Adolescents (PHQ‐A, also called the Severity Measure for Depression)	0–27	≥5 (Johnson, 2002)

In cases where the information provided in the study about participant eligibility was unclear, and the author did not provide further clarification when contacted, we included studies if participants were included based on depressive symptoms and if the mean baseline scores were above the cut point for the measure used.

Studies that included adolescents who were deemed to be at risk of developing any form of depressive disorder or who had subthreshold depression were excluded. The exception to this was when the mean depression score at baseline for the intervention and comparison groups fell above the clinically relevant cut point for depressive symptoms mentioned above.

Studies with adolescents with any comorbid disorders (e.g., depression and anxiety, depression and schizophrenia) were only included if the focus of the intervention was the treatment of depression, not comorbid conditions.

Studies were also excluded if their inclusion criteria included adolescents with cognitive impairments (e.g., learning difficulties and autism), or adolescents with chronic or acute physical health conditions, or if the reports stated that adolescents with these types of impairments or conditions were part of the study sample. The purpose of this last criterion was to limit the variation in populations within and across studies in the network, as it is an important effect modifier that has implications for the validity of the network meta‐analysis.

#### 5.1.6 Types of interventions

The review included cognitive behavioural interventions that aimed to reduce symptoms of depression in adolescence, irrespective of delivery mode. For the purposes of this review, an intervention was considered a cognitive behavioural intervention if it included (1) evaluation of cognition to identify dysfunctional cognition, and (2) cognitive restructuring to adopt helpful cognition, and (3) a component focusing on behaviour: behavioural activation, problem‐solving, social skills training or relaxation techniques. We recognised that variation in the third component may confound the estimated difference between treatment delivery modes, and that some interventions may have been partial CBT with more cognitive or more behavioural foci, but we were unable to identify these types of differences between the interventions from the descriptions provided in most studies (Hetrick, 2015).

If the description of the intervention in source documents was not adequate to make an assessment on inclusion based on the above criteria, the author(s) were contacted. If we did not receive further details from the author(s), we included studies that identified the intervention as CBT and excluded studies that did not identify the intervention as CBT.

Studies evaluating interventions that did not have all three CBT components listed above, or which were not identified by the authors as CBT, were excluded.

In line with the above conceptualisation of CBT, interventions such as acceptance and commitment therapy, mindfulness‐based cognitive therapy and dialectical behaviour therapy that are rooted in principles different from those of CBT and focus on helping people to accept thoughts in a non‐judgemental manner were excluded (e.g., Hayes, 1999; Linehan, 2006; Segal, 2012). Studies evaluating rational emotive behaviour therapy (REBT) were included as it is based in similar principles (Ellis, 2003).

Interventions were placed according to their mode of delivery into the following five categories:

1. Therapist‐delivered CBT in face‐to‐face individual sessions (individual CBT): CBT delivered by a therapist to individual clients in face‐to‐face sessions.
2. Therapist‐delivered CBT in group sessions (group CBT): This is similar to the above, but sessions are conducted for a group of clients rather than an individual client.
3. Therapist‐led CBT delivered remotely (remote CBT): This includes CBT that is delivered by a therapist remotely – for example, emails, video calls (e.g., Zoom) and text messaging. The delivery can be to individual clients or groups of clients.
4. Unguided self‐help (unguided self‐help): This involves educating the client in the principles of CBT through reading material and helping them apply it through quizzes and activities. Traditionally, this was referred to as bibliotherapy and included workbooks. CBT can now be provided through various technological platforms (such as smartphone applications or browser‐based programmes), and include audio files and videos in addition to text. When delivered electronically, self‐help may include additional features such as reminders and some basic guidance on how to use the materials.
5. Self‐help with therapist support or guided self‐help (guided self‐help): This involves material to introduce and guide the client through CBT, alongside support from a therapist. For example, clients might gain an understanding of the approach to thoughts via the workbook and could be given homework and have regular feedback calls with a therapist.

Comparisons were classified as (1) no intervention, (2) TAU, and (3) placebo. To be included in the current review, studies must have done one of the following:

1. Compared two cognitive behavioural interventions delivered through different delivery modes. Studies comparing two versions of cognitive behavioural interventions that have the same delivery mode were not included in the network meta‐analyses. If such a study also has another relevant intervention or control group, the two different groups with a common delivery mode were considered as one intervention for the analysis. The way in which the common effect size were determined is explained below.
2. Compared a cognitive behavioural intervention with a no intervention, placebo, services as usual control group. Comparisons in which any pharmacological treatment (e.g., antidepressants), complementary and alternative medicine (e.g., light therapy, acupuncture) or physical interventions (e.g., yoga, exercise) are explicitly provided (i.e., not as services as usual) were not considered because they are beyond the scope of this review. Waitlist controls were classified as no intervention controls. Services‐as‐usual were grouped together to avoid disconnecting the network. Psychological placebos may have included psychoeducation or attention placebos that were not expected to have any impact on the outcome of interest. Psychoeducation is the provision of information about a mental health condition without the provision of therapy. Attention placebo conditions provide similar time and attention from a therapist to participants without the provision of the active therapeutic intervention. Where services‐as‐usual or a placebo were not adequately described in source documents, the author(s) were contacted. If sufficient detail was not obtained, the study in question was excluded.

Studies where CBT was implemented in combination with another intervention were excluded unless the comparison group also received the additional intervention, meaning that the effects of the other intervention would be controlled for.

A sensitivity analysis separating therapist‐led placebos, self‐help placebos, and pill placebos was carried out.

We assumed that any adolescent who met the inclusion criteria was, in principle, equally likely to be randomised to any of the eligible interventions.

**Figure 1:**
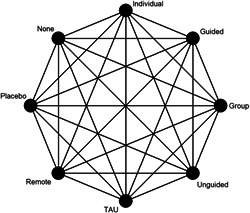
Draft network including all treatment and control nodes.

Figure 1 shows all possible intervention and control comparisons.

#### 5.1.7 Types of outcome measures

##### Primary outcomes

The primary outcome for the review is depressive symptom final score at post‐intervention and at 6–12 month post‐intervention follow‐up assessments. To be included in the analysis, assessment of depressive symptoms must have been self‐report using a validated measure, such as most of those listed in Table 1 (the CDRS‐R and the HDRS were not included as outcome measures as they are not self‐report measures). Eligible outcome measures were limited to self‐report for three reasons: (1) to minimise heterogeneity in outcomes measurement across trials; (2) due to the relative frequency of self‐report measures used in trials of interventions for adolescent depression compared with diagnosis or other measures; and (3) the reliability of self‐report measures of depression symptom severity (Merry, 2012; Mew, 2020; Stockings, 2015). Other self‐report measures of depression symptoms were considered for inclusion in the analysis if used by studies and validated. In cases where there are multiple measurement points within the timeframe of 6–12 months after post‐intervention assessment, we used the measurement point closest to 12 months.

Where insufficient information was provided for endpoint values, we contacted the authors for the required data. If those data could not be provided, the results are described in the narrative summary.

Only continuous measures of depression symptoms assessed using a validated assessment or diagnostic tool were considered. In cases where a study uses multiple appropriate measures for depressive symptoms, we prioritised the measure that is most used across all included studies for consistency across the network. In cases where one particular measure used in a study was not more common in the network than another, we used the measure that the authors of the study consider to be the primary measure.

##### Secondary outcomes

The secondary outcome is acceptability of the intervention. This is defined as the risk of not completing the intervention. This is operationalised as loss to follow‐up in the study by posttest as a dichotomous outcome (Kaltenthaler, 2008).

### 5.2 Search methods for identification of studies

#### 5.2.1 Electronic searches

A search of the following electronic databases was conducted by the Cochrane Common Mental Disorders Group Trials Search Coordinator:

- The Cochrane Depression, Anxiety and Neurosis Group (CCDAN) Clinical Trials Register (CTR) – References Register – Inception to 8 April 2020
- MEDLINE® ALL (Ovid) – Inception to 10 November 2020
- PsycINFO (Ovid) – Inception to November week 1, 2020
- EMBASE (Ovid) – Inception to 20 November 2020
- Cumulative Index to Nursing and Allied Health Literature (CINAHL) (Ebsco) – Inception to 11 November 2020
- International Health Technology Assessment (HTA) Database – Inception to 11 November 2020
- Proquest Dissertations and Theses (Global and UK and Ireland) – Inception to 12 November 2020
- Open Grey – Inception to 19 November 2020

The following trial registry was searched:

- The Cochrane Central Register of Controlled Trials (CENTRAL) via Wiley. Searches were not conducted for ClinicalTrials.gov or the International Clinical Trials Registry Platform (WHO) because CENTRAL now contains records from these two registers.

We were not able to search the International Bibliography of Social Science (IBSS) or PsycExtra due to access limitations, and were not able to search the Educational Technology and E‐Learning (EdiTLib) resource as the interface does not allow search results to be downloaded.

We searched the CCDANCTR‐References Register using the following terms:

Condition:

(depression or depressive) OR depress* or mood*) OR (depress*) adj3 (acute or clinical* or diagnos* or disorder* or elevated or major or unipolar or illness or scale* or scor* or schedule* or adolesc* or child* or ‘young adult*’ or student* or teen* or patient* or participant* or people or inpatient* or in‐patient* or outpatient* or out‐patient*) OR (depress*) and (Beck* or BDI* or DSM* or ‘diagnostic schedule*’ or ‘diagnostic interview*’ or ‘psychiatric assessment*’ or ‘self report*’ or (Statistical Manual adj2 Mental Disorders) or Hamilton or HAM‐D or HAMD or MADRS or (International Classification adj2 Disease*?) or ICD‐10 or ICD‐9 or CESD‐R or CDI or CDRS‐R or CDS or HDRS or MFQ or RCDS or PHQ‐9 or PHQ or PHQ‐A or PHQA or K‐SADS or DISC or DICA‐R or CAPA)

Intervention:

((cogniti* adj behavio*) adj3 (counsel* or intervention or therap* or psychotherap* or training or treatment or technique* or restructur* or defusion)) or counsel* or CBT or CBGT* or bCBT or b‐CBT or iCBT or i‐CBT or ‘rational emoti*’ or (problem* adj2 (focus* or sol*)) or psychoeducat* or ‘role play*’ or schema* or ‘self‐control*’ or ((self* or stress*) adj3 (control or analysis or direct* or esteem or help or instruct* or manage*)) or ((individually or group or conjoint or family) adj2 (counsel* or intervention* or program* or psychotherap* or therap* or train* or treat*)) or ((attribution* or reattribution*) adj3 (therap* or psychotherap*)) or (behavio* adj3 (activation or modification)) or (thought* adj3 suppress*) or rumination or psychodrama or ‘role play*’ or bibliotherap*)

Population:

(child* or boy* or girl* or kids or juvenil* or minors or paediatric* or pediatric* or adolesc* or preadolesc* or pre‐adolesc* or pubert* or pubescen* or prepube* or pre‐pube* or teen* or (young adj (adult* or survivor* or offender* or minorit*)) or youth* or school* or student*)

Search strategies were tailored for each of the remaining databases (Supporting Information: Appendix 1). No restrictions on date, language or publication status were applied to the searches.

#### 5.2.2 Searching other resources

The following sources were hand‐searched:

- Headspace (Australian National Youth Mental Health Foundation) Research Database — the research database for an evidence map of published systematic reviews and controlled studies on depression interventions for young people (Callahan, 2012). The database was filtered for depression, cognitive behavioural therapy and randomised controlled trials and then hand‐searched.
- www.evidencebasedpsychotherapies.org — a database on RCTs of psychotherapies; this was filtered by depression and then hand‐searched.

We searched the reference lists of the following recent reviews on psychotherapies for depression in children and young people: Calear (2010), Ebert (2015), Fleming (2014), Pennant (2015), Rice (2014), and Zhou (2015), as well as reviews retrieved in the search (*n* = 37) (Supporting Information: Appendix 2). Google Scholar was also searched on 30 November 2020, with appropriate search terms from the search strategy to identify newer studies that have cited these reviews. The same search strategy was used to search https://www.learntechlib.org/ on 30 November 2020. The Google search was also conducted on 30 November 2020. 1,290,000 search results were returned by Google and the first 10 pages were copied and pasted to a Word document, from which all links to study reports were extracted and added to Covidence.

Grey literature was also sought through handsearching and review of reference lists as described above and by contacting authors of the reviews listed above.

Ongoing studies identified as likely to meet inclusion criteria based on trial registration or protocol were included in the review as ‘Ongoing or Awaiting classification’. References were managed using Mendeley.

### 5.3 Data collection and analysis

#### 5.3.1 Selection of studies

All references were screened for relevance by title and abstract by two members of the team independently, at least one of whom was an author (G. B., N. A., L. F., or S. S.). Screening and data extraction were managed and stored using Covidence.

**Figure 2:**
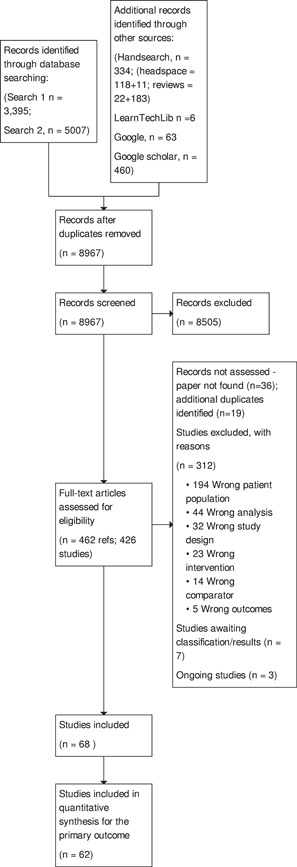
PRISMA flow chart.

The full text of potentially relevant articles was also screened independently by two members of the team (G. B., N. A., L. F., or S. S.) for inclusion. Discrepancies were resolved by consensus and discussion between three authors (G. B., N. A., and S. S.). Eligibility was assessed with reference to a pre‐designed form based on the inclusion criteria. Studies excluded at this stage and reasons for exclusion are presented in Excluded studies. The PRISMA flow chart is presented as Figure 2.

The screening checklist included:

1. Does the study include a relevant intervention?
  a. Is the intervention based on CBT?
  b. Does the intervention target depression?
2. Is the study conducted with adolescents, or students in secondary, middle or high school, or is the mean age between 10 and 19 years?
  a. Specify mean age
3. Is the study conducted with participants who have elevated levels of depression?
  a. Specify screening measure and cut‐off score
4. Is the study an RCT with two different nodes in the network?
  a. Confirm if RCT
  b. Specify all groups and potential nodes

#### 5.3.2 Description of methods used in primary research

The review is limited to randomised controlled trials. The most common type of comparison groups was no intervention. Most studies recruited and randomised individuals based on assessment of depressive symptoms. Studies randomising clusters (such as classrooms) were included.

#### 5.3.3 Criteria for determination of independent findings

Multiple publications of the same study were examined as a single study.

#### 5.3.4 Studies with two or more groups

In a multi‐arm trial where more than one mode of delivering CBT is evaluated, we kept the groups separate and account for correlations due to multi‐arm trials as recommended by (Salanti, 2008).

For multi‐arm trials where not all arms are relevant, we did not include non‐relevant arms in the analysis but will include them in the ‘Table of Characteristics’.

#### 5.3.5 Data extraction and management

Data extraction was done initially in pairs by (first 7 included studies) by N. H., J. H. D. and S. S. independently to ensure consistency, then compared extracted data and S. S. provided some feedback for consistency and clarified doubts on what should be recorded. Subsequent data extraction was done by one researcher and then checked by S. S. to identify and resolve potential discrepancies, with discussion with a third author (G. B.) as required. Study coding for network node was also conducted in pairs by S. S., N. H. and J. H. D., with discrepancies being resolved by a third author (G. B.). Inter‐rater reliability for node identification was analysed using Cohen's kappa statistic based on initial assignments. In cases where one reviewer had coded a node that did not was actually not appropriate to include (e.g., the intervention was used on both adolescents and parents), the discrepancy between the reviewers was not counted as a disagreement. If one reviewer had missed a relevant node from a multi‐arm study that the other reviewer assigned, it was included as a disagreement.

Data were extracted for study design, characteristics of participants and intervention using a data extraction form. Study design includes number of groups, sample size, attrition, recruitment and referral procedures, unit and method of randomisation, data collection methods and timing. Participant characteristics include age, gender, ethnicity, socioeconomic status, baseline depressive symptoms and eligibility criteria. Intervention characteristics include content, format, delivery modality (including details on provider [e.g., therapist qualifications and training, technology platform]), customisation, setting, dosage and implementation fidelity. These details were extracted for all intervention and control groups. Based on the data extraction, study arms were classified into network nodes where relevant.

#### 5.3.6 Assessment of risk of bias in included studies

Two review authors (S. S. and either N. H. or J. H. D.) independently assessed risk of bias using the Cochrane Risk of Bias Tool (Higgins, 2011), with discrepancies or uncertainty resolved by discussion with GB and NA. The following domains were assessed

- Selection bias: Bias due to inadequate randomisation method or allocation concealment method
- Performance bias: Bias due to trial participants and personnel being aware of treatment allocation
- Detection bias: Bias due to outcome assessors being aware of treatment allocation
- Attrition bias: Bias due to amount of missing data in a trial, differential missing data between trial arms, or inadequate methods of handling missing data
- Reporting bias: Bias due to selective outcome reporting
- Other sources of bias:
  ○ Baseline imbalance: Bias due to imbalance in patient characteristics which are strongly related to treatment outcomes
  ○ Contamination bias: Bias due to participants randomised to one group receiving the protocol of a different group of the trial
  ○ Null bias: Bias due to incomplete implementation of treatment group protocol
  ○ Recruitment bias (cluster trials): Bias due to individuals being recruited after clusters are randomised
  ○ Incorrect analysis (cluster trials): Bias due to the analysis not taking the clustering into account

Items were rated for risk of bias as ‘Low risk’, ‘Unclear risk’, or ‘High risk’ following the guidance in the Cochrane Risk of Bias Tool (Higgins, 2011). Performance, detection, and attrition biases were rated for the posttest outcome of depression used in each study.

We rated the overall risk of bias for each outcome within a study using the following key domains: selection bias, detection bias, and attrition bias. The overall risk of bias was rated as ‘Low risk’ if all key domains are rated as ‘Low risk’, ‘Unclear risk’ if at least one key domain is rated as ‘Unclear risk’ and none are rated as ‘High risk’, and ‘High risk’ if at least one key domain is rated as ‘High risk’. We used the overall ratings to inform our assessments of the certainty of the evidence for the quantitative findings.

#### 5.3.7 Measures of treatment effect

##### Relative treatment effects

We evaluated the same effect measures for both the pairwise and network meta‐analyses. For depressive symptom score, a continuous outcome, individual studies used different measures, and therefore we estimated the effect using the Hedges' *g* standardised mean difference (SMD). For acceptability, a dichotomous outcome, we estimated the odds ratio (OR). We report the summary effects and 95% confidence intervals (CIs) for each pair of treatments.

##### Relative treatment ranking

For each outcome, we also estimated the probabilities for all treatments attaining each possible rank. This information was used to develop a hierarchy of rankings using the surface under the cumulative ranking curve (SUCRA) (Salanti, 2011). This approach to ranking accounts for the uncertainty in the treatment effects. A SUCRA value of 0% indicates the treatment is amongst the least effective of the treatments while a value of 100% indicates it is amongst the most effective.

#### 5.3.8 Unit of analysis issues

##### Cluster‐randomised trials

We included cluster‐randomised trials in the analyses along with individually randomised trials. Where necessary, we adjusted standard errors using the methods described in the Cochrane Handbook (Higgins, 2021; White, 2005) using an estimate of the intracluster correlation coefficient (ICC) derived from the trial if provided, from a similar trial, or from a study of a similar population.

We also conducted a sensitivity analysis to investigate the effects of the randomisation unit by excluding cluster‐randomised trials from the analysis.

##### Cross‐over trials

We planned to include cross‐over randomised controlled trials if they provided data at the end of the first stage, but no cross‐over trials met criteria for inclusion.

##### Multi‐arm trial

As mentioned above, when there are two variations of the same mode of delivering of CBT along with a third relevant group, the two CBT groups were combined. For continuous outcomes, this was done by using the formulae provided in table 7.7a in the Cochrane Handbook (Higgins, 2021). For dichotomous outcomes, sample sizes and number of participants with outcome across the groups were summed.

##### Dealing with missing data

In case of missing information, the author(s) of the original study were contacted.

Results using intention‐to‐treat (ITT) analyses were prioritised for extraction, with preference for results based on multiple imputation.

We excluded studies from the quantitative synthesis when mean data was missing for at least one arm. When standard deviation (SD) data were missing, we imputed them based on the median SD for that outcome, measurement scale, and node. We evaluated the impact of this approach by conducting a sensitivity analysis excluding cases where we had imputed SDs.

We recorded the attrition rate and evaluated the risk of bias due to attrition bias.

#### 5.3.9 Assessment of heterogeneity

##### Assessment of clinical and methodological heterogeneity within comparisons

We assessed clinical and methodological heterogeneity by examining the distribution of extracted study, participant, and intervention characteristics (described above) within each direct comparison.

##### Assessment of transitivity across treatment comparisons

We assessed the assumption of transitivity by comparing the distribution of the potential effect modifiers across the different pairwise comparisons. We assessed whether the dose of active treatment was comparable in trials with inactive control groups and trials with active controls and examined differences between treatment nodes in terms of the age of participants and the severity of depression symptoms at baseline.

#### 5.3.10 Assessment of reporting biases

We aimed to minimise the potential impact of reporting biases by conducting a comprehensive search for eligible studies and by being alert to duplication of data. We used comparison‐adjusted funnel plots to explore publication bias and the possibility of small‐study effects across the network (Chaimani, 2015). In order for the results of comparison‐adjusted funnel plots to be meaningful, the treatment comparisons need to be ordered consistently based on the anticipated direction of the small‐study effects. Therefore, we have focused on active treatment versus inactive control comparisons. Comparison‐adjusted forest plots were generated using the netfunnel command in Stata 13® (Chaimani, 2015).

#### 5.3.11 Data synthesis

##### Methods for direct treatment comparisons

A pairwise meta‐analysis was conducted for each pair of interventions (or controls) where there are two or more head‐to‐head trials using random effects models. Pairwise meta‐analyses were performed using the metan command in Stata 13® (Harris, 2008).

##### Methods for indirect and network comparisons

Network meta‐analyses were conducted using random effects models. These analyses followed the multivariate meta‐regression approach accounting for correlations within multi‐arm trials (Lu, 2006; White, 2011, 2012). For the purpose of the analysis, we set the most commonly used intervention (or control) amongst identified trials as the reference. We used the ‘network’ suite of commands in Stata 13® to conduct the network meta‐analyses (White, 2015).

##### Assessment of statistical heterogeneity

###### Assumptions when estimating the heterogeneity

Pairwise meta‐analyses were conducted assuming comparison‐specific heterogeneity (i.e., each direct comparison has a separate heterogeneity estimate). For network meta‐analyses we assumed a common heterogeneity across comparisons.

###### Measures and tests for heterogeneity

Statistical heterogeneity within pairwise comparisons was assessed through *χ* ^2^ tests and *I* ^2^. We considered the following thresholds when interpreting I^2^: 0% to 40% might not be important; 30% to 60% may represent moderate heterogeneity; 50% to 90% may represent substantial heterogeneity; and 75% to 100% represents considerable heterogeneity (Deeks, 2011). We also considered the magnitude and the direction of the effects in our assessment of *I* ^2^. To assess heterogeneity across the entire network, we evaluated the magnitude of *τ* ^2^ and compared it to the empirical distribution (Rhodes, 2015; Turner, 2012).

##### Assessment of statistical incoherence

Incoherence was evaluated using a combination of local and global approaches. Where incoherence was detected, we planned to re‐evaluate the set of studies indicated by the tests which may have been the source of incoherence.

###### Local approaches for evaluating incoherence

Incoherence was evaluated locally using the loop‐specific approach and the node‐splitting approach.

The loop‐specific approach involves examining each closed loop of at least three treatments to determine the agreement between direct and indirect evidence (Higgins, 2012). The difference between the direct and indirect estimate is represented by the incoherence factor and its 95% CI; if the 95% CI is not compatible with 0, it indicates the presence of potential incoherence. We implemented the loop‐specific approach using ifplot in Stata 13® (Chaimani, 2015).

The node‐splitting approach involves examining each pair of treatments individually to compare the direct and indirect estimates (Dias, 2010). Significant differences indicating potential incoherence are detected using a *z*‐test. We implemented the node‐splitting approach using the network sidesplit command in Stata 13® (White, 2015).

###### Global approaches for evaluating incoherence

Incoherence was evaluated in the entire network simultaneously using a design‐by‐treatment interaction model. This model adds terms to represent disagreement between direct and indirect evidence as well as differences by trial design (e.g., two‐arm vs. three‐arm trials) (Higgins, 2012). A Wald test is used to assess potential incoherence. Incoherence models were fitted using the ‘network’ suite in Stata 13® (White, 2015).

#### 5.3.12 Subgroup analysis and investigation of heterogeneity

One potential effect modifier is participant age (Curry, 2006). We conducted exploratory subgroup analyses to investigate the effect of participant age on the primary outcome of post‐test depression score. Subgroups were created by splitting studies according to the mean age of participants as follows: 10–13 years; 14–15 years; ≥16 years. These subgroups were defined a priori based on a study that found differences between age subgroups in response to treatment for depression in adolescents (Curry, 2006). We examined the differences in the results of these subgroups using formal statistical testing. The between‐subgroup comparisons were conducted using fixed‐effects models and defining a statistically significant subgroup effect as *p* < 0.10.

#### 5.3.13 Sensitivity analysis

The primary analysis assumed that all placebo control conditions were similar enough to group together in the same node in the network. To evaluate the appropriateness of this assumption, we carried out a sensitivity analysis separating therapist‐led placebos, self‐help placebos, and pill placebos.

We also conducted a sensitivity analysis to exclude studies where we coded the intervention as interactive to examine whether interactive interventions confounded the estimated difference between delivery modes.

Finally, we conducted a sensitivity analysis to investigate the effects of the randomisation unit, as both cluster‐randomised and individually randomised were included in the main analysis.

All sensitivity analyses were conducted for the primary outcome at the post‐intervention time point.

We planned to conduct sensitivity analyses to test whether differences in intervention components confound the estimated differences between delivery modes, but were unable to do this as the descriptions of the interventions in most studies were not sufficient to identify these types of differences. We also planned to conduct a sensitivity analyses excluding studies where symptoms of depression were established using an unvalidated measure or unclear method, but all included studies measured depression at baseline using at least one validated measure.

#### 5.3.14 Summary of findings and assessment of the certainty of the evidence

The main Summary of findings Table 1 is based on GRADE recommendations. The table includes the comparison, number of studies and patients contributing to direct evidence, relative effect size, and quality of evidence for active treatment comparisons. Further details are summarised in narratively and in additional tables (capturing the intervention details and study details). The adaptation of GRADE to network meta‐analysis was implemented using the CINeMA web application (http://cinema.ispm.ch/).

We also assessed the certainty of treatment rankings based on the Salanti framework (Salanti, 2014). The geometry of the network is described according to the PRISMA guidelines (Hutton, 2015). We also present the findings including effect size (against a control group), confidence intervals, and SUCRA rankings. The relative effectiveness of all interventions against each other has been summarised in a matrix. To aid in clinical interpretation, the Summary of findings Table 1 includes a column where the standardised effects are converted to BDI scores using the median post‐test SD for interventions evaluated on the BDI scale.

## 6 RESULTS

### 6.1 Description of studies

#### 6.1.1 Results of the search

Figure 2 shows the PRISMA diagram, created using the R package and ShinyApp for producing PRISMA 2020 compliant flow diagrams (Haddaway, 2021), and transferred manually into RevMan Web.

The number of references identified by the searches was 9265. 3395 came from the first search of databases and 5007 came from the second search. In addition, 460 studies were identified via Google Scholar, 63 via Google and 6 via the Learning and Technology Library (https://www.learntechlib.org/). 334 studies were added through the handsearch, of which 129 came from Headspace (https://headspace.org.au/) and 205 were identified from relevant reviews.

Of these, 8967 remained after de‐duplication. We excluded 8505 after screening titles and abstracts, retrieving 462 full‐text papers which corresponded to 426 individual studies. 55 reports were not retrieved (36 papers were not found, 19 were duplicates). We excluded 312 studies with reasons outlined in the PRISMA diagram (Figure 2), and indicated 10 as ongoing studies or as awaiting classification due to lack of sufficient information to complete screening. This resulted in 85 references being included in the review for a total of 68 studies. We contacted 34 authors for further information, of whom 14 responded and 9 provided the information necessary to proceed with analysis. We could not find contact details for 5 authors. Percentage agreement for selection of studies at the abstract stage was 93.2% and at the full text stage was 86.7%.

All studies were included in at least one of the quantitative syntheses. Of the included studies, 62 were included in the data synthesis for the primary outcome of post‐test depression score and 6 were not included due to having insufficient data for analysis. One study was excluded from data analysis as there was a discrepancy between the inclusion criteria reported in the text and the baseline scores reported in the table, and this could not be verified with the authors. The study was included in the review based on the reported inclusion criteria.

#### 6.1.2 Included studies

Table 2 provides an overview of the studies included in the review.

**Table 2:** Characteristics of included studies.

References	Nodes	Number randomly assigned to each group	Inclusion criteria	Mean age (Age range)	Additional characteristics	Country (language)	Setting	Dosage	Outcome measure	Mean baseline scores (SD)	Follow‐up Points (in months)	Type of Publication	Funding Source
Ackerson (1998)	None; Unguided (not interactive)	10; 12	CDI > 10; HRSD > 10	15.9 (14–18)		USA (English)	Home	N/A; Completed during own time over 4 weeks	CDI	16.8 (4.5); 19.7 (7.1)		Published	Not reported
Alavi (2013)	TAU; Individual	15; 15	MDD/suicide attempt in last 90 days	16 (12–18)		Iran (English)	Clinical	NR; 12‐weekly sessions	BDI	27.78 (4.11); 30.58 (5.35)		Published	Shiraz University of Medical Sciences (Iran)
Araki (2019)	Placebo (self‐help); Guided (not interactive)	65; 69	None	19.5 (NR)	College students	Japan (English)	College/University	1 90‐min group session and 2 months no follow up; 1 90‐min group session and 2 months of follow‐up homework exercises	CES‐D	23 (12.4); 19.7 (9.6)		Published	Not reported
Bella‐Awusah (2016)^a^	None; Group	20; 20	BDI > 18	15.6 (14–17)		Nigeria (English)	School	N/A; 5‐weekly 45‐ to 60‐minute sessions	BDI, SMFQ	24.2, 11.8 (6.1, 2.7); 25.3, 13.4 (8.8, 4.5)		Published	John D and Catherine T MacArthur Foundation (USA)
Brent (1997)	Placebo (supported); Individual	35; 37	BDI > 13	15.6 (13–18)	MDD per DSM‐III	USA (English)	Primary Care	NR; 12 to 16 approximately 1‐h‐weekly sessions followed by 2 to 4 monthly booster session phase: 2‐4 sessions	BDI	25.7 (7.8); 24.3 (8.1)		Published	National Institute of Mental Health (USA)
Briere (2019)	Placebo (self‐help); Group	37; 37	CES‐D > 20	15.5 (14–18)		Canada (English)	School	NR; 6‐weekly 1‐h sessions	CES‐D	1.66 (0.39); 1.55 (0.37)		Published	Fonds de Recherche Québécois sur la Société et la Culture (Canada)
Charkhandeh (2016)	None; Individual	60; 65	CDI ≥ 20; DSM‐IV‐TR (MDD)	NR (12–17)		Iran (English)	Primary Care	N/A; 24 twice‐weekly 90‐min sessions	CDI	30.35 (5.45); 29.46 (5.47)		Published	Not reported
Clarke (1995)	TAU; Group	74; 76	CES‐D > 20	15.3 (NR)		USA (English)	NR	NR; 15 thrice‐weekly 45‐min sesisons	CES‐D	21.88 (9.2); 24.29 (9.6)	12	Published	National Institute of Mental Health (USA)
Clarke (1999)	None; Group	27; 37	DSM‐III‐R (MDD or dysthemia)	16.2 (14–18)		USA (English)	Clinical	N/A; 16 twice‐weekly 2‐h sessions followed by 1 to 2 booster sessions	BDI	24.2 (10.8); 26.5 (9.4)		Published	National Institute of Mental Health (USA)
Clarke (2001)	TAU; Group	49; 45	DSM‐III‐R (MDD or dysthemia)	14.5 (13–18)		USA (English)	Clinical	NR; 13 1‐h sessions	CES‐D	23.8 (10.3); 25.2 (8.7)	12	Published	National Institute of Mental Health (USA)
Clarke (2002)	TAU; Group	47; 41	DMS‐III‐R	15.3 (13–18)	Major depressive disorder (MDD) or dysthemia per DSM‐III‐R	USA (English)	Primary Care	NR; 16 twice‐weekly 2‐h sessions	CES‐D	34.2 (9.8); 33.5 (8.3)	12	Published	National Institute of Mental Health (USA)
Clarke (2016)	TAU; Individual	106; 106	DSM‐IV‐TR/K‐SADS (MD)	14.6 (12–18)		USA (English)	Primary Care	NR; 2, 4‐session modules (unclear on time frame of delivery), followed by monthly ph1 calls (up to 6 as required)	CES‐D	27.96 (7.74); 28.34 (7.58)	9	Published	National Institute of Mental Health (USA)
Congleton (1996)	None; Group	5; 10	Systematic screening for behaviour disorders – teachers nominated students with internalising behaviour; CDI used but not for screening	13.5 (12–15)		USA (English)	School	N/A; 8 roughly twice‐weekly 1‐h sessions over 5 weeks	CDI (pretest), YSR – Depression (posttest)	48.2 (9.07); 49.4 (10.61)		Published	Not reported – PhD
Cui (2016)	Placebo (supported); None; Group	60; 60; 60	SDS >30, <56	19.42 (18–21)		China (English)	College/University	8 weekly, 2‐h sessions; N/A; 8 weekly, 2‐h sessions	SDS	43.82 (19.23); 43.64 (18.62); 44.14 (19.07)		Published	National Natural Science Project (China)
Curtis (1993)	None; Group	11; 12	BDI ≥ 16; DSM‐III‐R/CAS (MDD, dysthymia or adjustment disorder with depressed mood)	15.8 (NR)	Major depression, dysthymia, or adjustment disorder with depressed mood diagnosis per DSM‐III‐R	USA (English)	School	N/A; 12 roughly twice‐weekly, 2‐h after school sessions over an 8 week period	BDI, RADS	24.6 (6.4), 61 (3.8); 26.6 (10.2), 56.1 (13.4)		Unpublished	Not reported – PhD
De Cuyper (2004)	None; Group	11; 9	DSM‐III‐R (at least one criterion of MDD); CDI (cut‐off score not reported)	10 (9–11)		Belgium (English)	NR	N/A; 16 1‐h‐weekly sessions followed by 2 booster sessions (1 and 4 months later)	CDI	15.27 (4.54); 12.67 (6)		Published	Not reported
Dobson (2010)	Placebo (supported); Group	21; 25	CES‐D ≥ 24	15.2 (13– 18)		Canada (English)	School	15 sessions (length/frequency and duration unclear); 15 45‐min sessions (frequency/duration unclear)	CDI, CES‐D, MASQ‐D	36.57 (5.34), 34.1 (11.08), 57.5 (15.09); 36.08 (5.63), 30.44 (7.02), 54.24 (14.05)		Published	Alberta Heritage Foundation for Medical Research (Canada)
Ede (2019)	None; Group	80; 82	CES‐DC (moderate or severe clinical depression)	18 (16–21)		Nigeria (English)	College/University	N/A; 12‐weekly 1‐h sessions	CES‐D	50.44 (7.68); 49.1 (6.25)		Published	Self‐funded
Ettelson (2003)	None; Group	12; 13	DSM‐IV	15.5 (14–18)	Major Depressive Disorder (MDD), Dysthymic Disorder (DD), or sub‐clinical MDD per DSM0‐IV	USA (English)	School	N/A; 16 twice‐weekly 50 minute sessions over 8 weeks	CDI	63.25 (11.25); 69.31 (15.47)		Unpublished	Not reported – PhD
Fischer (1996)	Placebo (supported); Group	8; 8	BDI > 14	NR (12–17)	Detained adolescents at Detention Centre	USA (English)	Penal	5 90‐min session 2× per week over 3 weeks; 5 90‐min session 2× per week over 3 weeks	BDI	20.38 (8.2); 24.25 (10.34)		Unpublished	Not reported – PhD
Fleming (2012)	None; Unguided (interactive)	11; 19	CDRS‐R ≥ 30	14.9 (13–16)		New Zealand (English)	School	N/A; 7 approximately 30‐min modules completed at a rate of 1–2 modules/week over 5 weeks	CDRS‐D, RADS	39.5 (NR), 70.5 (NR); 39.6 (NR), 70.3 (NR)		Published	New Zealand Ministry of Health; New Zealand Tertiary Education Commission
Gaete (2016)	None; Group	113; 229	BDI >10 (boys), >15 girls	15.9 (14–19)		Chile (English)	School	N/A; 8‐weekly 45‐min sessions	RADS	21.9 (8.5); 22.53 (9.53)		Published	Wellcome Trust (UK)
Garber (2009)	TAU; Group	157; 159	CES‐D ≥ 20 or previous episode of MDD (DSM‐IV)	14.8 (13–17)		USA (English)	Clinical	NR; 8‐weekly 90 min sessions followed by 6 monthly 90‐min continuation sessions	BDI	15.8 (10); 15.5 (9.4)	12	Published	National Institute of Mental Health (USA)
Hamamci (2006)	None; Group	11; 10	BDI ≥ 19; DAS (above average); ATQ (above average)	19.52 (NR)		Turkey (English)	College/University	N/A; 12‐weekly 90‐min sessions	CES‐D	29.87 (8.5); 27.1 (7.32)		Published	Not reported
Idsoe (2019)^a^	TAU; Group	95; 133	BDI > 10	16.8 (15–18)		Norway (English)	Community	NR; 8‐weekly 2‐h sessions, or twice‐weekly sessions over a shorted period, followed by 2 90‐min	BDI	30.28 (10.67); 32.77 (8.8)	12	Published	The Research Council of Norway; Gidske og Peter Jacob Sørensens fond; Norwegian Directorate of Health
Ip (2016)	Placebo (self‐help); Unguided (interactive)	127; 130	CESD‐R >12, <40	14.6 (13–17)		Hong Kong (English)	School	NR; 10 modules, completed over 8 months at own pace	CES‐D	20.43 (9.43); 20.66 (9.32)		Published	Lotteries Fund for Pilot Cyber Youth Outreaching Project (Hong Kong)
Kaesornsamut (2012)	None; Group	30; 30	CES‐D >16, <29	16.9 (16–18)		Thailand (English)	School	N/A; 12‐weekly 1‐h sessions	CES‐D	20.6 (3.44); 19.23 (2.78)		Published	Thailand Nursing and Midwifery Council
Kahn (1988)	None; Group	17; 17	RADS‐I > 72; CDI	13.5 (10–14)		USA (English)	Clinical	N/A; 12 50‐min sessions over 6 to 8 weeks	CES‐D, RADS	28.46 (13.23), 86.91 (11.71); 26.53 (11.05), 85.41 (11.48)		Unpublished	University of Utah (USA)
Kennard (2006)	Placebo (pill); Individual	112; 111	CDRS‐R > 45	14.6 (12–17)	Major depressive disorder (MDD) per DSM‐IV	USA (English)	Primary Care	Medication; 14 twice‐weekly 1‐h sessions	CDI	81.26 (9.22); 78.69 (10.59)		Published	National Institute of Mental Health (USA)
Kerfoot (2004)^a^	TAU; Individual	23; 29	MFQ > 23	13.7 (NR)		UK (English)	Primary Care	NR; 8‐weekly sessions	RADS	34.2 (8.3); 33.7 (8)		Published	National Health Service Executive (North West) (UK)
Kobak (2015)^a^	TAU; Individual	37; 39	QIDS‐A‐SR > 11	15.4 (12–17)	Mood disorder per DSM‐V	USA (English)	School	NR; 12 weeks of treatment (frequency and length of sessions not stated) plus individualised text messages	RADS	NR (NR); NR (NR)		Published	National Institute of Mental Health (USA)
Lamb (1998)	None; Group	19; 27	RADS > 66	15.8 (14–19)		USA (English)	Community	N/A; 8 week long programme (frequency/length unclear)	MFQ	NR (NR); NR (NR)		Published	National Center for Nursing Research (USA)
Lewinsohn (1990)	None; Group	19; 21	DSM‐III (MDD)/RDC (current episode of depressive disorder)	16 (14–18)	Major depression per DSM‐III, current episode of minor depression per RDC	USA (English)	School	N/A; 14 twice‐weekly 2‐h sessions	BDI, CES‐D	23.84 (11.43), 14.89 (4.3); 21.67 (11.34), 13.29 (5.21)	12	Published	National Institute of Mental Health (USA)
Listug‐Lunde (2004)	TAU; Group	8; 9	CDI > 15	12.4 (NR)		USA (English)	Home	NR; 13 twice‐weekly 35‐ to 40‐min sessions for 7 weeks followed by 2 booster sessions within 1 month after treatment	CDI, CES‐D	20.38 (4.1), 25.26 (11.72); 21 (4.95), 22.25 (8.51)		Unpublished	Not reported – PhD
Makarushka (2012)	Placebo (self‐help); Unguided (interactive)	85; 76	CES‐D > 13	12.7 (NR)		USA (English)	School	N/A; Completed during own time over 6 weeks at about 1 module per week	CES‐D	27.19 (9.12); 26.91 (8.74)		Unpublished	National Institute of Mental Health (USA)
Marcotte (1993)	None; Group	13; 12	BDI > 15; HDRS > 10	15 (14– 17)		Canada (French)	School	N/A; 12 twice‐weekly 45‐ to 60‐min sessions	BDI	21.385 (6.33); 24 (8.2)		Published	Not reported
McLaughlin (2011)	TAU; Group	11; 11	BYI‐II/CES‐D (exhibited signs of depression)	11.82 (10–15)		USA (English)	Primary Care	NR; 10‐weekly 50‐min sessions	BDI, CES‐D	49.73 (8.64), 18.64 (8.08); 57.18 (9.8), 20.18 (9.38)		Unpublished	Not reported – PhD
Merry (2012)	TAU; Unguided (interactive)	93; 94	PHQ‐9/self‐assessed (mild to moderate depressive disorder)	15.6 (12–19)		New Zealand (English)	Primary Care	NR; 7 modules completed during own time over 4‐ to 7‐weeks	RADS	75.52 (14.42); 74.83 (13.35)		Published	New Zealand Ministry of Health
Moldenhauer (2004)	Placebo (supported); Individual	11; 15	CDI >15, <26	14.6 (12–17)		USA (English)	Primary Care	NR; 6 1‐h‐weekly sessions plus 6‐weekly 45‐min individual parent sessions	CDI	15.45 (7.03); 19.8 (9.47)		Unpublished	National Research Science Award; Sigma Theta Tau International; KM Donahue (USA)
Nelson (2003)	Remote; Individual	19; 19	DSM‐IV (MDD)	10.3 (8–14)	Depression diagnosis per DSM‐IV	USA (English)	Primary Care	6 1‐h‐weekly sessions plus 6‐weekly 45‐min individual parent sessions; 1 90‐min session followed by 7‐weekly 60‐min sessions between the parent and the child	CDI	13.57 (9.85); 14.36 (8.75)		Published	Not reported – PhD
Parks (2013)	None; Unguided (not interactive)	18; 20	None	NR (NR); college freshmen	College freshmen	USA (English)	College/University	N/A; Over a period of 8 weeks	CES‐D	18.8 (n/a); 19.5 (n/a)		Published	Not reported
Phillips (2005)	None; Group	31; 33	BDI > 10	17.7 (15–20)		USA (English)	School	N/A; 6 1‐h‐weekly sessions	BDI	18.65 (); 16.48 ()		Unpublished	Not reported – PhD
Rajabi (2018)	Placebo (supported); Group	10; 10	CDI > 22	NR (13–15)		Iran (Persian)	Education Counselling Centre	12 twice‐weekly 90‐min sessions; N/A	CDI	32.8 (NR); 29.2 (NR)		Published	Not reported
Reynolds (1986)	None; Group	10; 9	BDI > 12; RADS‐I > 72; BID > 20	15.65 (NR)		USA (English)	School	10‐weekly 50‐min sessions; 16 twice‐weekly 2‐h sessions	BDI, RADS	16.9 (5.48), 80.7 (3.58); 21.11 (7.75), 85.67 (8.4)		Published	Wisconsin Alumni Research Foundation (USA)
Rohde (2006)	Placebo (supported); Group	48; 45	KSADS (MDD)	15.2 (13–17)	Juvenille Justice Referral; had conduct disorder	USA (English)	Penal	16 twice‐weekly 2‐h sessions followed by 2 optional sessions for parents; 6‐weekly 1‐h sessions with individual catch‐ups when sessions were missed	BDI	15.4 (10.6); 16.6 (12.8)	12	Published	National Institute of Mental Health (USA)
Rohde (2014a)	TAU; Unguided (not interactive); Group	33; 22; 27	CES‐D (endorsed two or more items)	19 (17–22)		USA (English)	School	6‐weekly 1‐h sessions with individual catch‐ups when sessions were missed; NR; Completed during own time over 6 weeks	K‐SADS	1.43 (0.35); 1.51 (0.41); 1.52 (0.39)	12	Published	National Institute of Health (USA)
Rohde (2014b)	TAU; Unguided (not interactive); Group	124; 128; 126	CES‐D (endorsed two or more items)	15.5 (13–19)		USA (English)	College/University	NR; Completed during own time over 6 weeks; 6‐weekly 1‐h sessions with 10‐ to 15‐minute individual catch‐ups when sessions were missed	K‐SADS	1.38 (0.36); 1.45 (0.41); 1.37 (0.35)	12	Published	National Institute of Health (USA)
Rossello (1999)	None; Individual	23; 25	DSM‐IV(MDD or dysthymia)	14.7 (13–17)	Major depressive disorder (MDD) or dysthemia per DSM‐III‐R	Puerto Rico (English)	Clinical	N/A; 12 1‐h‐weekly sessions	CDI	20.13 (5.99); 20.12 (6.95)		Published	National Institute of Mental Health; University of Puerto Rico (USA)
Sanchez‐Hernandez (2016)	None; Group	12; 13	CES‐D (child) > 15	11.1 (10–12)		Spain (English)	NR	N/A; 12 twice‐weekly 2‐h sessions	CES‐D	19.52 (8.03); 22.38 (6.54)		Published	University of Murcia (Spain)
Saranya (2017)	TAU; Guided (interactive)	42; 42	PHQ‐9 >9, <19	17.7 (16–19)	Incarcerated	Thailand (English)	Penal Setting/Vocational Training	NR; 6‐weekly 45‐ to 60‐min sessions	PHQ‐9	11.52 (2.73); 11.88 (2.86)		Published	Department of the Ministry of Justice (Thailand)
Saw (2019)	None; Group	10; 10	RADS‐II > 76	16 (16)		Malaysia (English)	School	N/A; 8‐weekly 90‐min sessions	RADS	79.1 (3.67); 80.2 (4.83)		Published	Deanship of Scientific Research, King Fahn University; Global Asia 21st Century (Malaysia)
Saw (2020)	None; Group	43; 42	RADS‐II > 76	16 (16)		Malaysia (English)	School	N/A; 8‐weekly 1‐h sessions	RADS	84.37 (4.68); 83.88 (4.6)		Published	Universiti Teknologi MARA; Ministry of Higher Education (Malaysia)
Sheffield (2006)^a^	None; Group	149; 134	CDI + CES‐D score top 20%	14.34 (14–15)		Australia (English)	School	N/A; 8‐weekly 90‐min sessions	CDI, CES‐D	23.3 (7.82), 30.24 (9.13); 21.3 (7), 27.55 (9.07)	12	Published	Not reported
Singhal (2018)^a^	Placebo (supported); Group	55; 65	CDI >14, <24	NR (13–18)		India (English)	School	45‐min sessions (number/duration unclear); 8‐weekly sessions (time not specified, but control was 45 min)	CDI, CES‐D	21.8 (3.5), 29.5 (5.3); 22 (4.3), 29.4 (6.4)		Published	National Institute of Mental Health and Neuro Sciences (India)
Srivastava (2015)	TAU; Unguided (interactive)	10; 11	ICD‐10 (mild/moderate unipolar depression)	16.5 (15–19)		India (English)	Primary Care	NR; 12‐weekly sessions	BDI	24.3 (3.8); 26.4 (3.4)		Published	Indian Council of Medical Research
Stallard (2012)^a^	Placebo (supported); None; Group	374; 298; 392	SMFQ > 15	14.1 (12–16)		UK (English)	School	9 weekly or fortnightly 50‐ to 60‐min sessions; N/A; 9 modules plus 2 booster sessions weekly or fortnightly 50‐ to 60‐min sessions	SMFQ	10.6 (4.67); 10.56 (4.93); 10.64 (4.91)	6	Published	National Institute for Health Research (UK)
Stasiak (2014)	Placebo (self‐help); Unguided (interactive)	17; 17	CDRS‐R ≥ 30/RADS‐II ≥ 76	15.2 (13–18)		New Zealand (English)	School	7 25‐ to 30‐min modules over 4 to 10 weeks completed in own time; 7 25‐ to 30‐min modules over 4 to 10 weeks completed in own time	RADS	66.12 (13.38); 77.47 (12.64)		Published	Not reported
Stice (2007)	Placebo (supported); Placebo (self‐help); Unguided (not interactive); Group; None	19; 61; 28; 50; 67	CESD > 20; BDI < 30	18.4 (15–22)		USA (English)	School	4‐weekly sessions; completed during own time over 4 weeks; completed during own time over 4 weeks; 4‐weekly 1‐h sessions; n/a	BDI	19.05(6.41); 19.95 (5.99); 20.28 (5.78); 20.58 (6.55); 19.38 (5.98)	6	Published	Hogg Foundation; National Institute of Health (USA)
Stice (2008)	Placebo (supported); TAU; Unguided(not interactive); Group	88; 84; 80; 89	CES‐D > 20	15.6 (14–19)		New Zealand (English)	Primary Care	6‐weekly 1‐h sessions; NR; Completed during own time over 6 weeks; 6‐weekly 1‐h sessions	BDI	20.27 (9.83); 19.6 (9.23); 18.21 (7.53); 20.12 (10.38)	12	Published	National Institute of Health (USA)
Stikkelbroek (2020)	TAU; Individual	36; 34	KSADS (MDD)	16.6 (12–21)		The Netherlands (English)	Clinical	NR; 15 45‐min‐weekly sessions	CDI	24.1 (6.7); 27.1 (8.7)		Published	Dutch Organisation for Health Research and Development (Netherlands)
Topooco (2017)	Placebo (supported); Guided (interactive)	35; 35	BDI > 14	17.5 (15–19)		Sweden (English)	Clinical	NR; 8 Individual CBT modules and 8 individual therapy‐weekly sessions	BDI, MFQ	28.8(7.9), 35.2 (9.4); 31.6 (10), 36 (10.7)	12	Published	Swedish Central Bank; Queen Silvia's Jubilee Fund, Sweden‐America Foundation; Swedish Society of Medicine; Swedish Psychotherapy Society (Sweden)
Topooco (2019)	Placebo (supported); Guided (interactive)	37; 33	BDI > 14	17 (15–19)		Sweden (English)	Community	8‐weekly sessions; 8 modules completed in own time and 8‐weekly therapy sessions	BDI	32.3 (10.2); 33.1 (9.4)		Published	Queen Silvia's Jubilee Fund; Swedish Central Bank (Sweden)
Vostanis (1996)	Placebo (supported); Individual	28; 29	MFQ > 16	12.7 (8–17)	Depressed per DSM‐III‐R	UK (English)	Primary Care	9 fortnightly sessions spread over a maximum period of 6 months; 9 fortnightly session spread over a maximum period of 6 months	MFQ	28.6 (14.4); 33.4 (12.2)	9	Published	Merck Research Fund; Queen Elizabeth Psychiatric Hospital Trustees' Fund (UK)
Vuthiarpa (2012)^a^	TAU; Group	37; 37	CES‐D >16, <24	15.5 (15–16)		Thailand (English)	School	NR; 12‐weekly 1‐h sessions	CES‐D	19.74 (2.52); 19.66 (2.57)		Published	King Prajadhipok and Queen Rambhai Barni Memorial Foundation (Thailand)
Wijnhoven (2014)	None; Group	52; 50	CDI > 16	13.3 (11–15)		The Netherlands (English)	School	N/A; 8‐weekly 50‐min sessions	CDI, CES‐D	19.31 (6.74), 24.68 (11.07); 15.82 (6.62), 19.53 (9.8)	6	Published	GGz Oost‐Brabant; The Olim Foundation (Netherlands)
Woods (2011)	TAU; Group	12; 12	CDI > 63	14 (NR)		New Zealand (English)	School	NR; 8‐weekly 90‐min sessions	CDI	26.17 (4.32); 22.92 (6.63)	12	Published	Not reported
Wright (2019)	Placebo (self‐help); Unguided (interactive)	69; 70	MFQ > 20	15 (12–18)		UK (English)	Clinical	Weekly sessions, flexible for the participant; Weekly sessions, flexible for the participant	BDI, MFQ	16, 35.3 (6.6, 9.9); 18, 37.5 (6.9, 9.2)	12	Published	National Institute for Health Research (UK)
Yu (2000)	None; Group	116; 104	CDI (top 25%) FES (top 25%)	11.8 (9–14)		China (English)	School	N/A; 10‐weekly 2‐h sessions	CES‐D	16.72 (9.29); 17.44 (9.47)		Unpublished	Not reported – PhD

Abbreviations: BDI, Beck's Depression Inventory; CBT, cognitive behavioural therapy; CESD/CES‐D, Centre for Epidemiologic Studies Depression Scale; CES‐DC, CESD for Children; CESD‐R, CESD Revised; CDI, Children's Depression Inventory; CDRS‐R, Children's Depression Rating Scale‐Revised; FES, Family Environment Scale; DSM, Diagnostic and Statistical Manual of Mental Disorders; Group, group CBT; Guided, guided self‐help; HRSD, Hamilton Rating Scale for Depression; Individual, individual CBT; K‐SADS, Children's Schedule for Affective Disorders and Schizophrenia; MASQ, Mood and Anxiety Symptom Questionnaire; MDD, major depressive disorder; MFQ, Mood and Feelings Questionnaire; N/a, not applicable; NR, not reported; PHQ:−9 Patient Health Questionnaire; RADS/RCDS, Reynolds Adolescent/Child Depression Scale; Remote, remotely‐delivered CBT; QIDS‐A‐SR, Quick Inventory of Depressive Symptomatology – Adolescent Version; SD, standard deviation; SDS, Zung Self‐Rating Depression Scale; SMFQ, Short MFQ; TAU, treatment‐as‐usual; Unguided, unguided self‐help.

^a^ Cluster‐randomised controlled trial. All other studies are parallel group, individually‐randomised controlled trials.

##### Design

Eight included studies were cluster randomised trials (Bella‐Awusah, 2016; Idsoe, 2019; Kerfoot, 2004; Kobak, 2015; Sheffield, 2006; Srivastava, 2015; Stallard, 2012; Vuthiarpa, 2012). The remaining 60 studies were parallel‐group individually randomised controlled trials. There were 14 multi‐arm studies, although more than two arms were included in the analysis for only seven studies (other multi‐arm studies were Brent, 1997; Clarke, 1999; Hamamci, 2006; Kahn, 1988; Lewinsohn, 1990; Parks, 2013; Rossello, 1999; and Sheffield, 2006).

##### Sample size

The sample size ranged from 5 to 229 per node. Cluster trials had as many as 392 participants in one node.

##### Study setting

Studies were mostly conducted in schools (28) or universities (6). Other settings included clinical settings (9), primary care (13), community (3), home (2), education counselling centre (1) and penal settings (3). Three studies did not report the settings.

Trials were conducted across 21 different countries. The most common countries were USA (29), New Zealand (5), UK (4), Canada (3), Iran (3) and Thailand (3). Two studies each were conducted in China, India, Malaysia, Nigeria, the Netherlands, and Sweden. There were single studies from Australia, Belgium, Chile, Hong Kong, Japan, Norway, Puerto Rico, Spain and Turkey. Most included studies were written in English, except for one written in French (Marcotte, 1993) and one written in Persian (Rajabi, 2018).

##### Participants

**Figure 3:**
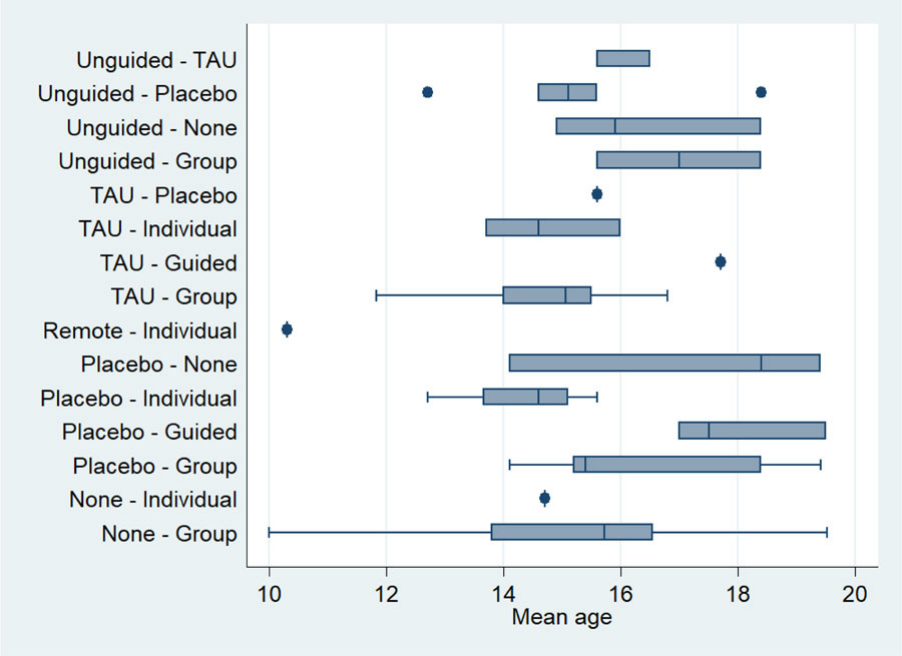
Boxplot of the distribution of mean age in individual trials across comparisons.

The mean age of participants ranged from 10 to 19.5 years. 12 studies had a mean age of participants between 10 and 13, 32 between 14 and 15 and 19 between 16 and 19 years. Five studies did not report the mean age. The distribution of mean age across trials by pairwise comparison of nodes is presented in Figure 3.

The proportion of females ranged from 7% to 100%. 17 studies had less than 50% females, 16 studies had 70% or more females. On average, 60% of participants were female. Four studies did not report participant gender.

Studies assessed inclusion using a range of different tools. Most commonly, participants were assessed using the Beck Depression Inventory (BDI) or the Child Depression Inventory (CDI). Thirteen studies did not assess participants for inclusion using a measure of depression but were included in this review as the mean baseline scores met our criteria.

The cut‐off for all scales used in the review are reported in Table 1.

**Figure 4:**
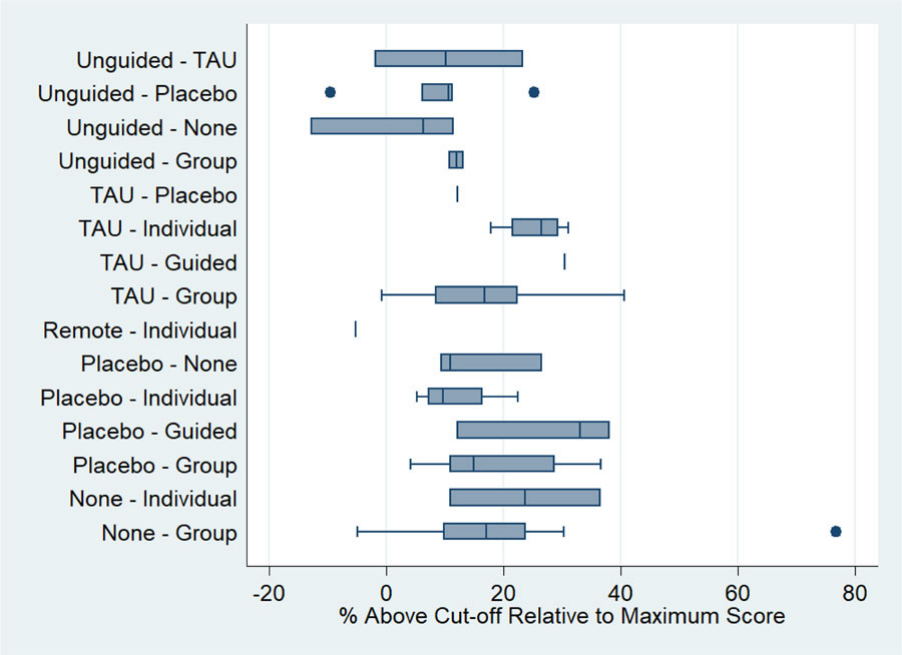
Boxplot of pretest depression scores in individual trials across comparisons. The score from each trial is represented as a percent above the cut‐off score for elevated depression symptoms relative to the scale's maximum possible score. For example, if a trial had a pretest score of 30 on the Beck Depression Inventory Second Edition (cut‐off of 14 and maximum possible score of 63), this would translate to a value of (30 – 14)/(63 – 14) ∗ 100 = 33%. Note that some trials have a value below the cut‐off (<0%) because they used multiple scales within the trial, and the scale prioritised for the review (based on frequency of scales used across all studies), may not have been the scale used to determine patient eligibility in the study.

Baseline severity was investigated by representing each trial's baseline score as a percent above the cut‐off score for elevated depression symptoms relative to the scale's maximum possible score and evaluating within and across comparisons. This value is similar to the percent of maximum possible value, except it reflects the observed score relative to the scale's cut‐off rather than the minimum value (Cohen, 2010). Severity appeared to be heterogenous within some comparisons, but was not a clear threat to transitivity (Figure 4).

##### Interventions

Amongst the 68 included studies, in terms of active treatment delivery models, 12 included therapist‐delivered CBT in face‐to‐face individual sessions (individual CBT), 43 included therapist‐delivered CBT in group sessions (group CBT) and one study included therapist‐led CBT delivered remotely (remote CBT). The number of therapist‐delivered sessions for these active interventions ranged from 4 to 16, the frequency of delivery from once every 2 weeks to twice a week and the duration of each session from 20 min to 2 h. Group sizes for group CBT ranged from 2 to 20, but were more commonly around 6 to 10. Thirteen studies evaluated unguided self‐help and four studies evaluated self‐help with therapist support (or guided self‐help). The number of therapist‐delivered sessions in the guided self‐help models ranged from 1 to 8. The number of self‐help modules across these two categories ranged from 4 to 12. The self‐help interventions were further classified based on level of interactivity for the purposes of sensitivity analysis. Seven studies of unguided self‐help and three studies of guided self‐help were interactive, and six studies of unguided self‐help and the self‐help component of guided self‐help in one study were not interactive.

Comparison interventions included no intervention/waitlist (30 studies; 25 waitlist, 5 no treatment), TAU (20) and placebo (21). Details about TAU were provided by 18 studies and were described as: freedom to continue pre‐existing intervention or seek new assistance (4), self‐help/written information (3), school nurse/counsellor (2), routine psychiatric interventions (1), therapy for depression (1), pharmacotherapy plus various therapy (1), counselling (1), vocational training (1), sessions with social worker (1), and various/self‐selected (3). Placebos were classified as self‐help (6), pill (1) and professionally supported (14). No systematic differences in dose of treatment were detected between interventions when delivered in trials with inactive control groups and trials with active controls.

There was 83% agreement between reviewers in initial node assignment (kappa 0.79 [95% CI: 0.71 to 0.86]). Nodes from one study (Sheffield, 2006) were excluded from this analysis as the node assignment was decided through discussion. In five cases, the final node classification ended up being completely different from the reviewers' initial assignment after review and discussion. In one case, the reclassification involved active treatment (group CBT to guided self‐help), while the remaining cases involved reclassifying control nodes to another control.

##### Outcomes

Two studies did not use self‐reported measures of depression and were thus not included in the quantitative analysis of the primary outcome (Rohde, 2014a, 2014b). Additionally, four studies were excluded from the analysis because we did not have suitable data to calculate the mean difference on the primary outcome at post‐intervention and were unable to obtain the required data from the authors. The first, Stikkelbroek (2020), assessed individual CBT vs TAU and reported improvements in both conditions over time but no significant differences between conditions in self‐reported depressive symptoms at post‐treatment or 6‐month follow‐up. Similarly, Kobak (2015) compared individual CBT with TAU and found a reduction in depression in both groups and no significant difference in outcomes between trial arms. Lamb (1998) compared school‐based group CBT with no treatment and also found reductions in depressive symptoms in both arms, with no significant differences between arms. Finally, Briere (2019) compared group CBT and an educational brochure in secondary schools and found significant differences, with greater reduction in symptoms of depression in group CBT compared to control at post‐treatment, but no significant differences between arms at 6‐month follow‐up.

Of the 62 studies included in the quantitative analysis for the primary outcome at post‐intervention, the most common measure was the Center for Epidemiologic Studies Depression Scale (CES‐D). Other measures used and included in this review are BDI, CDI, Mood and Feelings Questionnaire (MFQ), and Reynolds Adolescent Depression Scale (RADS). One study each used the Short MFQ, Patient Health Questionnaire (PHQ‐9), Zung Self‐Rating Depression Scale (SDS), and Mood and Anxiety Symptom Questionnaire (MASQ). For all measures, a lower score indicates a more beneficial outcome; no reversal of scale direction was necessary for combining data.

Just under a third of studies (19) followed‐up participants between six and twelve months after post‐intervention assessments.

62 studies were included in the secondary analysis of the measure attrition as a proxy for acceptability.

#### 6.1.3 Excluded studies

Reasons for exclusion were:

- Wrong study design: 35
- Wrong population: 198
- Wrong intervention: 24
- Wrong outcomes: 5
- Wrong comparator: 14
- Wrong analysis: 44

Where multiple criteria were not met, the first reason appearing in the above list is mentioned. These are reported in the PRISMA figure.

The most common reason was study population, as abstracts often did not mention the age group of participants and many studies included in full text screening were found to be focused on adults (e.g., Christensen, 2004; Richards, 2003; Salamanca‐Sanabria, 2020). Other studies were excluded because participants may have had other mental health conditions, not necessarily clinical levels of depression (e.g., Calear, 2009; Weisz, 2012), or because participants did not score in the clinical range on validated measures of depression (e.g., Stallard, 2010; Weisz, 2009; Whittaker, 2017).

Studies that reported on the wrong outcome reported results for outcomes such as: depression diagnosis, suicidal ideation, QALYs, and cognitive styles.

**Ongoing Studies**

Additionally, ten studies that appeared relevant were classified as ongoing or awaiting classification when more information becomes available.

### 6.2 Risk of bias in included studies

**Figure 5:**
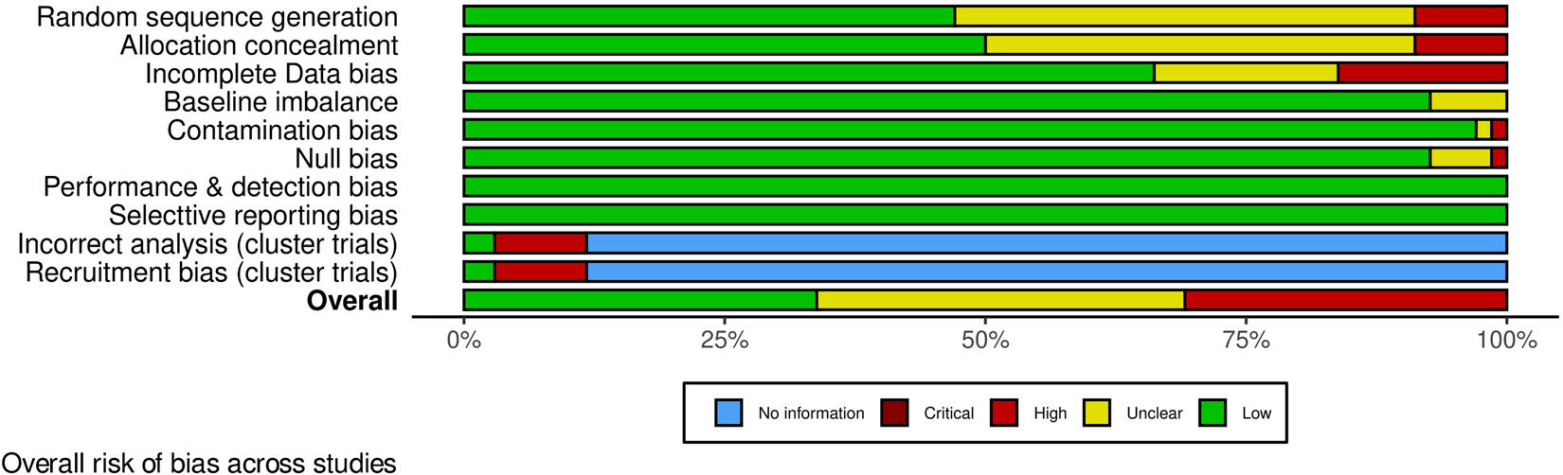
Overall risk of bias across studies.

Figure 5 shows the overall risk of bias across studies by the different domains. Figure 6 lists the risk of bias individually for each study. These were created using https://mcguinlu.shinyapps.io/robvis/.

Figure 6Risk of bias summary: review authors' judgements about each risk of bias item for each included study.

**Figure:**
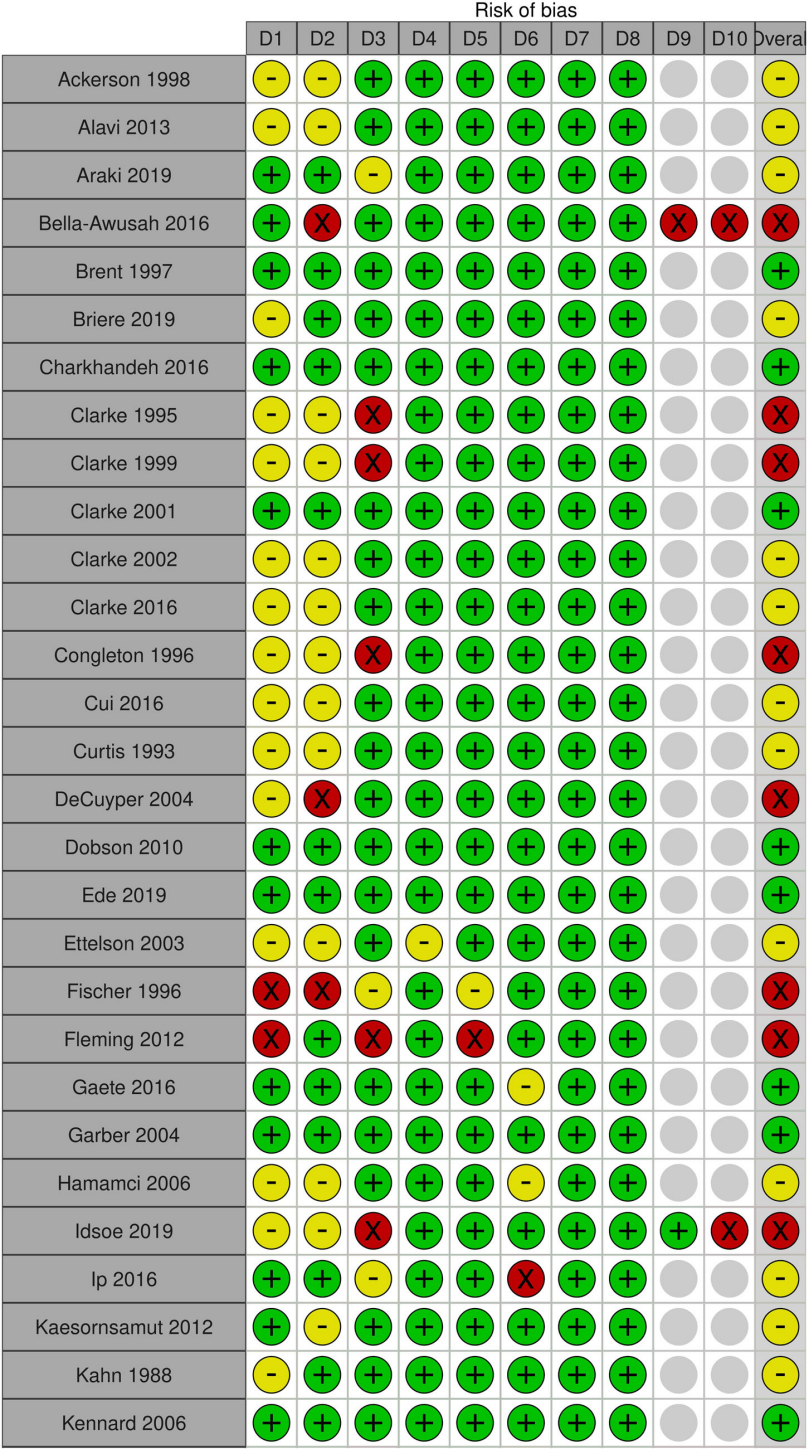

**Figure:**
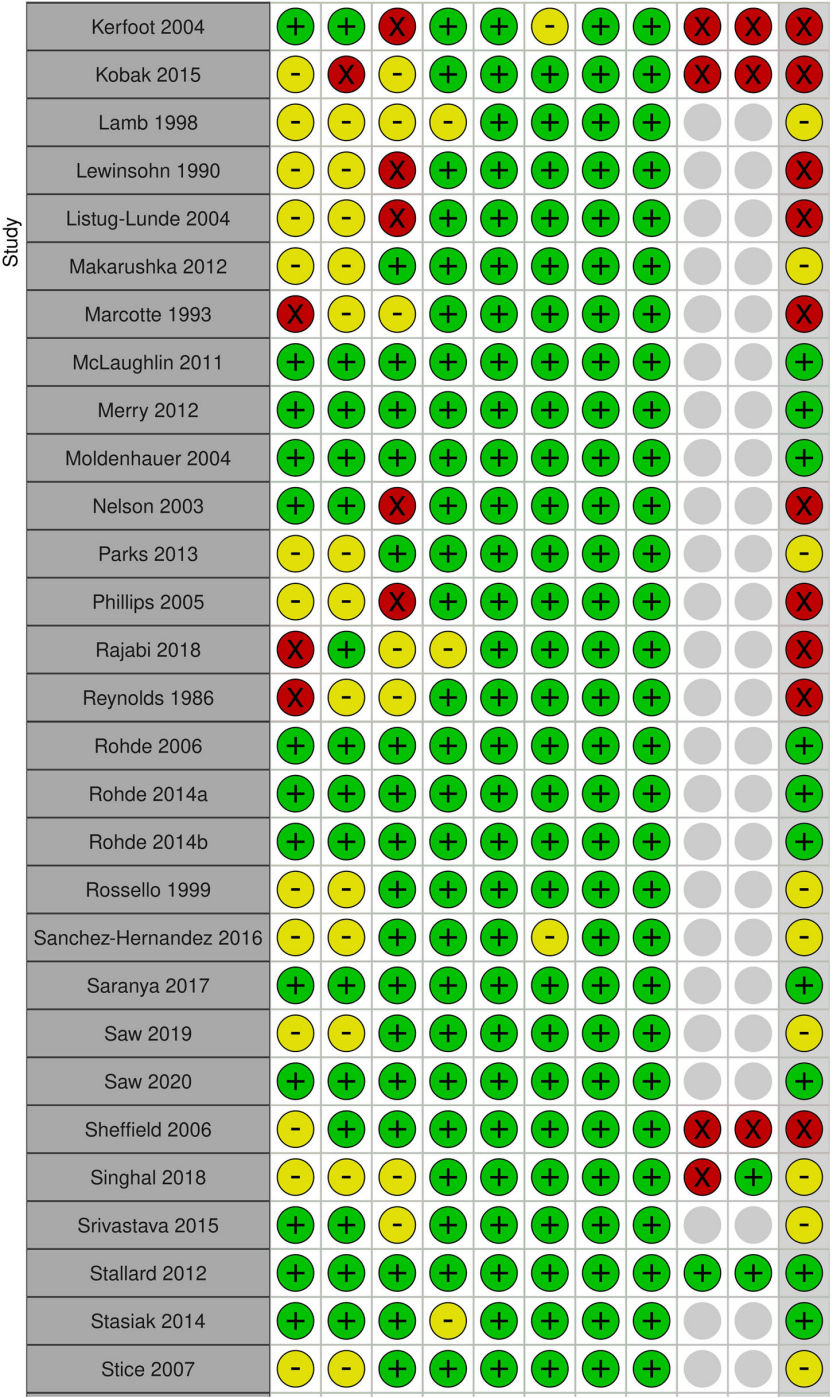

**Figure:**
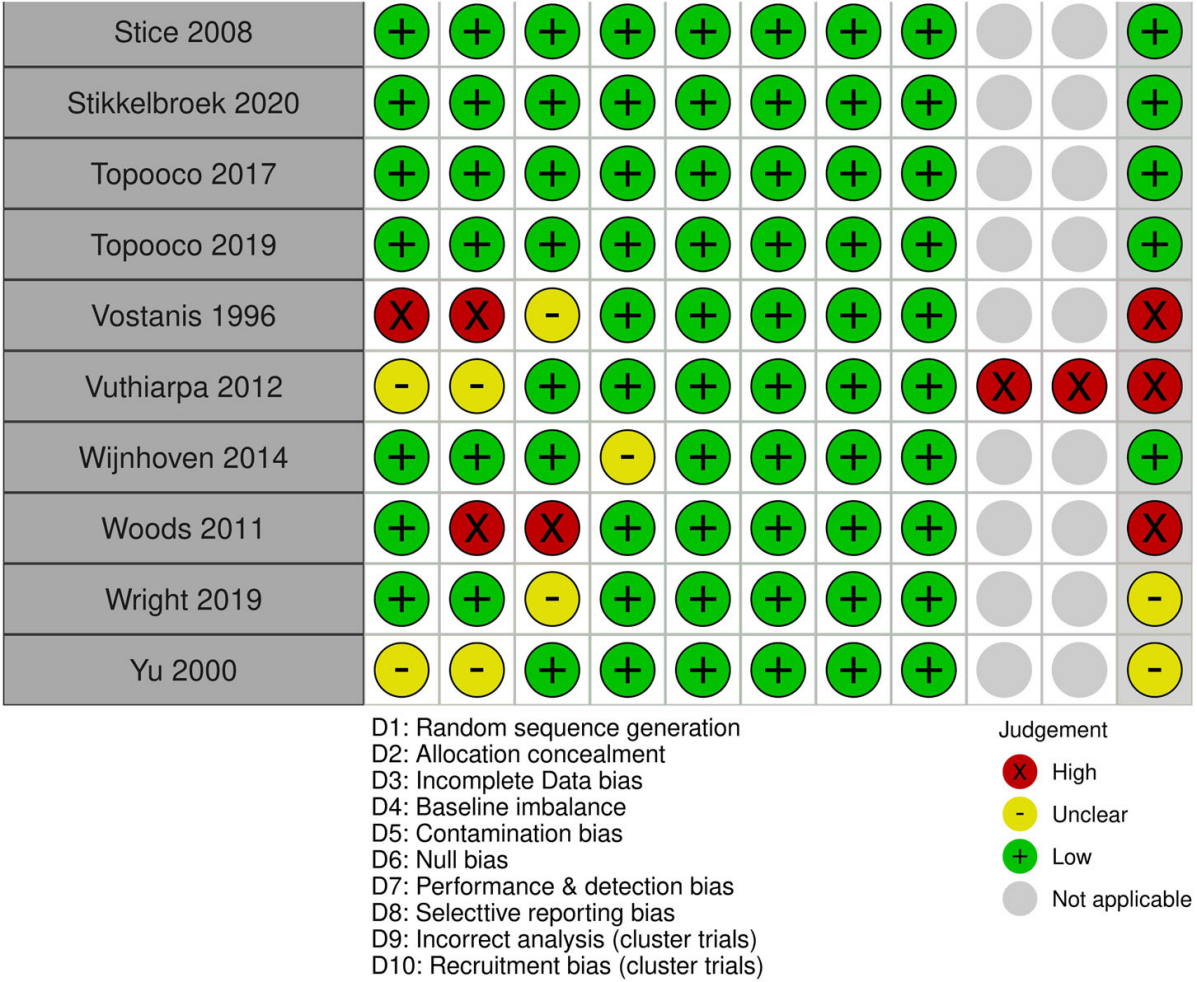

The risk of bias was low across all domains for 23 studies. 24 studies had some concerns and the remaining 21 were assessed to be at high risk of bias.

#### 6.2.1 Allocation

We assessed allocation bias through random sequence generation and allocation concealment. In addition, for cluster trials we assessed recruitment bias. Random sequence generation was considered adequate in less than half of the studies (32), whereas allocation concealment was considered adequate in 34 studies. In terms of recruitment bias, only two of the seven cluster trial studies were rated as low risk.

Across the three types of bias, only 28 studies were rated low risk. There were some concerns with 26 studies and 14 were rated as high risk. Reasons for a high risk rating included assessing eligibility/gaining consent post randomisation, reassigning participants after randomisation, small sample (only two schools in a cluster study), and poor randomisation techniques (e.g., sequential assignment).

#### 6.2.2 Blinding

Blinding of participants is not always possible for social interventions, and thus performance bias was rated low across the studies. Only self‐reported measures were included in quantitative analysis and thus detection bias is also low across the studies.

#### 6.2.3 Incomplete outcome data

45 studies had no concerns in terms of incomplete data, whereas 12 had some concerns. The remaining 11 were assessed to be at high risk of bias due to incomplete data. Reasons included not undertaking intention‐to‐treat analysis (e.g., a minimum attendance required to be included in analysis), high dropout and lack of imputing missing data when there was a considerable level of missing data.

#### 6.2.4 Selective reporting

There were no concerns with selective reporting in any study.

#### 6.2.5 Other potential sources of bias

We also assessed baseline imbalance, null bias, contamination bias, and incorrect analysis for cluster trials.

Five studies did not report baseline equivalence and were rated to be at unclear risk, leaving 63 rated as low risk.

One study noted poor completion to be a concern, with only 10% having completed the intervention; this study was rated to be at high risk of null bias. Four other studies noted some concerns and were rated as unclear risk. The remaining 63 studies were assessed to be at low risk of null bias.

In terms of contamination bias, one study was assessed to be at high risk as one participant from the control group received the intervention. Another study was considered to be at unclear risk because some control group participants were reported to have received some intervention sessions. However, these participants were not included in the research protocol (considered in selection and attrition bias). The remainder were assessed to be at low risk as there were no concerns reported for other studies in terms of contamination bias.

Of the eight cluster trials, six were assessed to be at high risk of bias due to incorrect analysis because there was no adjustment for intracluster correlation. Sheffield (2006) did use a multi‐level model including the cluster unit and time to account for clustering; however, the reporting of this analysis was insufficient to include in quantitative synthesis, and arm‐level results had to be adjusted using imputed ICCs, and was therefore rated at high risk.

#### 6.2.6 Publication bias

**Figure 7:**
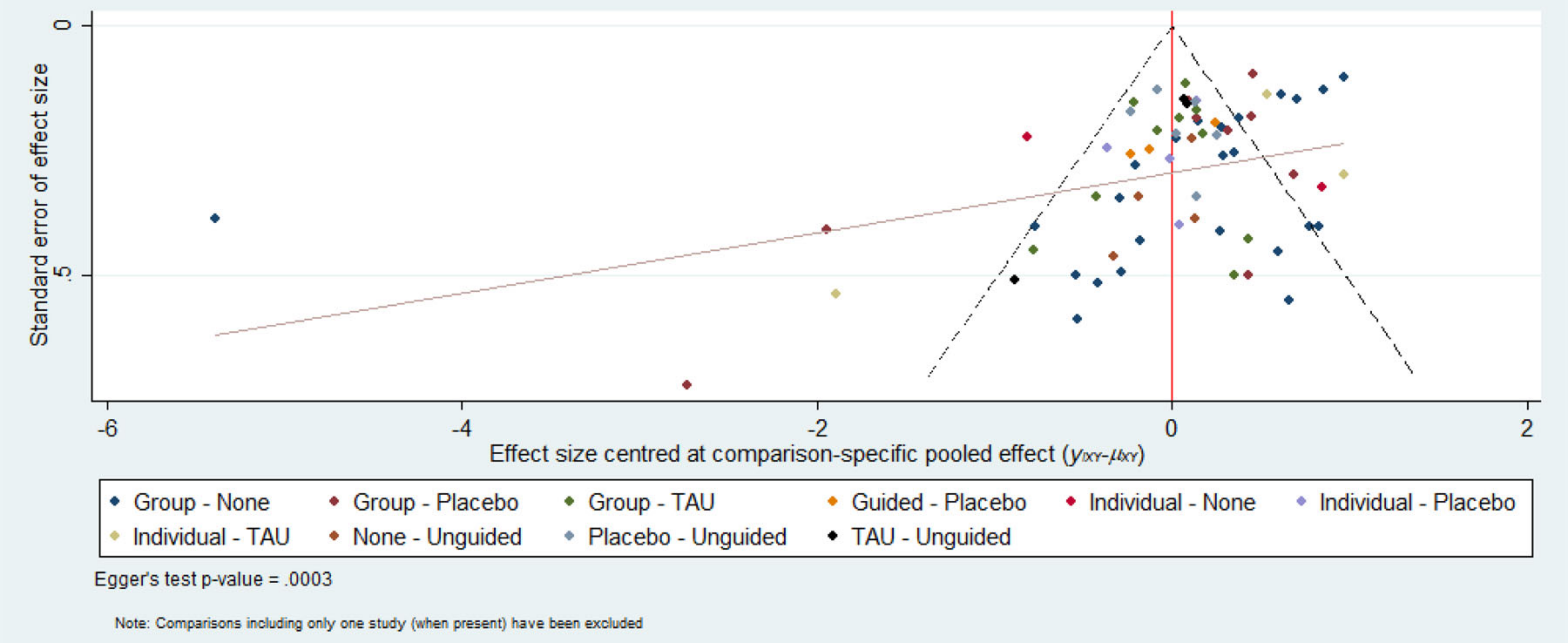
Comparison‐adjusted funnel plot of active versus inactive comparisons for post‐test depression score. A prediction line representing the linear regression of the effect size standard error on the adjusted effect size is included. Egger's test for small‐study effects is statistically significant (*p* < 0.001).

Publication bias is suspected amongst studies comparing active and inactive control treatments. The comparison‐adjusted funnel plot suggests that smaller studies tended to estimate greater improvements in post‐treatment depression score from active treatments than larger studies do, with the Egger's test for small‐study effects being statistically significant (*p* < 0.001) (Figure 7).

### 6.3 Effects of interventions

#### 6.3.1 Network meta‐analysis of cognitive behavioural therapy approaches

##### Primary outcomes

###### Depressive symptoms final score at post‐intervention

**Figure 8:**
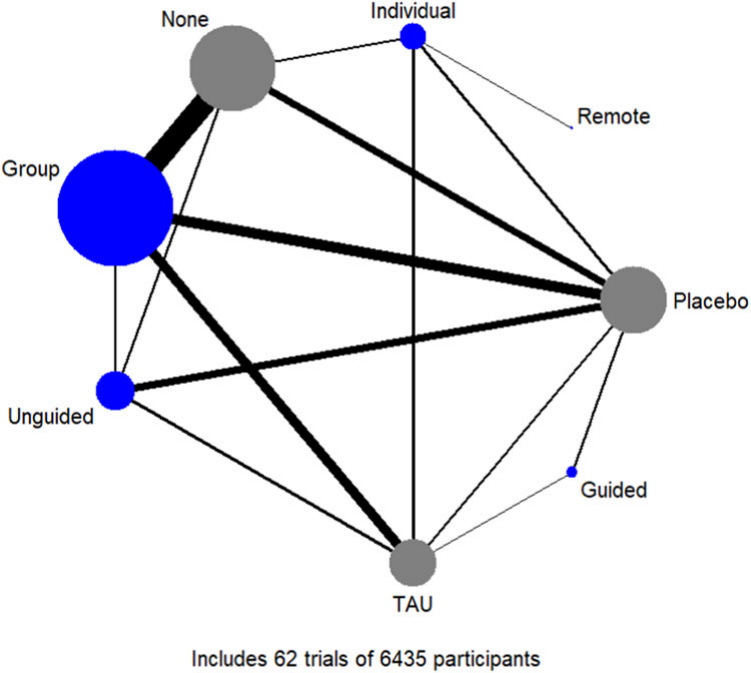
Network plot for post‐test depression score. Nodes are weighted by the number of patients randomised to that intervention, and edges are weighted by the number of trials comparing the two interventions.

Sixty‐two RCTs (representing 6435 participants) were included in the pairwise and network meta‐analyses for post‐intervention depressive symptom score. Figure 8 presents a network plot showing how treatments were compared across the included RCTs. All pre‐specified treatment and control categories were represented by at least one RCT. The number of RCTs comparing two interventions ranged between 1 and 24. Group CBT was the most commonly evaluated active condition and no intervention was the most commonly used control comparator. The network geometry indicates that guided self‐help has not been directly evaluated against another active treatment and rCBT has only been compared to individual CBT.

Of the seven cluster‐randomised trials included in the analysis, two (Idsoe, 2019; Stallard, 2012) reported ICCs derived from the analysis (range of 0.025 to 0.099), and these values were used to adjust the standard error. One (Kerfoot, 2004) reported that it assumed an ICC of 0.3 in its sample size calculation, and we used this value for adjustment. The remaining trials did not report ICCs, and because of a generally similar setting, we imputed the value reported in Stallard (2012) (0.025) for the adjustment.

**Figure 9:**
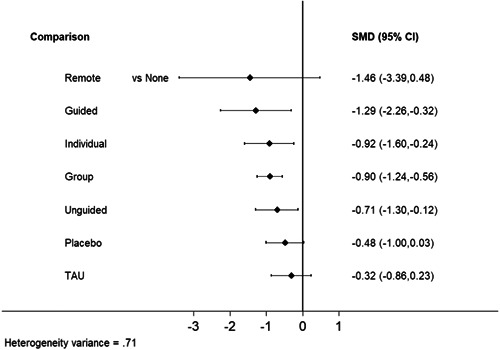
Forest plot of posttest depression score network estimates comparing interventions and other controls with no intervention.

**Figure 10:**
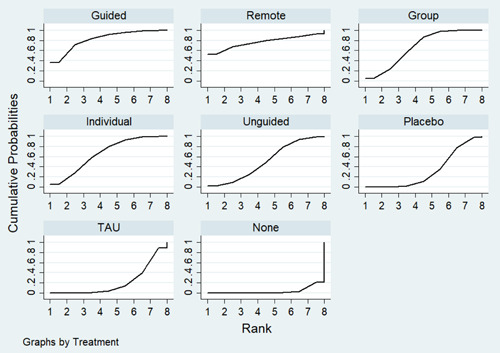
Cumulative ranking probabilities for each intervention in the post‐test depression score network. Rank indicates the cumulative probability of the intervention being the best method, the second best, the third best, and so forth.

**Figure 11:**
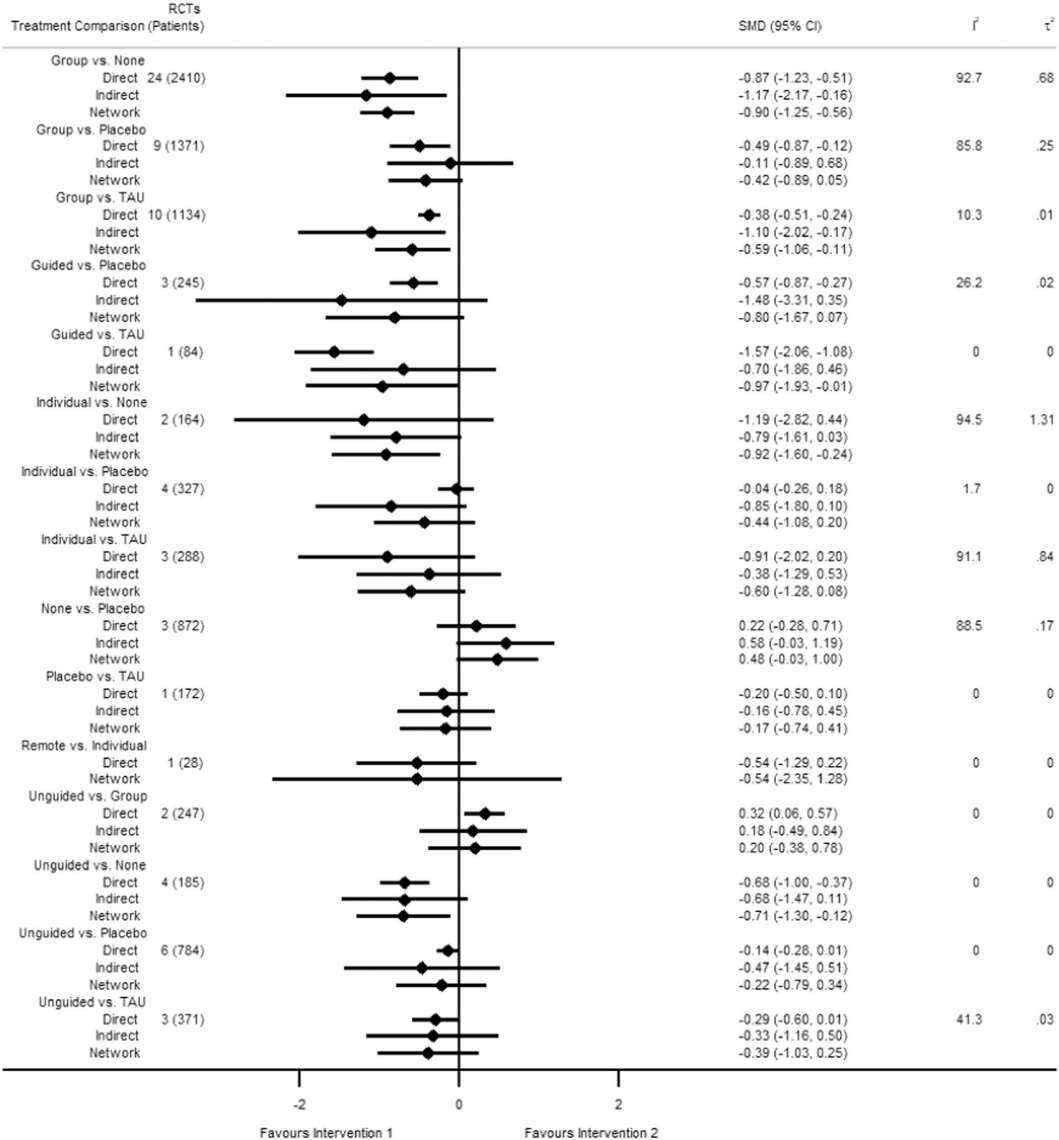
Forest plot of direct estimates of comparative effectiveness of interventions, with corresponding indirect and network estimates, for post‐test depression score. Intervention 1 is the treatment listed to the left in the treatment comparison column, and Intervention 2 is the treatment on the right. Sample size (number of randomised controlled trials and patients) and heterogeneity measures (*I* ^2^ and *τ* ^2^) are provided for the direct estimates.

All active interventions except for remote CBT demonstrated superiority compared to no intervention, with the effectiveness ranging from an SMD of −1.29 (95% CI: −2.26 to −0.32) for guided self‐help to −0.71 (95% CI: −1.30 to −0.12) for unguided self‐help (Figure 9, Table 3). Fewer demonstrated superiority compared to TAU, and none to the placebo condition. In terms of comparative effectiveness, no active treatment demonstrated superiority over another, with the certainty of evidence ranging from low to very low. The highest and lowest ranking interventions were guided self‐help (SUCRA 83%) and unguided self‐help (SUCRA 51%), respectively (*very low certainty in treatment ranking*) (Figure 10, Table 3). Pairwise meta‐analytic findings were generally similar to those of the network meta‐analysis (Figure 11).

**Table 3:** League table for post‐test depression score.

**Guided**	‐	‐	‐	‐	‐	‐	‐
0.17 (−1.92, 2.26)	**Remote**	‐	−0.54 (−1.29, 0.22)	‐	‐	‐	‐
−0.38 (−1.33, 0.56)	−0.55 (−2.48, 1.38)	**Group**	‐	−0.32 (−0.57, −0.06)	−0.49 (−0.87, −0.11)	−0.38 (−0.51, −0.24)	−0.87 (−1.23, −0.51)
−0.37 (−1.41, 0.68)	−0.54 (−2.35, 1.28)	0.02 (−0.65, 0.68)	**Individual**	‐	−0.04 (−0.26, 0.18)	−0.91 (−2.02, 0.20)	−1.19 (−2.82, 0.44)
−0.58 (−1.58, 0.43)	−0.75 (−2.72, 1.22)	−0.20 (−0.77, 0.38)	−0.21 (−0.98, 0.56)	**Unguided**	−0.14 (−0.28, 0.01)	−0.29 (−0.60, 0.01)	−0.68 (−1.00, −0.37)
−0.80 (−1.67, 0.07)	−0.97 (−2.89, 0.95)	−0.42 (−0.89, 0.05)	−0.44 (−1.07, 0.20)	−0.22 (−0.79, 0.34)	**Placebo**	−0.20 (−0.50, 0.10)	‐
−0.97 (−1.93, −0.01)	−1.14 (−3.07, 0.79)	−0.59 (−1.06, −0.11)	−0.60 (−1.28, 0.07)	−0.39 (−1.03, 0.25)	−0.17 (−0.74, 0.40)	**TAU**	‐
−1.29 (−2.26, −0.32)	−1.46 (−3.39, 0.48)	−0.90 (−1.24, −0.56)	−0.92 (−1.60, −0.24)	−0.71 (−1.30, −0.12)	−0.48 (−1.00, 0.03)	−0.32 (−0.86, 0.23)	**None**
**SUCRA**							
82.6%	76.9%	67.1%	66.0%	50.9%	31.9%	21.0%	3.5%

*Note*: Standardised mean differences (95% confidence intervals) for each comparison in the analysis of post‐test depression score. Estimates below the diagonal represent network meta‐analysis results while those above represent pairwise meta‐analysis results. Below the diagonal, standardised mean difference <  0 favours the intervention in the column (above the diagonal, <0 favours the intervention in the row). Values are underlined when one intervention demonstrated superiority based on the confidence interval. Interventions are ordered based on the SUCRA values.

Abbreviations: CBT, cognitive behavioural therapy; Group, group CBT; Guided, guided self‐help; Individual, individual CBT; Remote, remotely‐delivered CBT; SUCRA, surface under the cumulative ranking area curve; TAU, treatment‐as‐usual; Unguided, unguided self‐help.

Based on *χ* ^2^ and *I* ^2^ statistics, heterogeneity within pairwise comparisons ranged from not important to considerable (Figure 11). At the network meta‐analytic level, the extent of heterogeneity appeared to be moderate (*τ* ^2^ = 0.71) compared to the empirical distribution for non‐pharmacological interventions with mental health outcomes measured on a continuous scale (median *τ* ^2^ = 0.058, 95% range = 0.001 to 2.58) (Rhodes, 2015). The loop‐specific approach showed incoherence for the guided self‐help, placebo, and TAU closed loop of evidence (incoherence factor [IF] = 0.803, 95% CI: 0.05 to 1.56, *p* = 0.037). The source of incoherence in this loop appears to be Saranya 2017, which is the only study comparing guided self‐help and TAU, was conducted in an incarcerated population, and found a fairly large effect size for the treatment. While the node‐splitting approach did not demonstrate clear incoherence (incoherence *p*‐values ranged from 0.209 to 1.000), the direct and indirect estimates were notably different for individual CBT versus placebo, with the direct estimate suggesting no important difference between the groups (SMD −0.04, 95% CI: −0.26 to 0.18) and the indirect estimate indicating there may be an important difference(SMD −0.85, 95% CI: −1.79 to 0.10). The design‐by‐treatment interaction model did not detect incoherence (*p* = 0.973).

###### Depressive symptoms final score at 6 to 12 months follow‐up

Nineteen RCTs (representing 3260 participants) were included in the pairwise and network meta‐analyses for 6 to 12 months follow‐up depressive symptom score.

**Figure 12:**
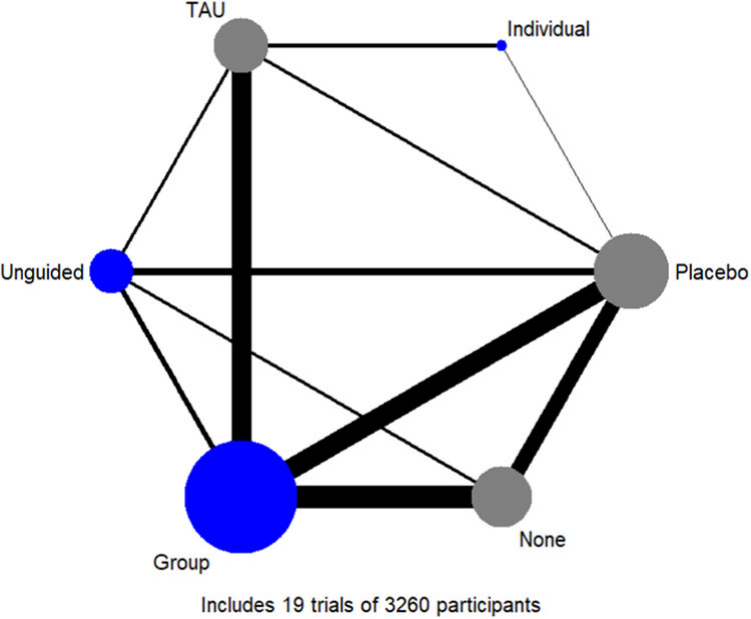
Network plot for 6‐ to 12‐month follow‐up depression score. Nodes are weighted by the number of patients randomised to that intervention, and edges are weighted by the number of trials comparing the two interventions.

Figure 12 presents a network plot showing how treatments were compared across the included RCTs. Neither guided self‐help nor remote CBT were evaluated in the RCTs for this time point. The number of RCTs comparing two interventions ranged between 1 and 7. Group CBT was the most commonly evaluated active condition and placebo was the most commonly used control comparator. The network geometry indicates that individual CBT has not been directly evaluated against another active treatment.

**Figure 13:**
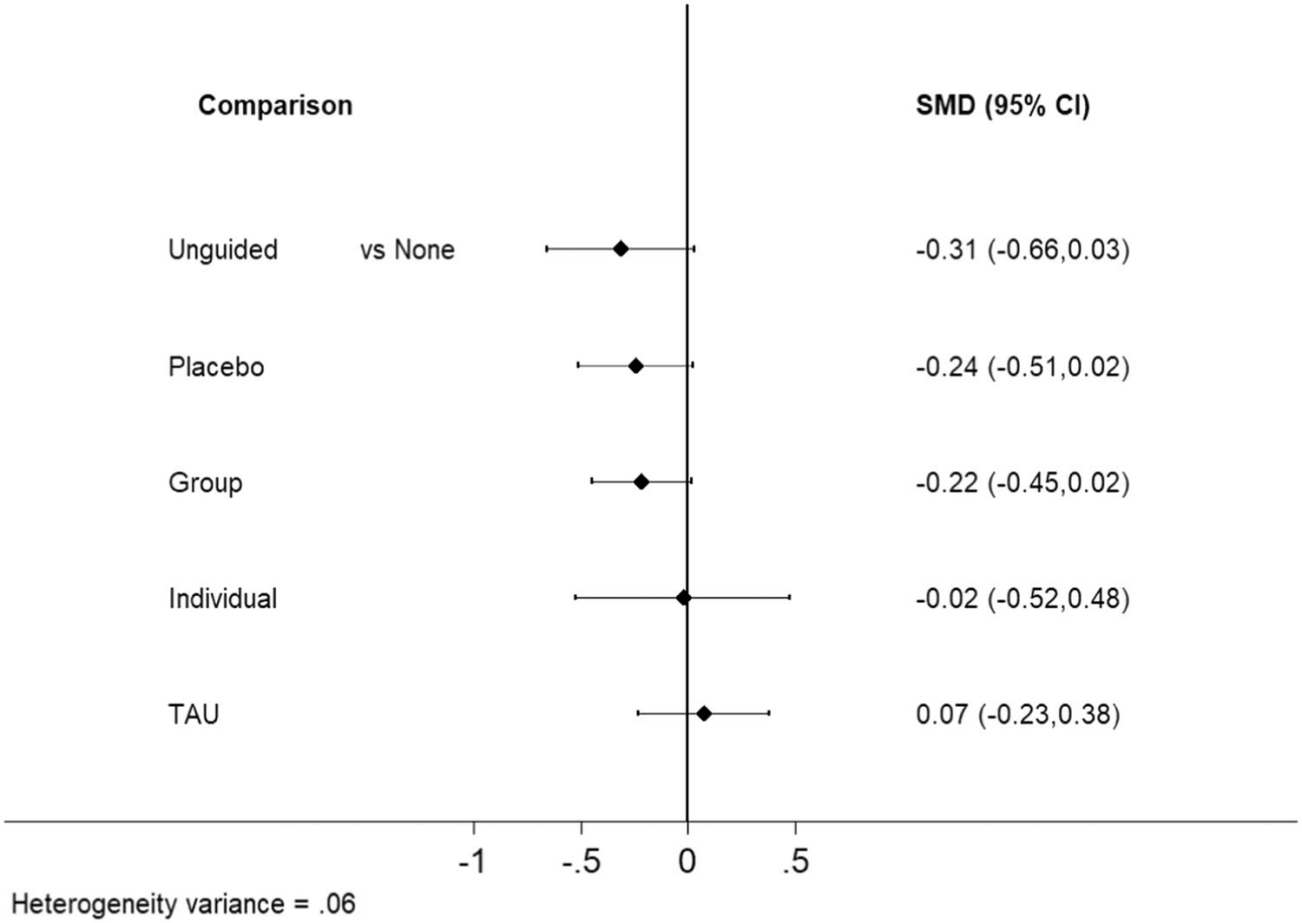
Forest plot of 6‐ to 12‐month follow‐up depression score network estimates comparing interventions and other controls with no intervention.

**Figure 14:**
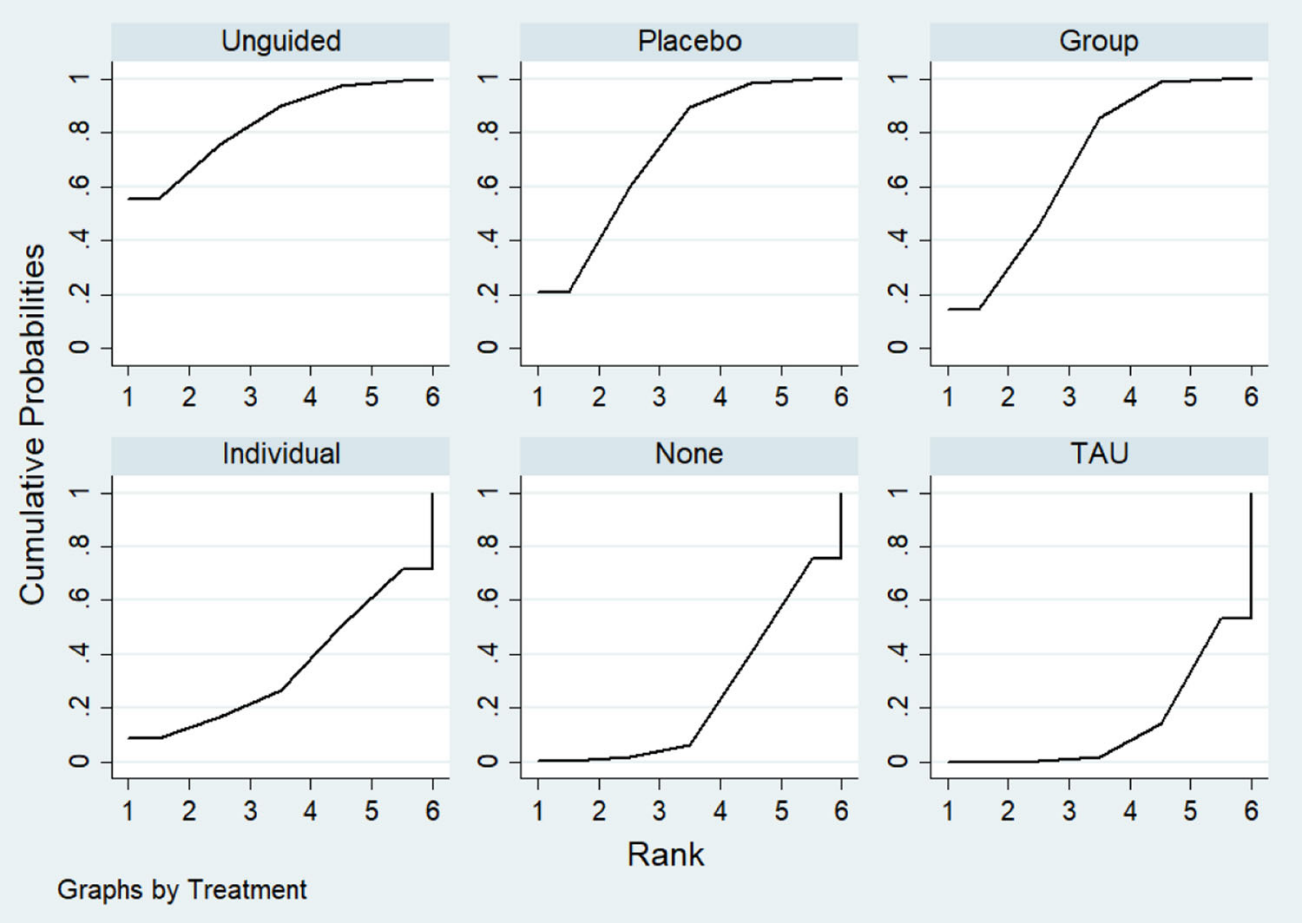
Cumulative ranking probabilities for each intervention in the 6‐ to 12‐month follow‐up depression score network. Rank indicates the cumulative probability of the intervention being the best method, the second best, the third best, and so forth.

**Figure 15:**
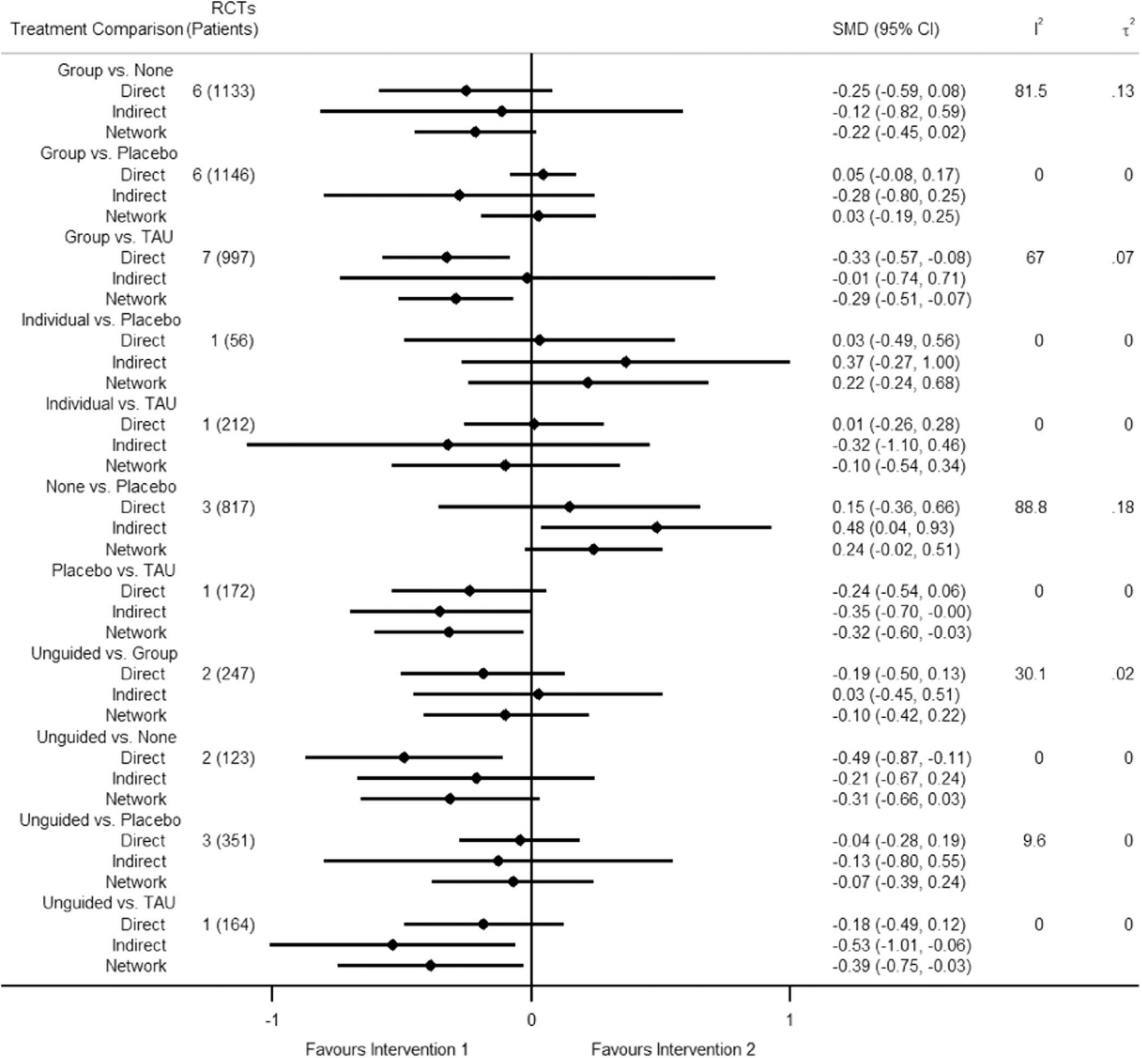
Forest plot of direct estimates of comparative effectiveness of interventions, with corresponding indirect and network estimates, for 6‐ to 12‐month follow‐up depression score. Intervention 1 is the treatment listed to the left in the treatment comparison column, and Intervention 2 is the treatment on the right. Sample size (number of randomised controlled trials and patients) and heterogeneity measures (*I* ^2^ and *τ* ^2^) are provided for the direct estimates.

The magnitude of effects appeared to be generally attenuated for 6‐ to 12‐month outcomes compared to posttest. No interventions demonstrated superiority to no intervention (Figure 13), although unguided self‐help (SMD −0.39, 95% CI: −0.75 to −0.03) and group CBT (−0.29, 95% CI: −0.51 to −0.07) both demonstrated superiority compared to TAU (Table 4). No active treatment demonstrated superiority over another. The highest and lowest ranking interventions were unguided self‐help (SUCRA 84%) and individual CBT (SUCRA 35%), respectively (Figure 14, Table 4). Pairwise meta‐analytic findings were similar to those of the network meta‐analysis (Figure 15).

**Table 4:** League table for 6‐ to 12‐month follow‐up depression scores.

**Unguided**	−0.04 (−0.28, 0.19)	−0.19 (−0.50, 0.13)	‐	−0.49 (−0.87, −0.11)	−0.18 (−0.49, 0.12)
−0.07 (−0.39, 0.24)	**Placebo**	−0.05 (−0.08, 0.17)	0.03 (−0.49, 0.56)	0.15 (−0.36, 0.66)	−0.24 (−0.54, 0.06)
−0.10 (−0.42, 0.22)	−0.03 (−0.25, 0.19)	**Group**	‐	−0.25 (−0.59, 0.08)	−0.33 (−0.57, −0.08)
−0.29 (−0.82, 0.24)	−0.22 (−0.68, 0.24)	−0.19 (−0.65, 0.27)	**Individual**	‐	0.01 (−0.26, 0.28)
−0.31 (−0.66, 0.03)	−0.24 (−0.51, 0.02)	−0.22 (−0.45, 0.02)	−0.02 (−0.52, 0.48)	**None**	‐
−0.39 (−0.75, −0.03)	−0.32 (−0.60, −0.03)	−0.29 (−0.51, −0.07)	−0.10 (−0.54, 0.34)	−0.07 (−0.38, 0.23)	**TAU**
**SUCRA**					
83.6%	73.8%	68.9%	34.8%	25.0%	13.9%

*Note*: Standardised mean differences (95% confidence intervals) for each comparison in the analysis of 6 to 12 month‐follow‐up depression score. Estimates below the diagonal represent network meta‐analysis results while those above represent pairwise meta‐analysis results. Below the diagonal, standardised mean difference  < 0 favours the intervention in the column (above the diagonal, < 0 favours the intervention in the row). Values are underlined when one intervention demonstrated superiority based on the confidence interval. Interventions are ordered based on the SUCRA values.

Abbreviations: CBT, cognitive behavioural therapy; Group, group CBT; Individual, individual CBT; SUCRA, surface under the cumulative ranking area curve; TAU, treatment‐as‐usual; Unguided, unguided self‐help.

Based on *χ* ^2^ and *I* ^2^ statistics, heterogeneity within pairwise comparisons ranged from not important to substantial (Figure 15). At the network meta‐analytic level, the extent of heterogeneity appeared to be small (*τ* ^2^ = 0.06) based on the empirical distribution for non‐pharmacological interventions with mental health outcomes measured on a continuous scale (median *τ* ^2^ = 0.058, 95% range = 0.001 to 2.58) (Rhodes, 2015). The loop‐specific (IF *p*‐values ranged from 0.228 to 0.944), node‐splitting (incoherence *p*‐values ranged from 0.186 to 0.759), and design‐by‐treatment interaction model (*p* = 0.979) approaches did not detect any incoherence.

##### Secondary outcomes

###### Acceptability of intervention

Sixty‐two RCTs (representing 7347 participants) were included in the pairwise and network meta‐analyses for intervention acceptability as measured by study attrition. Across these 62 studies, attrition rate was on average 11.2% at posttest. These 62 studies involved 130 intervention/control arms that can be classified as one of the nodes in this review. Attrition rate was calculated for each node and is reported in Table 5. Dropout from treatment was reported in only 13 studies. There was no dropout in one study, less than 5% in six studies, between 5% and 10% in 2 studies, and greater than 10% in the remaining 4 studies.

**Table 5:** Attrition by node.

Node	Average completion	Data from number of trial arms
No intervention	92.6%	27/30
Therapist‐delivered CBT – Group	89.4%	37/44
Placebo	88.6%	19/21
TAU	87.4%	18/21
Guided self‐help	87.4%	4/4
Unguided self‐help	87.1%	12/13
Individual CBT	84.3%	12/12
Remotely‐delivered CBT	70.0%	1/1
Across nodes	88.8%	130/146

**Figure 16:**
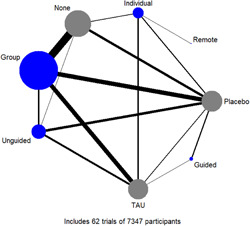
Network plot for intervention acceptability. Nodes are weighted by the number of patients randomised to that intervention, and edges are weighted by the number of trials comparing the two interventions.

Figure 16 presents a network plot showing how treatments were compared across the included RCTs. All pre‐specified treatment and control categories were represented by at least one RCT. The number of RCTs comparing two interventions ranged between 1 and 22. Group CBT was the most commonly evaluated active condition and no intervention was the most commonly used control comparator. The network geometry indicates that guided self‐help has not been directly evaluated against another active treatment and remote CBT has only been compared to individual CBT.

**Figure 17:**
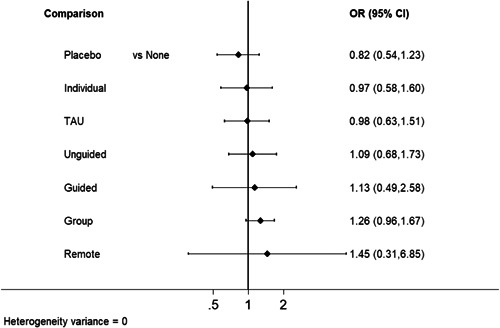
Forest plot of intervention acceptability network estimates comparing interventions and other controls with no intervention.

**Figure 18:**
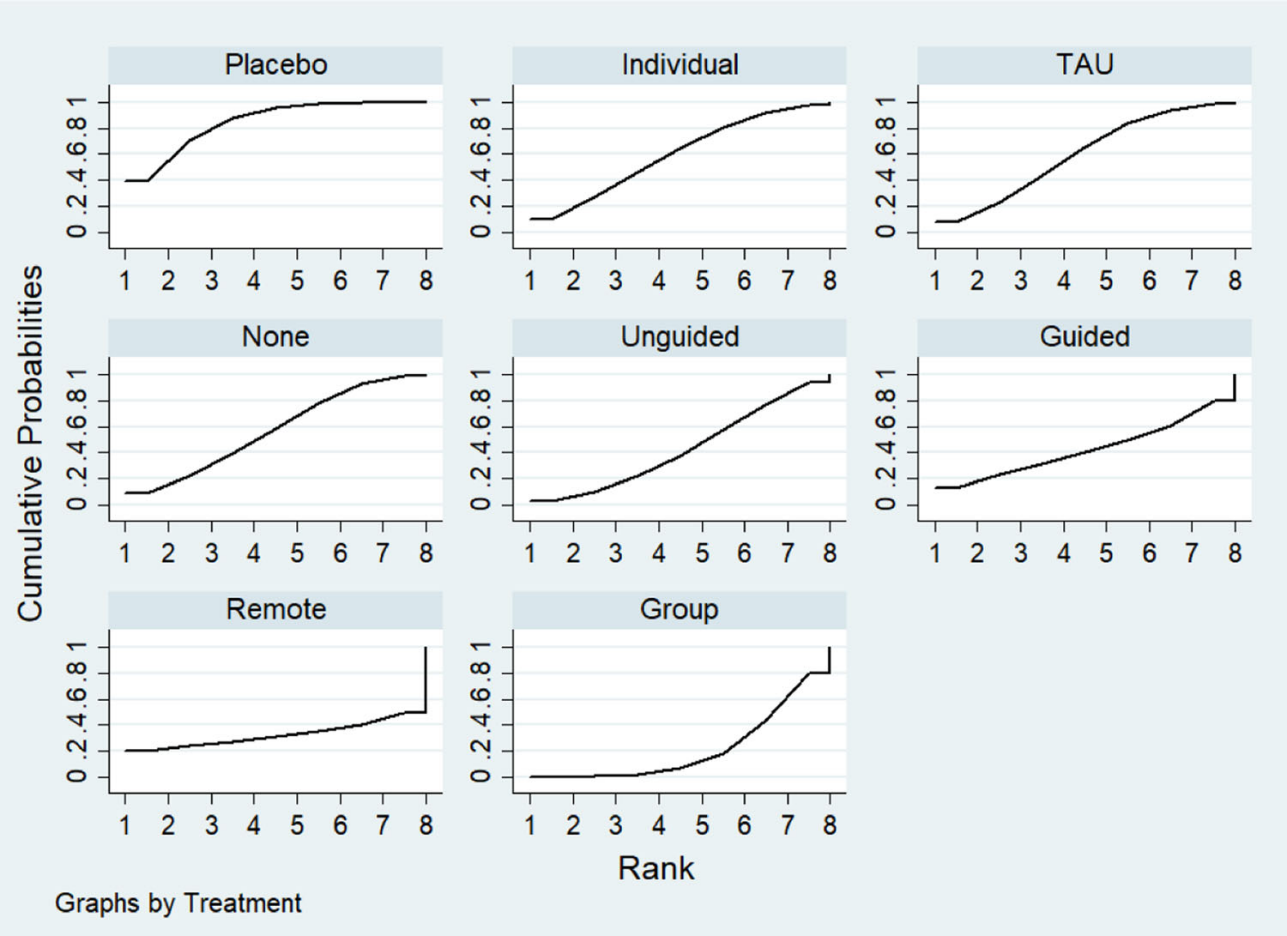
Cumulative ranking probabilities for each intervention in the intervention acceptability network. Rank indicates the cumulative probability of the intervention being the best method, the second best, the third best, and so forth.

**Figure 19:**
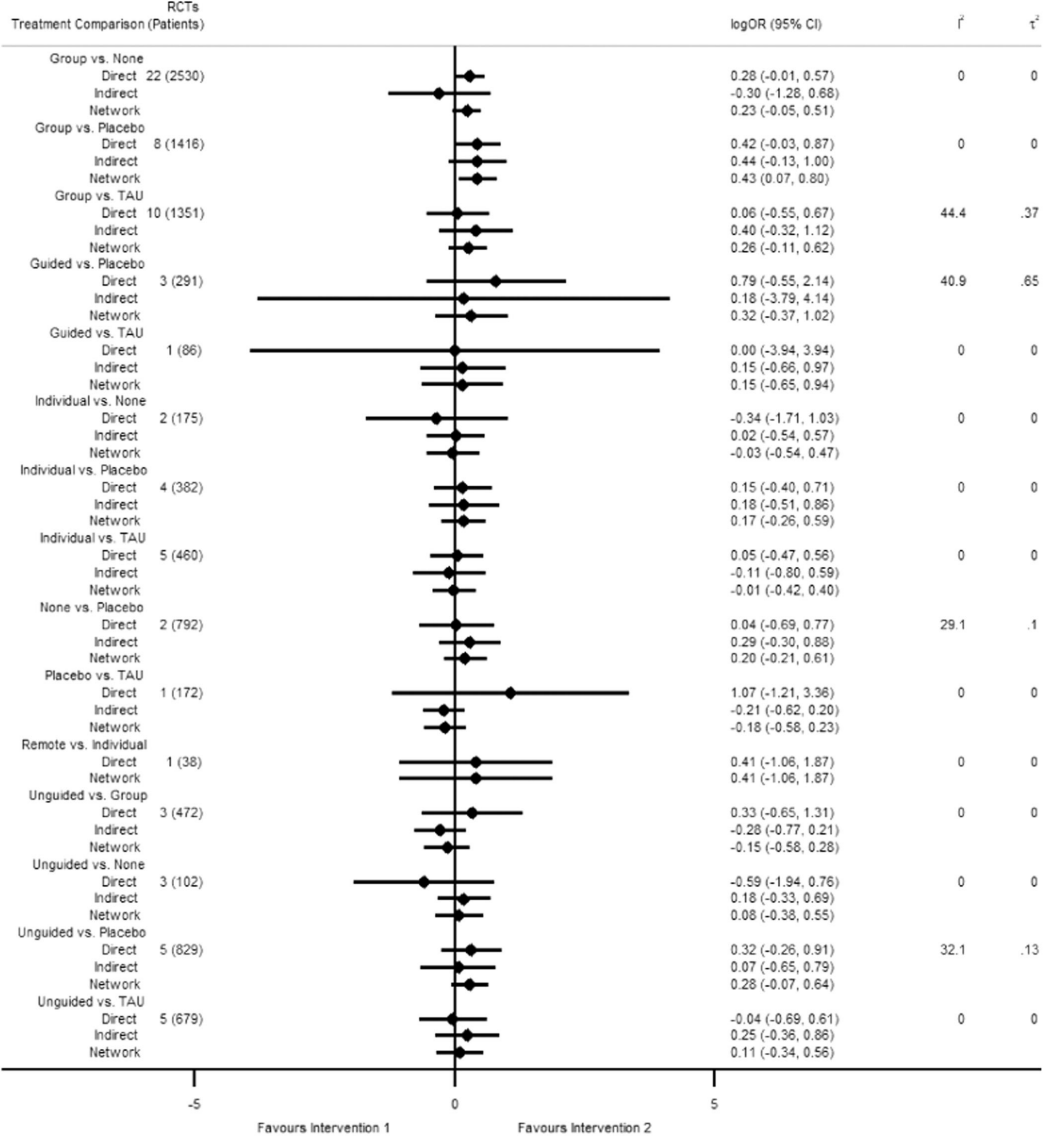
Forest plot of direct estimates of comparative effectiveness of interventions, with corresponding indirect and network estimates, for intervention acceptability. Intervention 1 is the treatment listed to the left in the treatment comparison column, and Intervention 2 is the treatment on the right. Sample size (number of randomised controlled trials and patients) and heterogeneity measures (*I* ^2^ and *τ* ^2^) are provided for the direct estimates.

Although point estimates tended to favour no intervention, no active treatments were clearly inferior, with estimates ranging from an OR of 1.45 (95% CI: 0.31 to 6.85) for remote CBT to 0.97 (95% CI: 0.58 to 1.60) for individual CBT (Figure 17, Table 6). Findings were similar with TAU or placebo as the comparator. No active treatment demonstrated superiority over another. The highest and lowest ranking active interventions were individual CBT (SUCRA 60%) and group CBT (SUCRA 21%), respectively (Figure 18, Table 6). Pairwise meta‐analytic findings were similar to those of the network meta‐analysis (Figure 19).

**Table 6:** League table for intervention acceptability.

**Placebo**	0.86 (0.49, 1.5)	2.93 (0.3, 28.74)	0.96 (0.46, 2)	0.72 (0.4, 1.3)	0.45 (0.12, 1.74)	‐	0.66 (0.42, 1.03)
0.85 (0.55, 1.29)	**Individual**	1.05 (0.63, 1.75)	0.71 (0.18, 2.79)	‐	‐	0.67 (0.15, 2.89)	‐
0.84 (0.56, 1.25)	0.99 (0.66, 1.50)	**TAU**	‐	1.04 (0.54, 1.99)	1 (0.02, 51.57)	‐	0.94 (0.51, 1.74)
0.82 (0.54, 1.23)	0.97 (0.58, 1.60)	0.98 (0.63, 1.51)	**None**	1.81 (0.47, 6.98)	‐	‐	0.75 (0.57, 1.01)
0.75 (0.53, 1.08)	0.89 (0.53, 1.48)	0.90 (0.57, 1.41)	0.92 (0.58, 1.47)	**Unguided**	‐	‐	1.39 (0.52, 3.7)
0.72 (0.36, 1.45)	0.86 (0.38, 1.93)	0.86 (0.39, 1.91)	0.89 (0.39, 2.02)	0.96 (0.43, 2.14)	**Guided**	‐	‐
0.56 (0.12, 2.60)	0.67 (0.15, 2.90)	0.67 (0.15, 3.10)	0.69 (0.15, 3.26)	0.75 (0.16, 3.55)	0.78 (0.15, 4.17)	**Remote**	‐
0.65 (0.45, 0.94)	0.77 (0.48, 1.22)	0.77 (0.54, 1.11)	0.79 (0.60, 1.05)	0.86 (0.56, 1.32)	0.89 (0.40, 2.00)	1.15 (0.25, 5.36)	**Group**
**SUCRA**							
84.8%	59.7%	59.6%	56.7%	42.9%	42.3%	32.6%	21.3%

*Note*: Odds ratios (95% confidence intervals) for each comparison in the analysis of intervention acceptability. Estimates below the diagonal represent network meta‐analysis results while those above represent pairwise meta‐analysis results. Below the diagonal, odds ratio < 1 favours the intervention in the column (above the diagonal, <1 favours the intervention in the row). Values are underlined when one intervention demonstrated superiority based on the confidence interval. Interventions are ordered based on the SUCRA values.

Abbreviations: CBT, cognitive behavioural therapy; Group, group CBT; Guided, guided self‐help; Individual, individual CBT; Remote, remote CBT; SUCRA, surface under the cumulative ranking area curve; TAU, treatment‐as‐usual; Unguided, unguided self‐help.

Based on *χ* ^2^ and *I* ^2^ statistics, heterogeneity within pairwise comparisons ranged from not important to considerable (Figure 19). At the network meta‐analytic level, the extent of heterogeneity appeared to be minimal (*τ* ^2^ ≈ 0) based on the empirical distribution for non‐pharmacological interventions with semi‐objective outcomes measured by OR (median *τ* ^2^ = 0.056, 95% range = 0.001 to 2.35) (Turner 2012). The loop‐specific (IF *p*‐values ranged from 0.486 to 0.875), node‐splitting (incoherence *p*‐values ranged from 0.255 to 0.999), and design‐by‐treatment interaction model (*p* = 0.801) approaches did not detect any incoherence.

##### Subgroup and sensitivity analyses

###### Depressive symptoms final score at post‐intervention

####### Subgroups by age category

**Figure 20:**
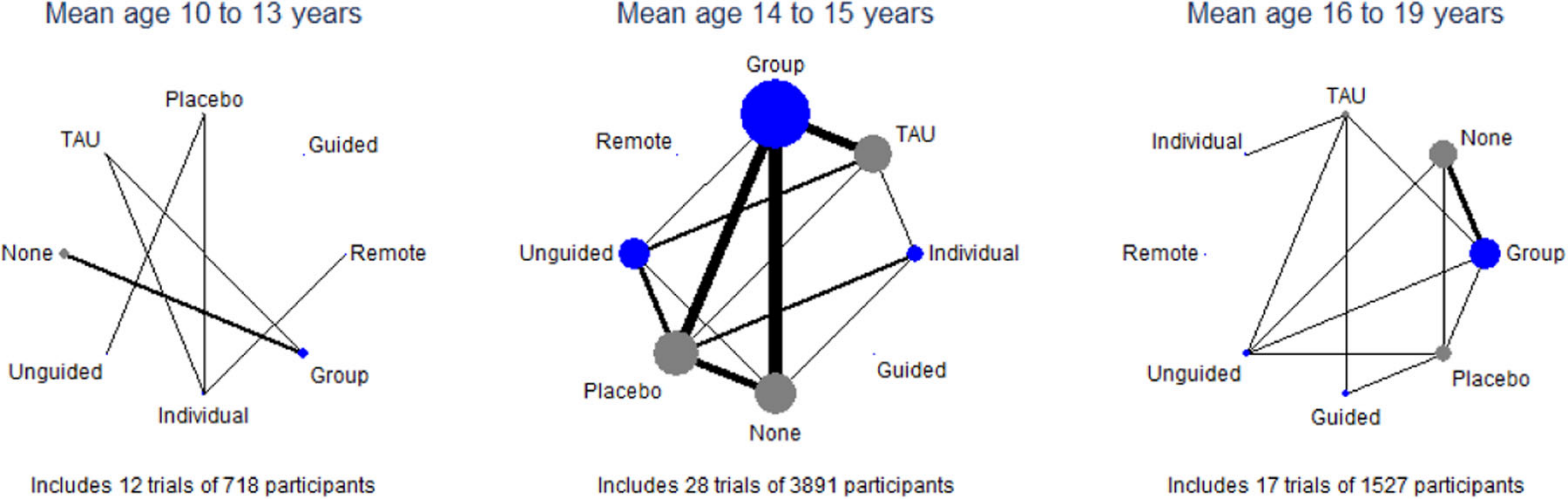
Network plot for post‐test depression score by age category subgroups. Nodes are weighted by the number of patients randomised to that intervention, and edges are weighted by the number of trials comparing the two interventions. Additionally, the subgroup networks are scaled based on the proportion of data they represent from the overall network.

Fifty‐seven of the 62 trials included in the overall analysis for post‐test depression score reported participant mean age. The mean age of participants was between 10 and 13 for 12 RCTs, between 14 and 15 for 28 RCTs, and between 16 and 19 for 17 RCTs (Figure 20). Group CBT was the most commonly studied intervention for all subgroups. Guided self‐help was only evaluated amongst RCTs focusing on participants in the oldest age category, while remote CBT was only evaluated in an RCT focusing on participants in the youngest category.

**Figure 21:**
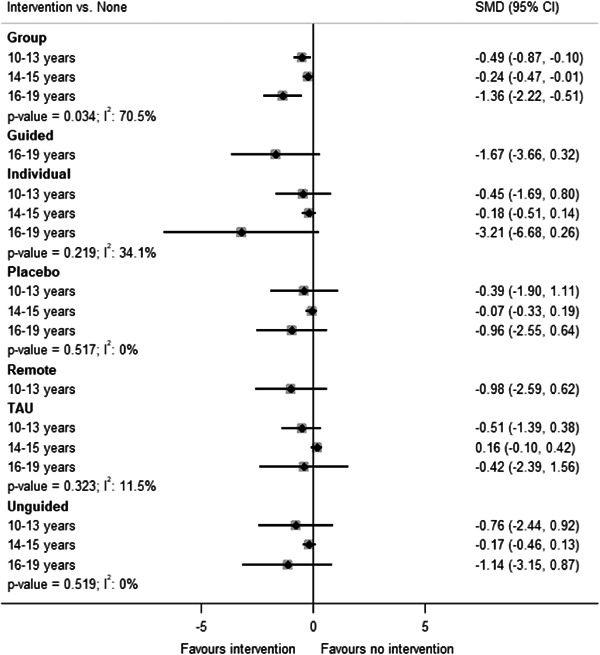
Forest plot of the network estimates for post‐test depression score for each intervention compared to no intervention by age category. *p* Values and *I* ^2^ values for subgroup differences are included.

There may be subgroup effects by age category. Using the no intervention control group as the reference, the magnitudes of effects appear to be larger for the oldest age categories compared to the other subgroups for each given comparison. However, they were also generally less precise and formal testing only indicated a significant difference for group CBT (Figure 21).

####### Sensitivity analyses

Our findings were generally robust to pre‐specified sensitivity analyses separating out the type of placebo and excluding cluster‐RCTs, as well as an additional analysis excluding studies where we had imputed SDs (Table 7). In the pre‐specified analysis excluding studies allowing for interactivity of the intervention, the effect for guided self‐help, the highest‐ranking intervention in the primary analysis, was notably attenuated (primary analysis SMD vs. no intervention: −1.29; sensitivity analysis SMD vs. no intervention: −0.77). We were not able to conduct the analyses evaluating the effects of intervention components due to lack of reported information in the studies, nor were we able to exclude studies using unvalidated measures due to all studies using validated measures.

**Table 7:** Sensitivity analysis results for post‐test depression score.

Versus None	Primary analysis (62 trials, *n* = 6436)	Separate placebo types (62 trials, *n* = 6436)	Excluding interactive interventions (52 trials, n = 5470)	Excluding cluster trials (55 trials, *n* = 4768)	Excluding imputed SDs (59 trials, *n* = 6321)
Guided	−1.29 (−2.26, −0.32)	−1.28 (−2.27, −0.29)	−0.77 (−2.68, 1.13)	−1.45 (−2.44, −0.46)	−1.40 (−2.35, −0.44)
Remote	−1.46 (−3.39, 0.48)	−1.53 (−3.50, 0.43)	−1.47 (−3.53, 0.59)	−1.65 (−3.60, 0.29)	−1.53 (−3.42, 0.37)
Group	−0.90 (−1.24, −0.56)	−0.90 (−1.25, −0.56)	−0.90 (−1.26, −0.54)	−0.95 (−1.31, −0.58)	−0.90 (−1.24, −0.55)
Individual	−0.92 (−1.60, −0.24)	−0.99 (−1.71, −0.28)	−0.93 (−1.66, −0.20)	−1.12 (−1.84, −0.40)	−0.99 (−1.66, −0.32)
Unguided	−0.71 (−1.30, −0.12)	−0.72 (−1.37, −0.07)	−0.68 (−1.54, 0.18)	−0.81 (−1.41, −0.21)	−0.79 (−1.40, −0.18)
Placebo	−0.48 (−1.00, 0.03)	‐	−0.45 (−1.03, 0.13)	−0.70 (−1.26, −0.14)	−0.62 (−1.14, −0.10)
Pill placebo	‐	−1.09 (−2.93, 0.74)	‐	‐	‐
Self‐help placebo	‐	−0.55 (−1.45, 0.35)	‐	‐	‐
Therapist‐led placebo	‐	−0.47 (−1.03, 0.09)	‐	‐	‐
TAU	−0.32 (−0.86, 0.23)	−0.33 (−0.88, 0.23)	−0.39 (−1.01, 0.23)	−0.35 (−0.95, 0.25)	−0.35 (−0.90, 0.19)

*Note*: Values represent standardised mean differences (95% confidence intervals).

Abbreviations: CBT, cognitive behavioural therapy; Group, group CBT; Guided, guided self‐help; Individual, individual CBT; Remote, remotely‐delivered CBT; SD, standard deviation; TAU, treatment‐as‐usual; Unguided, unguided self‐help.

## 7 DISCUSSION

### 7.1 Summary of main results

Sixty‐two studies were included in the network meta‐analysis for the primary outcome of depression symptoms at posttreatment, and each of the pre‐specified treatment and comparison nodes was represented as a trial arm by at least one study. At posttreatment, all active treatments (guided self‐help, group CBT, individual CBT, and unguided self‐help) except for remote CBT were found to be more effective than no treatment. However, only guided self‐help and group CBT were found to be more effective than TAU. Guided self‐help was the most highly ranked intervention, but the subgroup analysis revealed that it was only evaluated in trials with the oldest adolescents (ages 16–19 years). The results for remotely‐delivered CBT should be treated with caution as it was only evaluated in one included study with the youngest adolescents (10–13 years). The magnitude of effects also appeared to be greatest in the older age categories for each comparison, where these age groups were included in studies. However, this needs to be interpreted with caution due to the possibility of ecological bias.

The standardised effects at posttreatment are presented in terms of the difference in points on the BDI‐II between active interventions in the Summary of findings Table 1. These scores ranged from mean differences of 6.8 points to 0.2 points between intervention delivery formats. For context, the minimum clinically important difference in scores on the BDI‐II is likely to fall in the range of 3–6 points, with average treatment effects of antidepressants being 2 points (Hengartner, 2021).

Nineteen studies reported data for depressive symptom outcomes at 6‐12 month follow up. None of the included studies evaluating guided self‐help or remote CBT reported outcomes at this time point. The magnitude of effects appeared to be generally attenuated for 6‐ to 12‐month outcomes compared to post‐intervention. Although unguided self‐help was the lowest‐ranked active intervention at post‐intervention, it was the most highly ranked at 6 to 12‐month follow‐up.

In the analysis of treatment acceptability, 62 studies were included, with at least one study representing each of the pre‐specified active and control nodes in the network. We did not find clear evidence that any of the active treatments were more acceptable to participants than any others, based on attrition. This is in line with a review which found that drop‐out rates for computerised CBT for depression was similar to drop‐out rates for other psychological therapies including face‐to‐face CBT (Kaltenthaler, 2008), as well as another review which found no significant differences in acceptability between delivery formats of CBT for adults with depression (Cuijpers, 2019). In addition, a recent rapid review of the evidence found that engagement was lower and drop‐out was higher when adolescents were randomised to mental health treatment delivery formats, with drop‐out being much lower when young people could choose how to access support, indicating that randomised trials may not be the optimum design for assessing acceptability of different delivery formats (James, 2020).

### 7.2 Overall completeness and applicability of evidence

Only one study examining remote delivery of CBT for younger adolescents with depression was retrieved in the search, limiting the conclusions that may be drawn about the relative effectiveness of this particular mode of delivery and its effectiveness for older adolescents. In addition, all studies evaluating guided self‐help included older adolescents. These results may therefore only be applicable to this age group and research is needed to assess the effectiveness and acceptability of guided self‐help for younger adolescents with depression.

Very few studies included a follow‐up assessment 6 to 12 months after the end of the intervention. Although this is a common problem in trials of interventions, it is important that future studies follow young people up over longer periods to determine whether the effects of different delivery models of CBT are maintained.

Studies conducted in the United States were overrepresented (*n* = 29), potentially affecting the generalisability of the findings. However, this is not a major concern as studies from 21 different countries were included, representing a range of contexts. We were unable to find some papers (*n* = 39) or obtain the required information for others (Studies awaiting classification, *n* = 5) and thus these were excluded from the review, despite our attempts to contact authors.

We excluded studies in which the intervention was focused on treating comorbid conditions, which may be atypical for the population seen by services (Essau, 2009; Rao, 2009).

### 7.3 Quality of the evidence

Overall, our confidence in the evidence for post‐test depression symptoms was low or very low. Reasons for downgrading the evidence include a high prevalence of studies at unclear or high risk of bias in the network, the inclusion of some moderately indirect studies, imprecise effect estimates, and heterogeneity between the studies within some comparisons. Although heterogeneity appears to be moderate to large for a few comparisons, most of the comparisons have a fairly small evidence base. These cases preclude reliably estimating heterogeneity statistics as well as thoroughly investigating how sources of heterogeneity may differ between comparisons and affect transitivity. Three studies (Fischer, 1996; Rohde, 2004; Saranya, 2017) were considered to be indirect due to using incarcerated adolescent populations, three (Araki, 2019; Topooco, 2017, 2019) due to transitivity concerns with the comparison they assessed (guided self‐help vs. Placebo) only focusing on older adolescents, and one study (Nelson, 2003) due to transitivity concerns with it being the sole representative for a direct comparison (individual CBT and remote CBT).

### 7.4 Potential biases in the review process

The assignment of trial arms to network nodes has a degree of subjectivity and the authors did not always agree on node assignments. Different teams of authors therefore may have come to different decisions about node assignments for this review, which could introduce a bias.

There was some indication of publication bias, which may mean that the effects of the active treatments are inflated when compared to inactive controls. However, it is not clear that this implies that the relative effectiveness between any of the active treatments is inflated.

It was not possible to find papers for 39 studies at the full text screening stage, which could have introduced a bias, although we were not able to determine from available information whether these studies would have met the inclusion criteria and have no reason to believe that they would necessarily have had results that differed from the findings of this review. Similarly, we were unable to include the data for five included studies in the network meta‐analysis. These studies indicated no significant differences in depression outcomes for comparisons of group CBT vs unguided self‐help and for individual CBT versus TAU. These results are not inconsistent with the findings of this review for these comparisons. For three included studies, the standard deviations of outcome measures were not included in the papers, and we were unable to obtain them by contacting authors, so these were imputed. However, we have no reason to expect that this introduced a bias in the results.

The secondary outcome is limited in that loss to follow‐up is an imperfect proxy for treatment acceptability. In particular, completion of post‐test measures may not have been entirely related to engagement with treatment, particularly if incentives and other methods were used to boost response rate. Similarly, where interventions were delivered in schools, attendance to take part in the intervention and for measure completion would have been compulsory regardless of whether students found the intervention acceptable. However, this proxy facilitated quantitative comparison between studies on a consistent measure.

### 7.5 Agreements and disagreements with other studies or reviews

In their systematic review comparing delivery formats of CBT for adult depression, Cuijpers (2019) found that there was no statistically significant difference in effectiveness between individual, group, telephone, or guided self‐help formats and that all of these formats were more effective than unguided self‐help as well as waiting list and care as usual control conditions. In contrast, in this review we found that all active treatments except for remote CBT were more effective than no treatment, and that the comparisons between active treatments demonstrated neither superiority nor equivalence.

López‐López (2019) found the biggest effects for face‐to‐face CBT, with more uncertainty for the effectiveness of multimedia CBT or hybrid models of CBT, but found little evidence of differential effectiveness of face‐to‐face versus multimedia CBT interventions in a components analysis. Similarly, Bennett (2019) found significant effects for guided self‐help and unguided self‐help on symptoms of anxiety, depression, and disruptive behaviour when compared to controls, but negative effects when compared to face‐to‐face therapy, although they report that the difference in effect sizes between self‐help and face‐to‐face therapy was small and unlikely to be clinically significant. The results of this review are more nuanced, in that both face‐to‐face and hybrid models were found to be more highly ranked than unguided self‐help at posttreatment assessment time points, with no significant differences in effectiveness, and with unguided self‐help ranking higher than face‐to‐face therapy at 6‐ to 12‐month follow‐up.

A systematic review of the acceptability to patients of computerised CBT for depression in adults found that drop‐out rates were comparable to rates found for other forms of treatment in other studies (Kaltenthaler, 2008). Similarly, we found that none of the active treatment delivery models were relatively superior to any other in terms of attrition.

## 8 AUTHORS' CONCLUSIONS

### 8.1 Implications for practice

The findings of this review have implications for policy and practice and the future funding of mental health service provision by providing an understanding of how different CBT delivery modes compare to one another on a subpopulation of adolescents with elevated symptoms of depression.

Although guided self‐help had the highest ranking at post‐intervention, the included studies vary in terms of the type and amount of therapist support provided. Three of the studies examining guided self‐help involved online or app‐based interventions (Saranya, 2017; Topooco, 2017, 2019), while the fourth involved reading and worksheets for the self‐help element (Araki, 2019). Therapist support in these studies took the form of individual therapy delivered via chat (Topooco, 2017), individual face‐to‐face therapy sessions along with messages in the app (Topooco, 2019), a single group CBT session (Araki, 2019), or brief therapist feedback and reinforcement on homework each week (Saranya, 2017). The high ranking of unguided self‐help in the longer term indicates that interventions with self‐directed elements may provide young people with skills or resources that they can use to maintain the effects of the intervention, although this needs further investigation.

None of the active treatments were clearly more acceptable to participants than others, using drop‐out rates as a proxy for treatment acceptability.

The results of the network meta‐analysis may only apply to the oldest adolescents. In particular, effectiveness of guided self‐help was only found in studies of 16–19 year olds; we did not find any studies of guided self‐help with children under 16 years of age.

Finally, in clinical practice, the relative effectiveness of these intervention delivery modes must be taken into account in the context of the needs and preferences of individual young people, particularly as the differences between effect sizes were relatively small.

### 8.2 Implications for research

As guided self‐help was not directly evaluated against any of the other active treatments in this review, the results are based on indirect evidence only. Given that guided self‐help appeared to be the highest ranked intervention, at least in the short term, direct comparisons would provide further insight into not only the relative effectiveness of guided self‐help compared to other delivery formats, but also acceptability and cost‐effectiveness.

Further research into the type and amount of therapist support that is most acceptable to young people and most cost‐effective would be particularly useful. Two recent scoping reviews found that involvement of young people in research to develop preventative digital mental health interventions for children and young people, or in child health research more generally, has not been frequently reported (Bergin, 2020; Sellars, 2021). Involvement of young people in future intervention development research may increase the acceptability of different modes of delivery and may increase the chances of developing effective interventions in different formats for a wider range of ages.

Remote delivery is particularly relevant in the context of the COVID‐19 pandemic. However, this review was only able to include one study of remote delivery of CBT. Results from trials of this delivery format are needed to help commissioners and practitioners understand whether this is an effective alternative to face‐to‐face therapy.

Furthermore, trials evaluating guided self‐help and remote CBT did not collect longer‐term follow‐up data. Results over the long term are needed to determine whether effects persist over time for CBT when delivered using these formats. The high ranking of unguided self‐help at 6‐ to 12‐month follow‐up has important implications for further research into whether interventions with self‐directed elements enable young people to maintain effects by continuing or revisiting the intervention independently, and whether therapist support would improve long‐term outcomes.

Delivery modalities will differ in terms of demands on resources. This review may therefore have important cost–benefit implications, which could be examined in further research (Arnberg, 2014).

## CONTRIBUTIONS OF AUTHORS

GB and SS provided the content of the review that is pertinent to adolescent depression and interventions delivered via technology with support and guidance from NA.

GB, BR, and SS designed the methodology for the review, with suggestions and input from NA.

BR planned and performed the statistical analyses.

Screening was conducted by SS, LF, GB, and NA. Data extraction, assessment of risk of bias, and study coding for network nodes were conducted by SS, NH, and JHD, with discussion with GB.

All authors reviewed and approved the final version of the review.

## DECLARATIONS OF INTEREST

BR has recently contributed to a systematic review of interventions for major depressive disorder in adults, including cognitive behavioural therapy, to inform the development of a clinical practice guideline for the US Department of Veterans Affairs. None of the other authors have been involved in the development of any relevant interventions or primary research, and nor have they published a prior review on the topic.

## SOURCES OF SUPPORT

### 1 Internal sources

- NIHR, UK

The time of Gretchen Bjornstad and Nick Axford is supported by the National Institute for Health and Care Research Applied Research Collaboration South West Peninsula (PenARC). The views expressed in this publication are those of the authors and not necessarily those of the National Institute for Health and Care Research or the Department of Health and Social Care.

### 2 External sources

- Jacobs Foundation, Switzerland

This review was funded by the Jacobs Foundation through the Better Evidence for Children and Youth programme, grant number 2014113405. No other funding has been sought.

## DIFFERENCES BETWEEN PROTOCOL AND REVIEW

Real time remote delivery was accidentally included under face‐to‐face CBT and remote delivery in the description of treatment nodes in the protocol. This was corrected in the review.

In the protocol, we had planned to use Screen4Me to assist with screening, but did not opt to use it when screening studies for inclusion in this review.

We planned to conduct sensitivity analyses to test whether differences in intervention components confound the estimated differences between delivery modes, but were unable to do this as the descriptions of the interventions in most studies were not sufficient to identify these types of differences. We also planned to conduct a sensitivity analyses excluding studies where symptoms of depression were established using an unvalidated measure or unclear method, but all included studies measured depression at baseline using at least one validated measure.

### PEER REVIEW

The peer review history for this article is available at https://www.webofscience.com/api/gateway/wos/peer-review/10.1002/cl2.1376.

## Supporting information

Supporting information.

Supporting information.

## Data Availability

The data that support the findings of this study are available from the corresponding author upon reasonable request.
