# Supplementary material for: A comparison of the effectiveness of cognitive behavioural interventions based on delivery features for elevated symptoms of depression in adolescents: A systematic review
Source: Campbell Syst Rev. 2024 Jan 7;20(1):e1376. doi: 10.1002/cl2.1376 (PMC10771715; doi:10.1002/cl2.1376)
Supplement: Supplementary file 1 — Supporting information. [file CL2-20-e1376-s001.docx]

**Appendices**

**Appendix 1. Search Strategies**

**Cochrane Central Register of Controlled Trials (CENTRAL)**

via Wiley http://onlinelibrary.wiley.com/

Issue 11 of 12, November 2020

Searched on: 12^th^ November 2020

Records retrieved: 3150

#1 MeSH descriptor: [Depression] this term only 12232

#2 MeSH descriptor: [Depressive Disorder] this term only 7847

#3 MeSH descriptor: [Depressive Disorder, Major] this term only 4935

#4 MeSH descriptor: [Dysthymic Disorder] this term only 178

#5 (depress* or mood*):ti,kw 60386

#6 (depress* near/3 (acute or clinical* or diagnos* or disorder* or elevated or major or unipolar or illness or scale* or scor* or schedule? or adolesc* or child* or young adult* or student? or teen* or patient* or participant* or people or inpatient* or in-patient* or outpatient* or out-patient*)):ab 36989

#7 (depress* and (Beck* or BDI* or DSM* or diagnostic next schedule? or diagnostic next interview? or psychiatric next assessment? or self next report* or (Statistical Manual near/2 Mental Disorders) or Hamilton or HAM-D or HAMD or MADRS or (International Classification near/2 Disease?) or ICD-10 or ICD-9 or CESD-R or CDI or CDRS-R or CDS or HDRS or MFQ or RCDS or PHQ-9 or PHQ or PHQ-A or PHQA or K-SADS or DISC or DICA-R or CAPA)):ab 22380

#8 with next depressi*:ab 4092

#9 (dysthymic or dysthymia):ti,ab,kw 953

#10 #1 or #2 or #3 or #4 or #5 or #6 or #7 or #8 or #9 74443

#11 MeSH descriptor: [Cognitive Behavioral Therapy] this term only 8118

#12 MeSH descriptor: [Counseling] this term only 4186

#13 MeSH descriptor: [Psychotherapy, Group] this term only 2174

#14 MeSH descriptor: [Psychodrama] explode all trees 183

#15 MeSH descriptor: [Psychotherapy, Rational-Emotive] this term only 26

#16 MeSH descriptor: [Sensitivity Training Groups] this term only 25

#17 MeSH descriptor: [Bibliotherapy] this term only 126

#18 (CBT or CBGT* or bCBT or b-CBT or gCBT or g-CBT or iCBT or i-CBT or cCBT or c-CBT):ti,ab,kw 8594

#19 ((cogniti* or behavio*) near/3 (counsel* or intervention* or therap* or psychotherap* or training or treatment* or technique* or restructur* or program*)):ti,ab,kw 52947

#20 (cognitive next behavio*):ti,ab,kw 19667

#21 (rational next emoti* or (problem* near/2 (focus* or sol*)) or psychoeducat* or psycho next educat* or role next play* or schema* or self-control* or selfcontrol*):ti,ab,kw 14827

#22 ((attribution* or reattribution*) near/3 (therap* or psychotherap* or training)):ti,ab,kw 57

#23 (behavio* near/3 (activation or modification)):ti,ab,kw 2406

#24 ((thought* near/3 suppress*) or rumination):ti,ab,kw 833

#25 (((individually or group or conjoint or family) near/2 (counsel* or intervention* or program* or psychotherap* or therap* or train* or treat*)) and cognitive):ti,ab,kw 14546

#26 ((selfhelp or self-help or selfmanag* or self-manag* or selfinstruct* or self-instruct* or selfdirect* or self-direct* or selfguid* or self-guid*) and cognitive):ti,ab,kw 2469

#27 (bibliotherap* and cognitive):ti,ab,kw 152

#28 MeSH descriptor: [Psychotherapy] this term only 2570

#29 #11 or #12 or #13 or #14 or #15 or #16 or #17 or #18 or #19 or #20 or #21 or #22 or #23 or #24 or #25 or #26 or #27 or #28 76768

#30 #10 and #29 17148

#31 MeSH descriptor: [Adolescent] this term only 103483

#32 MeSH descriptor: [Child] this term only 48494

#33 MeSH descriptor: [Young Adult] this term only 63907

#34 (child* or boy* or girl* or kids or juvenil* or minors or paediatric* or pediatric* or adolesc* or preadolesc* or pre-adolesc* or pubert* or pubescen* or prepube* or pre-pube* or teen* or (young next (adult* or people or person or persons or survivor* or offender* or minorit* or people or person?)) or youth* or school? or student? or cyp):ti,ab,kw 328383

#35 #31 or #32 or #33 or #34 328383

#36 #35 and #30 5209

#37 #35 and #30 in Trials 5148

#38 #35 and #30 with Cochrane Library publication date Between Jan 2016 and Dec 2020, in Trials 3150

**MEDLINE(R) ALL**

via Ovid http://ovidsp.ovid.com/

1946 to November 10, 2020

Searched on: 12^th^ November 2020

Records retrieved: 1351

1 controlled clinical trial.pt. (93920)

2 randomized controlled trial.pt. (516819)

3 Clinical Trials as Topic/ (193622)

4 (randomi#ed or randomi#ation or randomi#ing).ti,ab,kf. (658796)

5 (RCT or "at random" or (random* adj3 (administ* or allocat* or assign* or class* or cluster or crossover or cross-over or control* or determine* or divide* or division or distribut* or expose* or fashion or number* or place* or pragmatic or quasi or recruit* or split or substitut* or treat*))).ti,ab,kf. (580926)

6 placebo.ab,ti,kf. (218476)

7 trial.ti. (228511)

8 (control* adj3 group*).ab. (551563)

9 (control* and (trial or study or group*) and (waitlist* or wait* list* or ((treatment or care) adj2 usual))).ti,ab,kf. (22634)

10 ((single or double or triple or treble) adj2 (blind* or mask* or dummy)).ti,ab,kf. (176707)

11 double-blind method/ or random allocation/ or single-blind method/ (282743)

12 or/1-11 (1765022)

13 exp animals/ not humans.sh. (4754888)

14 12 not 13 (1531346)

15 Depression/ (121562)

16 Depressive Disorder/ (73266)

17 Depressive Disorder, Major/ (30535)

18 Dysthymic Disorder/ (1141)

19 (depress* or mood*).ti,kf. (189030)

20 (depress* adj3 (acute or clinical* or diagnos* or disorder* or elevated or major or unipolar or illness or scale* or scor* or schedule? or adolesc* or child* or young adult* or student? or teen* or patient* or participant* or people or inpatient* or in-patient* or outpatient* or out-patient*)).ab. (162463)

21 (depress* and (Beck* or BDI* or DSM* or diagnostic schedule? or diagnostic interview? or psychiatric assessment? or self report* or (Statistical Manual adj2 Mental Disorders) or Hamilton or HAM-D or HAMD or MADRS or (International Classification adj2 Disease?) or ICD-10 or ICD-9 or CESD-R or CDI or CDRS-R or CDS or HDRS or MFQ or RCDS or PHQ-9 or PHQ or PHQ-A or PHQA or K-SADS or DISC or DICA-R or CAPA)).ab. (71571)

22 "with depressi*".ab. (28286)

23 (dysthymic or dysthymia).ti,ab,kf. (3149)

24 or/15-23 (344399)

25 Cognitive Behavioral Therapy/ (25849)

26 Counseling/ (36185)

27 Psychotherapy, Group/ (14014)

28 Psychotherapy, Rational-Emotive/ (194)

29 Psychotherapy/ (54388)

30 psychodrama/ or role playing/ or Sensitivity Training Groups/ (3512)

31 Bibliotherapy/ (413)

32 (CBT or CBGT* or bCBT or b-CBT or gCBT or g-CBT or iCBT or i-CBT or cCBT or c-CBT).ti,ab,kf. (12352)

33 ((cogniti* or behavio*) adj3 (counsel* or intervention* or therap* or psychotherap* or training or treatment* or technique* or restructur* or program*)).ti,ab,kf. (93064)

34 (cognitive adj behavio*).ti,ab,kf. (29226)

35 (rational emoti* or (problem* adj2 (focus* or sol*)) or psychoeducat* or psycho educat* or role play* or schema* or selfcontrol* or self control*).ti,ab,kf. (99152)

36 ((attribution* or reattribution*) adj3 (therap* or psychotherap* or training)).ti,ab,kf. (87)

37 ((behavio* adj3 (activation or modification)) or ((thought* adj3 suppress*) or rumination)).ti,ab,kf. (14059)

38 (((individually or group or conjoint or family) adj2 (counsel* or intervention* or program* or psychotherap* or therap* or train* or treat*)) and cognitive).ti,ab,kf. (10966)

39 ((selfhelp or self help or selfmanag$ or self manag$ or selfinstruct$ or self instruct$ or selfdirect$ or self direct$ or selfguid$ or self guid$) and cognitive).ti,ab,kf. (3225)

40 (bibliotherap$ and cognitive).ti,ab,kf. (150)

41 or/25-40 (310595)

42 adolescent/ or young adult/ or child/ (3231641)

43 (child* or adolesc* or paediatr* or pediatr*).hw,jn. (3228988)

44 (child* or boy* or girl* or kids or juvenil* or minors or paediatric* or pediatric* or adolesc* or preadolesc* or pre-adolesc* or pubert* or pubescen* or prepube* or pre-pube* or teen* or (young adj (adult* or people or person or persons or survivor* or offender* or minorit*)) or youth* or school? or student? or cyp).ti,ab,kf. (2403312)

45 or/42-44 (4505988)

46 14 and 24 and 41 and 45 (3597)

47 (2016* or 2017* or 2018* or 2019* or 2020*).yr,dp,dt,ep,ez. (6368876)

48 46 and 47 (1351)

**Embase**

via Ovid http://ovidsp.ovid.com/

1974 to 2020 November 10

Searched on: 12^th^ November 2020

Records retrieved: 1617

1 randomized controlled trial/ (632469)

2 randomization.de. (89092)

3 controlled clinical trial/ (466690)

4 *clinical trial/ (17627)

5 placebo.de. (359271)

6 placebo.ti,ab. (315481)

7 trial.ti. (315049)

8 (randomi#ed or randomi#ation or randomi#ing).ti,ab,kw. (953099)

9 (RCT or "at random" or (random* adj3 (administ* or allocat* or assign* or class* or cluster* or control* or determine* or divide* or division or distribut* or expose* or fashion or number* or place* or recruit* or split or substitut* or treat*))).ti,ab,kw. (775796)

10 ((singl$ or doubl$ or trebl$ or tripl$) adj3 (blind$ or mask$ or dummy)).mp. (317344)

11 (control* and (trial or study or group*) and (placebo or waitlist* or wait* list* or ((treatment or care) adj2 usual))).ti,ab,kw,hw. (367147)

12 or/1-11 (1872727)

13 depression/ (375168)

14 major depression/ (66074)

15 dysthymia/ (8511)

16 (depress* or mood*).ti,kw. (258321)

17 (depress* adj3 (acute or clinical* or diagnos* or disorder* or elevated or major or unipolar or illness or scale* or scor* or schedule? or adolesc* or child* or young adult* or student? or teen* or patient* or participant* or people or inpatient* or in-patient* or outpatient* or out-patient*)).ab. (234791)

18 (depress* and (Beck* or BDI* or DSM* or diagnostic schedule? or diagnostic interview? or psychiatric assessment? or self report* or (Statistical Manual adj2 Mental Disorders) or Hamilton or HAM-D or HAMD or MADRS or (International Classification adj2 Disease?) or ICD-10 or ICD-9 or CESD-R or CDI or CDRS-R or CDS or HDRS or MFQ or RCDS or PHQ-9 or PHQ or PHQ-A or PHQA or K-SADS or DISC or DICA-R or CAPA)).ab. (109462)

19 "with depressi*".ab. (40351)

20 (dysthymic or dysthymia).ti,ab,kw. (4167)

21 or/13-20 (574497)

22 cognitive behavioral therapy/ (13512)

23 cognitive therapy/ (43688)

24 counseling/ (68236)

25 *psychotherapy/ (39743)

26 group therapy/ (19301)

27 rational emotive behavior therapy/ (130)

28 behavior modification/ (8024)

29 psychodrama/ (985)

30 role playing/ (16586)

31 sensitivity training/ (279)

32 bibliotherapy/ (259)

33 (CBT or CBGT* or bCBT or b-CBT or gCBT or g-CBT or iCBT or i-CBT or cCBT or c-CBT).ti,ab,kw. (18725)

34 ((cogniti* or behavio*) adj3 (counsel* or intervention* or therap* or psychotherap* or training or treatment* or technique* or restructur* or program*)).ti,ab,kw. (129712)

35 (cognitive adj behavio*).ti,ab,kw. (42334)

36 (rational emoti* or (problem* adj2 (focus* or sol*)) or psychoeducat* or psycho educat* or role play* or schema* or self-control* or selfcontrol*).ti,ab,kw. (126000)

37 ((attribution* or reattribution*) adj3 (therap* or psychotherap* or training)).ti,ab,kw. (150)

38 ((behavio* adj3 (activation or modification)) or ((thought* adj3 suppress*) or rumination)).ti,ab,kw. (17791)

39 (((individually or group or conjoint or family) adj2 (counsel* or intervention* or program* or psychotherap* or therap* or train* or treat*)) and cognitive).ti,ab,kw. (16835)

40 ((selfhelp or self help or selfmanag$ or self manag$ or selfinstruct$ or self instruct$ or selfdirect$ or self direct$ or selfguid$ or self-guid$) and cognitive).ti,ab,kw. (4738)

41 (bibliotherap$ and cognitive).ti,ab,kw. (209)

42 or/22-41 (429663)

43 young adult/ (376696)

44 exp adolescent/ or juvenile/ (1574738)

45 child/ (1756427)

46 (child* or adolesc* or paediatr* or pediatr*).hw,jn. (3230912)

47 (child* or boy* or girl* or kids or juvenil* or minors or paediatric* or pediatric* or adolesc* or preadolesc* or pre-adolesc* or pubert* or pubescen* or prepube* or pre-pube* or teen* or (young adj (adult* or people or person or persons or survivor* or offender* or minorit*)) or youth* or school? or student? or cyp).ti,ab,kw. (2999057)

48 or/43-47 (4460976)

49 12 and 21 and 42 and 48 (3719)

50 adolescent depression/ (869)

51 12 and 42 and 50 (73)

52 49 or 51 (3721)

53 (2016* or 2017* or 2018* or 2019* or 2020*).yr,dp,dc. (8544765)

54 52 and 53 (1617)

**PsycINFO**

via Ovid http://ovidsp.ovid.com/

1806 to November week 1, 2020

Searched on: 12^th^ November 2020

Records retrieved: 1220

1 clinical trials.sh. (11806)

2 (randomi#ed or randomi#ation or randomi#ing).ti,ab,id. (89297)

3 (RCT or at random or (random* adj3 (administ* or allocat* or assign* or class* or cluster* or control* or crossover or cross-over or determine* or divide* or division or distribut* or expose* or fashion or number* or place* or recruit* or split or substitut* or treat*))).ti,ab,id. (107327)

4 (control* and (trial or study or group) and (placebo or waitlist* or wait* list* or ((treatment or care) adj2 usual))).ti,ab,id,hw. (29919)

5 ((single or double or triple or treble) adj2 (blind* or mask* or dummy)).ti,ab,id. (26654)

6 trial.ti. (31469)

7 placebo.ti,ab,id,hw. (40767)

8 treatment outcome.md. (20939)

9 treatment effectiveness evaluation.sh. (25027)

10 mental health program evaluation.sh. (2148)

11 or/1-10 (201874)

12 major depression/ (125673)

13 dysthymic disorder/ (1495)

14 (depress* or mood*).ti,id. (187240)

15 (depress* adj3 (acute or clinical* or diagnos* or disorder* or elevated or major or unipolar or illness or scale* or scor* or schedule? or adolesc* or child* or young adult* or student? or teen* or patient* or participant* or people or inpatient* or in-patient* or outpatient* or out-patient*)).ab. (142573)

16 (depress* and (Beck* or BDI* or DSM* or diagnostic schedule? or diagnostic interview? or psychiatric assessment? or self report* or (Statistical Manual adj2 Mental Disorders) or Hamilton or HAM-D or HAMD or MADRS or (International Classification adj2 Disease?) or ICD-10 or ICD-9 or CESD-R or CDI or CDRS-R or CDS or HDRS or MFQ or RCDS or PHQ-9 or PHQ or PHQ-A or PHQA or K-SADS or DISC or DICA-R or CAPA)).ab. (65627)

17 "with depressi*".ab. (24127)

18 (dysthymic or dysthymia).ti,ab,id. (3817)

19 or/12-18 (251991)

20 cognitive behavior therapy/ (20142)

21 cognitive therapy/ (13515)

22 cognitive techniques/ (1697)

23 cognitive restructuring/ (799)

24 exp counseling/ (77914)

25 group psychotherapy/ (19911)

26 rational emotive behavior therapy/ (1820)

27 psychotherapy/ (53450)

28 psychodrama/ (2457)

29 role playing/ (2408)

30 sensitivity training/ (1210)

31 bibliotherapy/ (750)

32 psychoeducation/ (4684)

33 schema therapy/ (322)

34 behavior modification/ (10500)

35 thought suppression/ (639)

36 "rumination (cognitive process)"/ (2431)

37 (CBT or CBGT* or bCBT or b-CBT or gCBT or g-CBT or iCBT or i-CBT or cCBT or c-CBT).ti,ab,id. (14979)

38 ((cogniti* or behavio*) adj3 (counsel* or intervention* or therap* or psychotherap* or training or treatment* or technique* or restructur* or program*)).ti,ab,id. (121157)

39 (cognitive adj behavio*).ti,ab,id. (44480)

40 (rational emoti* or (problem* adj2 (focus* or sol*)) or psychoeducat* or psycho educat* or role play* or schema* or self-control* or selfcontrol*).ti,ab,id. (119158)

41 ((attribution* or reattribution*) adj3 (therap* or psychotherap* or training)).ti,ab,id. (414)

42 ((behavio* adj3 (activation or modification)) or ((thought* adj3 suppress*) or rumination)).ti,ab,id. (16693)

43 (((individually or group or conjoint or family) adj2 (counsel* or intervention* or program* or psychotherap* or therap* or train* or treat*)) and cognitive).ti,ab,id. (13430)

44 ((selfhelp or self help or selfmanag$ or self manag$ or selfinstruct$ or self instruct$ or selfdirect$ or self direct$ or selfguid$ or self-guid$) and cognitive).ti,ab,id. (4061)

45 (bibliotherap$ and cognitive).ti,ab,id. (275)

46 or/20-45 (389369)

47 (child* or adolesc* or paediatr* or pediatr*).hw,jw. (424883)

48 (child* or boy* or girl* or kids or juvenil* or minors or paediatric* or pediatric* or adolesc* or preadolesc* or pre-adolesc* or pubert* or pubescen* or prepube* or pre-pube* or teen* or (young adj (adult* or people or person or persons or survivor* or offender* or minorit*)) or youth* or school? or student? or cyp).ti,ab,id. (1544078)

49 47 or 48 (1574629)

50 11 and 19 and 46 and 49 (1818)

51 11 and 19 and 46 (7765)

52 limit 51 to (180 school age <age 6 to 12 yrs> or 200 adolescence <age 13 to 17 yrs> or 320 young adulthood <age 18 to 29 yrs>) (2690)

53 50 or 52 (3306)

54 (2016* or 2017* or 2018* or 2019* or 2020*).yr,an,up. (910483)

55 53 and 54 (1220)

**CINAHL Complete**

via Ebsco https://www.ebsco.com/products/research-databases/cinahl-complete

Inception – 20201111

Searched on: 16^th^ November 2020

Records retrieved: 1144

**# Query Results**

S1 (MH "Randomized Controlled Trials") 110,592

S2 (MH "Double-Blind Studies") 49,271

S3 (MH "Single-Blind Studies") 14,440

S4 (MH "Random Assignment") 64,661

S5 (MH "Pretest-Posttest Design") 44,349

S6 (MH "Cluster Sample") 4,578

S7 TI randomised OR randomized 109,227

S8 AB random* 322,692

S9 TI trial 111,681

S10 MH (sample size) AND AB (assigned OR allocated OR control) 4,085

S11 MH (placebos) 12,997

S12 PT (randomized controlled trial) 124,467

S13 AB (control W5 group) 115,192

S14 MH (crossover design) OR MH (comparative studies) 366,055

S15 AB (cluster W3 RCT) 367

S16 MH animals+ 95,285

S17 MH (animal studies) 131,180

S18 TI (animal model*) 3,247

S19 S16 OR S17 OR S18 218,201

S20 MH (human) 2,279,538

S21 S19 NOT S20 189,609

S22 S1 OR S2 OR S3 OR S4 OR S5 OR S6 OR S7 OR S8 OR S9 OR S10 OR S11 OR S12 OR S13 OR S14 OR S15 806,189

S23 S22 NOT S21 767,196

S24 (MH "Control Group") 12,306

S25 TI ( group or groups ) OR AB ( group or groups ) 773,978

S26 TI assign* OR AB assign* 79,061

S27 (MH "Multicenter Studies") 265,026

S28 TI ( multicentre* or multi-centre* or multicenter* or multi-center* ) OR AB ( multicentre* or multi-centre* or multicenter* or multi-center* ) 53,782

S29 (MH "Controlled Before-After Studies") 180

S30 TI before N3 after OR AB before N3 after 80,200

S31 S24 OR S25 OR S26 OR S27 OR S28 OR S29 OR S30 1,053,515

S32 S31 not S21 1,003,259

S33 S23 OR S32 1,286,801

S34 (MH "Depression") 109,780

S35 (MH "Dysthymic Disorder") 439

S36 TI depress* or mood* 69,627

S37 AB depress* N3 (acute or clinical* or diagnos* or disorder* or elevated or major or unipolar or illness or scale* or scor* or schedule* or adolesc* or child* or "young adult" or "young adults" or student* or teen* or patient* or participant* or people or inpatient* or in-patient* or outpatient* or out-patient*) 62,728

S38 AB depress* and (Beck* or BDI* or DSM* or (diagnostic N1 schedule*) or (diagnostic N1 interview*) or (psychiatric N1 assessment*) or (self N1 report*) or (Statistical Manual N2 Mental Disorders) or Hamilton or HAM-D or HAMD or MADRS or (“International Classification” N2 Disease*) or ICD-10 or ICD-9 or CESD-R or CDI or CDRS-R or CDS or HDRS or MFQ or RCDS or PHQ-9 or PHQ or PHQ-A or PHQA or K-SADS or DISC or DICA-R or CAPA)) 26,652

S39 TI ( dysthymic or dysthymia ) OR AB ( dysthymic or dysthymia ) 807

S40 S34 OR S35 OR S36 OR S37 OR S38 OR S39 150,403

S41 (MH "Cognitive Therapy") 19,227

S42 (MH "Counseling") 30,971

S43 (MH "Psychotherapy") 21,727

S44 (MH "Psychotherapy, Group") 5,656

S45 (MH "Behavior Modification") 2,460

S46 (MH "Psychodrama+") 2,563

S47 (MH "Bibliotherapy") 403

S48 TI ( CBT or CBGT* or bCBT or b-CBT or gCBT or g-CBT or iCBT or i-CBT or cCBT or c-CBT ) OR AB ( CBT or CBGT* or bCBT or b-CBT or gCBT or g-CBT or iCBT or i-CBT or cCBT or c-CBT ) 6,057

S49 TI ( (cogniti* or behavio*) N3 (counsel* or intervention* or therap* or psychotherap* or training or treatment* or technique* or restructur* or program*) ) OR AB ( (cogniti* or behavio*) N3 (counsel* or intervention* or therap* or psychotherap* or training or treatment* or technique* or restructur* or program*) ) 51,336

S50 TI cognitive N1 behavio* OR AB cognitive N1 behavio* 18,745

S51 TI ( ((rational N1 emoti*) or (problem* N2 (focus* or sol*)) or psychoeducat* or psycho-educat* or role-play* or schema* or self-control* or selfcontrol*) ) OR AB ( ((rational N1 emoti*) or (problem* N2 (focus* or sol*)) or psychoeducat* or psycho-educat* or role-play* or schema* or self-control* or selfcontrol*) ) 34,821

S52 TI ( (attribution* or reattribution*) N3 (therap* or psychotherap* or training) ) OR AB ( (attribution* or reattribution*) N3 (therap* or psychotherap* or training) ) 55

S53 TI ( (behavio* N3 (activation or modification)) or (thought* N3 suppress*) or rumination ) OR AB ( (behavio* N3 (activation or modification)) or (thought* N3 suppress*) or rumination ) 4,684

S54 TI ( (((individually or group or conjoint or family) N2 (counsel* or intervention* or program* or psychotherap* or therap* or train* or treat*)) and cognitive) ) OR AB ( (((individually or group or conjoint or family) N2 (counsel* or intervention* or program* or psychotherap* or therap* or train* or treat*)) and cognitive) ) 7,421

S55 TI ( ((selfhelp or self N1 help or selfmanag* or self N1 manag* or selfinstruct* or self N1 instruct* or selfdirect* or self N1 direct* or selfguid* or self N1 guid*) and cognitive) ) OR AB ( ((selfhelp or self N1 help or selfmanag* or self N1 manag* or selfinstruct* or self N1 instruct* or selfdirect* or self N1 direct* or selfguid* or self N1 guid*) and cognitive) ) 1,801

S56 TI ( bibliotherap* and cognitive ) OR AB ( bibliotherap* and cognitive ) 64

S57 S41 OR S42 OR S43 OR S44 OR S45 OR S46 OR S47 OR S48 OR S49 OR S50 OR S51 OR S52 OR S53 OR S54 OR S55 OR S56 156,046

S58 (MH "Adolescence+") OR (MH "Young Adult") OR (MH "Child") OR (MH "Child, Abandoned") OR (MH "Child, Adopted") OR (MH "Child, Disabled") OR (MH "Child, Foster") OR (MH "Child, Gifted") OR (MH "Child, Hospitalized") OR (MH "Child, Institutionalized") OR (MH "Child, Medically Fragile") OR (MH "Latchkey Children") OR (MH "Only Child") 908,220

S59 MW ( child* or adolesc* or paediatr* or pediatr* ) OR JN ( child* or adolesc* or paediatr* or pediatr* ) 1,035,749

S60 TI ( child* or boy* or girl* or kids or juvenil* or minors or paediatric* or pediatric* or adolesc* or preadolesc* or pre-adolesc* or pubert* or pubescen* or prepube* or pre-pube* or teen* or (young N1 (adult* or people or person or persons or survivor* or offender* or minorit*)) or youth* or school* or student* or cyp ) OR AB ( child* or boy* or girl* or kids or juvenil* or minors or paediatric* or pediatric* or adolesc* or preadolesc* or pre-adolesc* or pubert* or pubescen* or prepube* or pre-pube* or teen* or (young N1 (adult* or people or person or persons or survivor* or offender* or minorit*)) or youth* or school* or student* or cyp ) 956,416

S61 S58 OR S59 OR S60 1,496,812

S62 S33 AND S40 AND S57 AND S61 2,727

S63 S33 AND S40 AND S57 AND S61

Limiters - Published Date: 20160101-20201231 1,128

S64 (ZD "in process") 506,686

S65 S62 AND S64 200

S66 S63 OR S65 1,144

**International HTA Database**

https://www.inahta.org/hta-database/

Searched on: 12^th^ November

Records retrieved: 35

((((bibliotherap*) AND (cognitive)) OR (((self help) OR (selfhelp) OR (self manag*) OR (selfmanag*) OR (selfinstruct*) OR (self instruct*) OR (selfdirect*) OR (self direct*) OR (selfguid*) OR (self guid*)) AND (cognitive)) OR ((cognitive) AND ((counsel*) OR (intervention*) OR (program*) OR (psychotherap*) OR (therap*) OR (train*) OR (treat*)) AND ((individually) OR (group) OR (conjoint) OR (family))) OR ((rumination)) OR ((thought* ) AND (suppress*)) OR ((behavio*) AND ( modification)) OR ((behavio*) AND (activation)) OR (((therap*) OR (psychotherap*) OR (training)) AND ((attribution* ) OR (reattribution*))) OR ((selfcontrol*) OR (self control*)) OR ((role play* ) OR (schema*)) OR ((psychoeducat* ) OR (psycho educat*)) OR ((problem*)[Keywords] AND (focus* OR sol* )[Keywords]) OR (((problem)[abs] AND (focus* OR sol*)[abs])) OR ((problem)[Title] AND (focus* OR sol*)[Title]) OR (rational emoti*) OR (cognitive behavio*) OR (((counsel* OR intervention* OR therap* OR psychotherap* OR training OR treatment* OR technique* OR restructur* OR program*)) AND (cogniti* OR behavio*)) OR ((CBT OR CBGT* OR bCBT OR b-CBT OR gCBT OR g-CBT OR iCBT OR i-CBT OR cCBT OR c-CBT)) OR ("Bibliotherapy"[mh]) OR ("Sensitivity Training Groups"[mh]) OR ("Role Playing"[mh]) OR ("Psychodrama"[mh]) OR ("Psychotherapy"[mh]) OR ("Psychotherapy, Rational-Emotive"[mh]) OR ("Psychotherapy, Group"[mh]) OR ("Counseling"[mh]) OR ("Cognitive Behavioral Therapy"[mh])) AND (((depress* ) OR (mood*) OR (dysthymi*)) OR ("Dysthymic Disorder"[mh]) OR ("Depressive Disorder"[mh]) OR ("Depressive Disorder, Major"[mh]) OR ("Depression"[mh]))) AND (((young adult* ) OR (young people) OR (young person) OR (young persons) OR (young survivor*) OR (young offender* ) OR (young minorit*)) OR ((pre adolesc*) OR (pre pube*)) OR (child* OR boy* OR girl* OR kids OR juvenil* OR minORs OR paediatric* OR pediatric* OR adolesc* OR preadolesc* OR pubert* OR pubescen* OR prepube* OR teen* OR youth* OR school* OR student* OR cyp) OR ("Child"[mh]) OR ("Young Adult"[mh]) OR ("Adolescent"[mh]))

**Proquest Dissertations & Theses A&I**

via Proquest https://about.proquest.com/products-services/dissertations/

Inception to 12^th^ November 2020

Searched on: 13^th^ November 2020

Records retrieved: 273 (192 after duplicates removed)

1. ((TI,AB,SU,IF(behavio* NEAR/3 (activation OR modification)) OR (TI,AB,SU,IF(thought* NEAR/3 suppress*) OR TI,AB,SU,IF(rumination))) AND ((TI,SU,IF(depress* OR dysthymi*) AND (TI,AB,SU,IF(child* OR boy* OR girl* OR kids OR juvenil* OR minors OR paediatric* OR pediatric* OR adolesc* OR preadolesc* OR pre-adolesc* OR pubert* OR pubescen* OR prepube* OR pre-pube* OR teen* OR youth* OR school? OR student? OR cyp) OR TI,AB,SU,IF(young NEAR/1 (adult* OR person OR persons OR people)) OR TI,AB,SU,IF(young NEAR/1 (survivor* OR offender* OR minorit*)))) AND (TI,AB,SU,IF(RCT OR random* OR trial*) OR TI,AB,SU,IF(placebo OR blind* OR mask* OR dummy)))) OR (((TI,AB,SU,IF((individually OR group OR conjoint OR family) NEAR/2 (counsel* OR intervention* OR program* OR psychotherap* OR therap* OR train* OR treat*)) AND TI,AB,SU,IF(cognitive)) OR (TI,AB,SU,IF(selfhelp OR selfmanag* OR selfinstruct* OR selfdirect* OR selfguid*) AND TI,AB,SU,IF(cognitive)) OR (TI,AB,SU,IF(self-help OR self-manag* OR self-instruct* OR self-direct* OR self-guid*) AND TI,AB,SU,IF(cognitive)) OR (TI,AB,SU,IF(bibliotherap*) AND TI,AB,SU,IF(cognitive)) OR (TI,AB,SU,IF(psychodrama OR psycho-drama) AND TI,AB,SU,IF(cognitive))) AND ((TI,SU,IF(depress* OR dysthymi*) AND (TI,AB,SU,IF(child* OR boy* OR girl* OR kids OR juvenil* OR minors OR paediatric* OR pediatric* OR adolesc* OR preadolesc* OR pre-adolesc* OR pubert* OR pubescen* OR prepube* OR pre-pube* OR teen* OR youth* OR school? OR student? OR cyp) OR TI,AB,SU,IF(young NEAR/1 (adult* OR person OR persons OR people)) OR TI,AB,SU,IF(young NEAR/1 (survivor* OR offender* OR minorit*)))) AND (TI,AB,SU,IF(RCT OR random* OR trial*) OR TI,AB,SU,IF(placebo OR blind* OR mask* OR dummy))))

**75 records retrieved**

2. TI,SU,IF(depress* OR dysthymi*) AND (TI,AB,SU,IF(problem* NEAR/2 (focus* OR sol*)) OR (TI,AB,SU,IF(rational NEAR/1 emoti*) OR TI,AB,SU,IF(problem* NEAR/2 (focus* OR sol*)) OR TI,AB,SU,IF(psychoeducat* OR psycho-educat*) OR TI,AB,SU,IF(role NEAR/1 play*) OR TI,AB,SU,IF(schema*) OR TI,AB,SU,IF(selfcontrol*) OR TI,AB,SU,IF(self NEAR/1 control*)) OR TI,AB,SU,IF(attribution* NEAR/3 (therap* OR psychotherap* OR training)) OR TI,AB,SU,IF(reattribution* NEAR/3 (therap* OR psychotherap* OR training))) AND (TI,AB,SU,IF(child* OR boy* OR girl* OR kids OR juvenil* OR minors OR paediatric* OR pediatric* OR adolesc* OR preadolesc* OR pre-adolesc* OR pubert* OR pubescen* OR prepube* OR pre-pube* OR teen* OR youth* OR school? OR student? OR cyp) OR TI,AB,SU,IF(young NEAR/1 (adult* OR person OR persons OR people)) OR TI,AB,SU,IF(young NEAR/1 (survivor* OR offender* OR minorit*))) AND (TI,AB,SU,IF(RCT OR random* OR trial*) OR TI,AB,SU,IF(placebo OR blind* OR mask* OR dummy))

**70 records retrieved**

3. TI,SU,IF(depress* OR dysthymi*) AND (TI,AB,SU,IF(cognitive NEAR/1 behavio*) OR (TI,AB,SU,IF(cogniti* OR behavio*) NEAR/2 TI,AB,SU,IF(counsel* OR intervention* OR therap* OR psychotherap* OR training OR treatment* OR technique* OR restructur* OR program*)) OR TI,AB,SU,IF(CBT OR CBGT* OR bCBT OR b-CBT OR gCBT OR g-CBT OR iCBT OR i-CBT OR cCBT OR c-CBT)) AND (TI,AB,SU,IF(child* OR boy* OR girl* OR kids OR juvenil* OR minors OR paediatric* OR pediatric* OR adolesc* OR preadolesc* OR pre-adolesc* OR pubert* OR pubescen* OR prepube* OR pre-pube* OR teen* OR youth* OR school? OR student? OR cyp) OR TI,AB,SU,IF(young NEAR/1 (adult* OR person OR persons OR people)) OR TI,AB,SU,IF(young NEAR/1 (survivor* OR offender* OR minorit*))) AND (TI,AB,SU,IF(RCT OR random* OR trial*) OR TI,AB,SU,IF(placebo OR blind* OR mask* OR dummy))

**128 records retrieved**

**Open Grey**

http://www.opengrey.eu/

Searched on: 20^th^ November 2020

Records retrieved: 45 (28 after duplicated removed)

1. (child* OR boy* OR girl* OR kids OR juvenil* OR minors OR paediatric* OR pediatric* OR adolesc* OR preadolesc* OR pre-adolesc* OR pubert* OR pubescen* OR prepube* OR pre-pube* OR teen* OR youth* OR school* OR student* OR cyp OR young adult* OR young people OR young person* OR young survivor* OR young offender* OR young minorit*) AND (depress* OR mood* OR dysthymi*) AND ((cogniti* OR behavio*) NEAR/3 (counsel* OR intervention* OR therap* OR psychotherap* OR training OR treatment* OR technique* OR restructur* OR program*))

**23 records retrieved**

2. (child* OR boy* OR girl* OR kids OR juvenil* OR minors OR paediatric* OR pediatric* OR adolesc* OR preadolesc* OR pre-adolesc* OR pubert* OR pubescen* OR prepube* OR pre-pube* OR teen* OR youth* OR school* OR student* OR cyp OR young adult* OR young people OR young person* OR young survivor* OR young offender* OR young minorit*) AND (depress* OR mood* OR dysthymi*) AND (CBT OR CBGT* OR bCBT OR b-CBT OR gCBT OR g-CBT OR iCBT OR i-CBT OR cCBT OR c-CBT)

**5 records retrieved**

3. (child* OR boy* OR girl* OR kids OR juvenil* OR minors OR paediatric* OR pediatric* OR adolesc* OR preadolesc* OR pre-adolesc* OR pubert* OR pubescen* OR prepube* OR pre-pube* OR teen* OR youth* OR school* OR student* OR cyp OR young adult* OR young people OR young person* OR young survivor* OR young offender* OR young minorit*) AND (depress* OR mood* OR dysthymi*) AND (cogniti* NEAR/1 behavio*)

**17 records retrieved**

**Google**

https://www.google.co.uk/advanced_search

Searched on: 30^th^ November 2020

Search string:

cognitive behavior OR cognitive behaviour

AND

depression OR depressive

AND

child OR children OR juvenile OR adolescent OR adolescence OR teen OR teenager OR teenage OR youth OR school OR student OR young adult OR young people OR young person

AND

RCT OR trial

Restricted to pdf files.

1,290,000 search results were returned by Google and the first 10 pages were copied and pasted to a word document for the research team to scan for relevance.

**Appendix 2. References for Handsearched Reviews**

1. Bevan Jones R, Thapar A, Stone Z, Thapar A, Jones I, Smith D, et al. Psychoeducational interventions in adolescent depression: A systematic review. Vol. 101, Patient Education and Counseling. Elsevier Ireland Ltd; 2018. p. 804–16.

2. Bostic JQ, Rubin DH, Prince J, Schlozman S. Treatment of depression in children and adolescents [Internet]. Vol. 11, Journal of Psychiatric Practice. 2005. p. 141–54. Available from: https://search.proquest.com/openview/4e310e93ae79dabb19e491327f97ca26/1?pq-origsite=gscholar&cbl=40635

3. Caldwell DM, Davies SR, Hetrick SE, Palmer JC, Caro P, López-López JA, et al. School-based interventions to prevent anxiety and depression in children and young people: a systematic review and network meta-analysis. The Lancet Psychiatry [Internet]. 2019;6(12):1011–20. Available from: https://www.sciencedirect.com/science/article/pii/S2215036619304031

4. Calear AL, Christensen H. Systematic review of school-based prevention and early intervention programs for depression. J Adolesc. 2010;33(3):429–38.

5. Carlbring P, Andersson G, Cuijpers P, Riper H, Hedman-Lagerlöf E. Internet-based vs. face-to-face cognitive behavior therapy for psychiatric and somatic disorders: an updated systematic review and meta-analysis. Cogn Behav Ther [Internet]. 2018;47(1):1–18. Available from: https://www.tandfonline.com/doi/full/10.1080/16506073.2017.1401115

6. Conley CS, Shapiro JB, Kirsch AC, Durlak JA. A meta-analysis of indicated mental health prevention programs for at-risk higher education students. J Couns Psychol. 2017;64(2):121–40.

7. Cuijpers P, Karyotaki E, Eckshtain D, Ng MY, Corteselli KA, Noma H, et al. Psychotherapy for Depression across Different Age Groups: A Systematic Review and Meta-analysis. JAMA Psychiatry [Internet]. 2020;77(7):694–702. Available from: https://jamanetwork.com/

8. Davaasambuu S, Hauwadhanasuk T, Matsuo H, Szatmari P. Effects of interventions to reduce adolescent depression in low- and middle-income countries: A systematic review and meta-analysis. Vol. 123, Journal of Psychiatric Research. Elsevier Ltd; 2020. p. 201–15.

9. Ebert DD, Zarski AC, Christensen H, Stikkelbroek Y, Cuijpers P, Berking M, et al. Internet and computer-based cognitive behavioral therapy for anxiety and depression in youth: A meta-analysis of randomized controlled outcome trials. Wallander JL, editor. PLoS One [Internet]. 2015;10(3):e0119895–e0119895. Available from: https://dx.plos.org/10.1371/journal.pone.0119895

10. Fu Z, Zhou S, Burger H, Bockting CLH, Williams AD. Psychological interventions for depression in Chinese university students: A systematic review and meta-analysis. J Affect Disord [Internet]. 2020;262:440–50. Available from: https://www.sciencedirect.com/science/article/pii/S0165032719312169

11. Garrido S, Millington C, Cheers D, Boydell K, Schubert E, Meade T, et al. What Works and What Doesn’t Work? A Systematic Review of Digital Mental Health Interventions for Depression and Anxiety in Young People. Vol. 10, Frontiers in Psychiatry. Frontiers Media S.A.; 2019.

12. Gee B, Reynolds S, Carroll B, Orchard F, Clarke T, Martin D, et al. Practitioner Review: Effectiveness of indicated school-based interventions for adolescent depression and anxiety – a meta-analytic review. J Child Psychol Psychiatry Allied Discip [Internet]. 2020;61(7):739–56. Available from: https://onlinelibrary.wiley.com/doi/abs/10.1111/jcpp.13209

13. Grist R, Croker A, Denne M, Stallard P. Technology Delivered Interventions for Depression and Anxiety in Children and Adolescents: A Systematic Review and Meta-analysis. Vol. 22, Clinical Child and Family Psychology Review. Springer New York LLC; 2019. p. 147–71.

14. Gualano MR, Bert F, Martorana M, Voglino G, Andriolo V, Thomas R, et al. The long-term effects of bibliotherapy in depression treatment: Systematic review of randomized clinical trials. Vol. 58, Clinical Psychology Review. Elsevier Inc.; 2017. p. 49–58.

15. Harrer M, Adam SH, Baumeister H, Cuijpers P, Karyotaki E, Auerbach RP, et al. Internet interventions for mental health in university students: A systematic review and meta-analysis. Int J Methods Psychiatr Res [Internet]. 2019;28(2). Available from: https://doi.org/10.1002/mpr.1759

16. Hetrick SE, Cox GR, Witt KG, Bir JJ, Merry SN. Cognitive behavioural therapy (CBT), third-wave CBT and interpersonal therapy (IPT) based interventions for preventing depression in children and adolescents [Internet]. Vol. 2016, Cochrane Database of Systematic Reviews. John Wiley and Sons Ltd; 2016. Available from: https://www.cochranelibrary.com/cdsr/doi/10.1002/14651858.CD003380.pub4/full

17. Higinbotham MK, Emmert-Aronson B, Bunge EL. A Meta-analysis of the Effectiveness of Behavioral Intervention Technologies and Face-to-Face Cognitive Behavioral Therapy for Youth with Depression. Vol. 5, Journal of Technology in Behavioral Science. 2020.

18. Høifødt RS, Strøm C, Kolstrup N, Eisemann M, Waterloo K. Effectiveness of cognitive behavioural therapy in primary health care: A review. Fam Pract [Internet]. 2011;28(5):489–504. Available from: https://academic.oup.com/fampra/article-lookup/doi/10.1093/fampra/cmr017

19. Hollis C, Falconer CJ, Martin JL, Whittington C, Stockton S, Glazebrook C, et al. Annual Research Review: Digital health interventions for children and young people with mental health problems – a systematic and meta-review. J Child Psychol Psychiatry Allied Discip [Internet]. 2017;58(4):474–503. Available from: http://doi.wiley.com/10.1111/jcpp.12663

20. Huang J, Nigatu YT, Smail-Crevier R, Zhang X, Wang J. Interventions for common mental health problems among university and college students: A systematic review and meta-analysis of randomized controlled trials. Vol. 107, Journal of Psychiatric Research. Elsevier Ltd; 2018. p. 1–10.

21. J K, S O, J C, H T, A G, A H, et al. Inequalities and the mental health of young people: a systematic review of secondary school-based cognitive behavioural interventions (Structured abstract). Database Abstr Rev Eff [Internet]. 2009;(2):1. Available from: http://onlinelibrary.wiley.com/o/cochrane/cldare/articles/DARE-12009107534/frame.html

22. Keles S, Idsoe T. A meta-analysis of group Cognitive Behavioral Therapy (CBT) interventions for adolescents with depression. Vol. 67, Journal of Adolescence. Academic Press; 2018. p. 129–39.

23. Lau HM, Smit JH, Fleming TM, Riper H. Serious games for mental health: Are they accessible, feasible, and effective? A systematic review and meta-analysis [Internet]. Vol. 7, Frontiers in Psychiatry. Frontiers Media S.A.; 2017. p. 1. Available from: http://www.frontiersin.org

24. Leichsenring F, Luyten P, Abbass A, Rabung S, Steinert C. Treatment of depression in children and adolescents [Internet]. Vol. 8, The Lancet Psychiatry. 2021. Available from: https://effectivehealthcare.ahrq.gov/products/childhood-depression/research

25. McLaughlin, Colleen, Holliday, Carol, Clarke, Barbie and Ilie S. Research on counselling and psychotherapy with children and young people: asystematic scoping review of the evidence for its effectiveness from 2003-2011 [Internet]. 2013. Available from: https://www.bacp.co.uk/media/1978/bacp-research-on-counselling-psychotherapy-with-children-young-people-systematic-review-2013.pdf

26. Munthe-Kaas H, Johansen S, Blaasvær N, Hammerstrøm K, Nilsen W. The Effect of Psychosocial Interventions for Preventing and Treating Depression and Anxiety Among At-Risk Children and Adolescents [Internet]. The Effect of Psychosocial Interventions for Preventing and Treating Depression and Anxiety Among At-Risk Children and Adolescents. 2014. Available from: http://www.ncbi.nlm.nih.gov/pubmed/29320103

27. Nardi B, Massei M, Arimatea E, Moltedo-Perfetti A. Effectiveness of group CBT in treating adolescents with depression symptoms: A critical review [Internet]. Vol. 29, International Journal of Adolescent Medicine and Health. Walter de Gruyter GmbH; 2017. Available from: https://www.degruyter.com/document/doi/10.1515/ijamh-2015-0080/html

28. Pollok J, van Agteren J, Chong A, Carson-Chahhoud K, Smith B. Evaluation of existing experimental evidence for treatment of depression in indigenous populations: A systematic review. Aust J Psychol [Internet]. 2018;70(4):305–17. Available from: http://doi.wiley.com/10.1111/ajpy.12204

29. Rith-Najarian LR, Boustani MM, Chorpita BF. A systematic review of prevention programs targeting depression, anxiety, and stress in university students. Vol. 257, Journal of Affective Disorders. Elsevier B.V.; 2019. p. 568–84.

30. Senanayake B, Wickramasinghe SI, Chatfield MD, Hansen J, Edirippulige S, Smith AC. Effectiveness of text messaging interventions for the management of depression: A systematic review and meta-analysis. J Telemed Telecare [Internet]. 2019;25(9):513–23. Available from: https://www.researchgate.net/publication/336699820

31. Ssegonja R, Nystrand C, Feldman I, Sarkadi A, Langenskiöld S, Jonsson U. Indicated preventive interventions for depression in children and adolescents: A meta-analysis and meta-regression. Vol. 118, Preventive Medicine. Academic Press Inc.; 2019. p. 7–15.

32. Stasiak K, Fleming T, Lucassen MFG, Shepherd MJ, Whittaker R, Merry SN. Computer-Based and Online Therapy for Depression and Anxiety in Children and Adolescents. J Child Adolesc Psychopharmacol [Internet]. 2016;26(3):235–45. Available from: http://dx.doi.org/doi:10.1089/cap.2015.0029

33. Turrini G, Purgato M, Acarturk C, Anttila M, Au T, Ballette F, et al. Efficacy and acceptability of psychosocial interventions in asylum seekers and refugees: Systematic review and meta-analysis [Internet]. Vol. 28, Epidemiology and Psychiatric Sciences. Cambridge University Press; 2019. p. 376–88. Available from: https://www.cambridge.org/core/terms.https://doi.org/10.1017/S2045796019000027Downloadedfromhttps://www.cambridge.org/core

34. Välimäki M, Anttila K, Anttila M, Lahti M. Web-based interventions supporting adolescents and young people with depressive symptoms: Systematic review and meta-analysis. JMIR mHealth uHealth [Internet]. 2017;5(12). Available from: https://mhealth.jmir.org/2017/12/e180

35. Wu J. How effective are school setting-based cognitive behavioural therapy based programmes delivered in a group format for children between the ages of 7-13 at reducing anxiety symptoms? [Internet]. Available from: https://www.ucl.ac.uk/educational-psychology/resources/CS1Wu17-20.pdf

36. Yang L, Zhou X, Zhou C, Zhang Y, Pu J, Liu L, et al. Efficacy and Acceptability of Cognitive Behavioral Therapy for Depression in Children: A Systematic Review and Meta-analysis. Vol. 17, Academic Pediatrics. Elsevier Inc.; 2017. p. 9–16.

37. Yuan S, Zhou X, Zhang Y, Zhang H, Pu J, Yang L, et al. Comparative efficacy and acceptability of bibliotherapy for depression and anxiety disorders in children and adolescents: A meta-analysis of randomized clinical trials [Internet]. Vol. 14, Neuropsychiatric Disease and Treatment. 2018. p. 353–65. Available from: https://psycnet.apa.org/record/2018-04822-001

**Appendix 3. Methodological expectations of Campbell Collaboration intervention reviews (MECCIR) Reporting Standards Table**

| **Item No.** | **Status** | **Item Name** | **Standard** | **Rationale and elaboration** | **Authors note: pages where item is addressed** |
| --- | --- | --- | --- | --- | --- |
| Title and authors | | | | |  |
| R1 | Highly desirable | Format of title | Follow the standard template for a Campbell review title. |  | 1 |
| R2 | Mandatory | Authors | List names and affiliations of all authors |  | 1-2 |
| Abstract | | | | |  |
| R3 | Mandatory | Writing the abstract | Prepare a structured abstract to provide a succinct summary of the review. In the interests of brevity it is highly desirable for authors to provide an abstract of less than 700 words, and it should be no more than 1000 words in length. | Abstracts are a prominent, publically accessible summary of the review. They should convey key information about the review question and its findings, and be informative to readers. [PRISMA item 2] | 2-3 |
| R4 | Mandatory | Abstract, Background | Summarize the rationale and context of the review. |  | 2 |
| R5 | Mandatory | Abstract, Objectives | State the main objective(s), preferably in a single concise sentence. | The objective(s) should be expressed in terms that relate to the population(s), intervention comparison(s) and, where appropriate, outcomes of interest. | 2 |
| R6 | Mandatory | Abstract, Search methods | Provide the date of the last search from which records were evaluated and any studies identified were incorporated into the review, and an indication of the databases and other sources searched. | Abstracts should aim to give readers brief but key information about the comprehensiveness of the search and the currency of the information summarized by the review.  The abstract must include the month and year of the set of searches up to which the conclusions of the review are valid. This date should reflect the date of the most recent set of searches from which all records have been screened for relevance and any studies meeting the eligibility criteria have been fully incorporated into the review (studies may be awaiting classification if, for example, the review authors are awaiting translation or clarification from authors or sponsors).  The amount of information regarding the search should be indicative of the process rather than provide specific details. In the interests of brevity certain details regarding the overall process may need to be moved to the full text of the review.  Example: “MEDLINE, PsycINFO, five other databases and three trials registers were searched on [date] together with reference checking, citation searching and contact with study authors to identify additional studies.” | 2 |
| R7 | Mandatory | Abstract, Selection criteria | Summarize eligibility criteria of the review, including information on study design, population, and comparison. | Any extensions to eligibility criteria to address adverse effects, economic issues, or qualitative research should be mentioned. | 2 |
| R8 | Mandatory | Abstract, Data collection and analysis | Summarize any noteworthy methods for selecting studies, collecting data, evaluating risk of bias/study quality, and synthesizing findings. For many reviews, it may be sufficient to state “We used standard methodological procedures expected by The Campbell Collaboration.” | This section of the abstract should indicate the rigor of the methods that underpin the results reported subsequently in the abstract. It does not need to replicate detailed description of the methods in the main text of the review.  Details of how many people were involved in the screening process and collection of information about any included studies are not necessary in the abstract. Key statistical methods may be given if not clear from the results that follow.  The abstract should prioritize the disclosure of non-standard approaches. For example, rather than disclosing all domains applied in the assessment of risk of bias/study quality, notable variations on the standard approach should be given, such as non-standard tools that were used. | 2 |
| R9 | Mandatory | Abstract, Main results: number of studies and participants | Report the number of included studies and participants. | The total number of included studies should be stated. It might be appropriate to provide numbers of studies and participants for specific comparisons and main outcomes if the amount of evidence differs substantially from the total. Numbers of participants *analyzed* should generally be presented in preference to numbers *recruited* (e.g., randomized); more important is to be clear which numbers are being reported. For some types of data there may be preferable alternatives to the number of participants (e.g., person-years of follow-up). | 3 |
| R10 | Highly desirable | Abstract, Main results: study characteristics | Provide a brief description of key characteristics that will determine the applicability of the body of evidence (e.g., age, severity of condition, setting, study duration). | Summarizing the study characteristics will provide readers of the abstract with important information about the applicability of the included studies. This is particularly important if the included studies reflect a subgroup of those eligible for inclusion in the review, for example, if the review intended to address the effects of interventions across all age groups, but included studies that only recruited adolescents. | 3 |
| R11 | Mandatory | Abstract, Main results: bias/quality assessment | Provide a comment on the findings of the risk of bias/quality assessments. | The risk of bias/study quality assessments are a key finding and form a fundamental part of the strength of the conclusions drawn in the review. If risks of bias/study quality differ substantially for different comparisons and outcomes, this may need to be mentioned. | 3 |
| R12 | Mandatory | Abstract, Main results: findings | Report findings for all primary outcomes, irrespective of the strength and direction of the result, and of the availability of data. | Findings should typically include concise information about the quality of the body of evidence for the outcome (such as study limitations, consistency of effect, imprecision, indirectness and publication bias).  Outcomes should not be selected solely on the basis of the findings. If no studies measured the primary outcomes, then a comment should be made to that effect. | 3 |
| R13 | Highly desirable | Abstract, Main results: adverse effects | Ensure that any findings related to adverse effects are reported. If adverse effects data were sought, but availability of data was limited, this should be reported. | The abstract of the review should aim to reflect a balanced summary of the benefits and harms of the intervention. | N/A |
| R14 | Mandatory | Abstract, Main results: format of numerical results | Present summaries of statistical analyses in the same way as they are reported in the review and in a standard way, ensuring that readers will understand the direction of benefit and the measurement scale used, and that confidence intervals are included where appropriate. | The standard format for reporting the results of statistical analysis includes an indication of the summary measure, point estimate and confidence interval (e.g., odds ratio 0.75, 95% CI [0.62 to 0.89]). | 3 |
| R15 | Highly desirable | Abstract, Main results: interpretability of findings | Ensure that key findings are interpretable, or are re-expressed in an interpretable way. For instance, they might be re-expressed in absolute terms (e.g., assumed and corresponding risks, NNTs, group means), and outcomes combined with a standardized scale (e.g., SMD) might be re-expressed in units that are more naturally understood. | Absolute effects provide a useful illustration of the likely impact of intervention, and are usually easier to understand than relative effects. Units expressed on a standardized scale reflect the effect estimate as the number of standard deviations. This is not intuitive to many readers who may be more familiar with specific scales. Any re-expressed findings must have been presented in the same way in the main text of the review (see previous standard). | 3 |
| R16 | Mandatory | Abstract, Implications for policy, practice, and research | State key conclusions drawn. | Authors’ conclusions may include both implications for practice and implications for research. Care must be taken to avoid interpreting lack of evidence of effect as evidence of lack of effect. *Recommendations* for practice should be avoided | 3 |
| R17 | Mandatory | Completeness of main review text | Ensure that all findings reported in the abstract, including re-expressions of meta-analysis results, also appear in the main text of the review. |  | 3 |
| R18 | Mandatory | Consistency of summary versions of the review | Ensure that reporting of objectives, important outcomes, results, caveats and conclusions is consistent across the text, the abstract and the ‘Summary of findings’ table (if included). | Summary versions of the review should be written on the assumption that they are likely to be read in isolation from the rest of the review. | 2-5 |
| Background | | | | |  |
| R19 | Mandatory | Background | Provide a concise description of the condition or problem addressed by the review question, definition of the intervention and how it might work, and why it is important to do the review. | Systematic reviews should have a clearly defined and well-reasoned rationale that has been developed in the context of existing knowledge. Outlining the context of the review question is useful to readers and helps to establish key uncertainties that the review intends to address.  [PRISMA item 3] | 5-7 |
| R20 | Highly desirable | Background headings | Include the four standard headings when writing the Background. | Four standard headings are recommended (‘Description of the problem or condition’, ‘Description of the intervention’, ‘How the intervention might work’, and ‘Why it is important to do this review’). Different headings should only be used when these standard headings are not appropriate for the content matter of the review. | 5-7 |
| R21 | Mandatory | Background references | Support all key supporting statements with references. | Claims or statements regarding aspects such as prevalence and mechanisms of action should be substantiated and, where available, supported by external evidence. | 5-7, 91-97 |
| R22 | Mandatory | Background text | Do not use plagiarized text. | Unacknowledged copying from the work of other people is not acceptable. There may however be situations in which the same text appears in different reviews, for example when the reviews are prepared by the same team.  Content that is identical to, drawn or copied from standard texts may be acceptable but must be referenced. Ensure any verbatim quotations of more than a few words are shown in quotation marks and clearly acknowledge (i.e., cite) all sources. | 5-7 |
| R23 | Mandatory | Main objective | State the main objective, where appropriate in a single concise sentence. | The primary objective of a Campbell review should be to assess the effects of one or more interventions on stakeholder-important outcomes, both intended and unintended. The objective should be expressed in terms that relate to the population(s), intervention, comparison(s) and, where appropriate to specify explicitly, the outcomes of interest. Stakeholders may be professionals, service providers, policy makers, practitioners, or others.  *MECCIR C2* (Define in advance the objectives of the review, including participants, interventions, comparators, and outcomes.)  Where possible, the format should be of the form “To assess the effects of *[intervention or comparison]* for *[topical issue]* for/in *[types of people, issue or problem and setting if specified]*”.  [PRISMA item 4] | 7 |
| R24 | Mandatory, if applicable | Secondary objectives | *If the review includes secondary objectives*, state explicitly (as secondary objectives) any specific questions being addressed by the review, such as those relating to particular participant groups, intervention comparisons, or outcomes. | The objectives should be expressed in terms that relate to the population(s), intervention comparison(s) and, where appropriate, outcomes of interest.  *MECCIR C4* (Consider in advance whether issues of equity and relevance of evidence to specific populations are important to the review, and plan for appropriate methods to address them if they are. Attention should be paid to the relevance of the review question to populations such as low socioeconomic groups, low or middle-income regions, women, people with disabilities, children, and older people.) | N/A |
| R25 | Mandatory | Economic evidence | *If economic evidence is being reviewed*, state this explicitly in the Objectives (as secondary objectives). | The primary aim of a Campbell review should be to assess the effects of one or more interventions on stakeholder-important outcomes, both intended and unintended. These outcomes may include economic outcomes, such as the impact of interventions on use of resources and/or costs. If economic evidence is being reviewed as an integrated economics component, this should be stated as a secondary objective. | N/A |
| R26 | Mandatory, if applicable | Qualitative research evidence | *If qualitative research evidence is being reviewed*, state this explicitly in the Objectives (as secondary objectives). | The primary aim of a Campbell review should be to assess the effects of one or more interventions on stakeholder-important outcomes, both intended and unintended. If qualitative research evidence is being included to ‘complement the review, this should be stated as a secondary objective. | N/A |
| Methods | | | | |  |
| R27 | Mandatory | Reference protocol | Cite the protocol for the review. | The reader should be made aware that the review is based on a published protocol. This is particularly important if the review has been split into multiple reviews since the protocol was published. The protocol should be cited using the last publication citation for the protocol. Archived versions of protocols can be accessed via the current version of the review in the Campbell Systematic Reviews.  [PRISMA item 5] | 7 |
| Criteria for inclusion and exclusion of studies in the review | | | | |  |
| R28 | Mandatory | Eligibility criteria for types of study: study designs | State eligible study designs, and provide a justification for the choice. | It is not necessary to explain why randomized trials are eligible (if that is the case), although it may be important to explain the eligibility or non-eligibility of other types of study designs.  *MECCIR C9* (Define in advance the eligibility criteria for study designs in a clear and unambiguous way, with a focus on features of a study's design rather than design labels.)  *MECCIR C11* (Justify the choice of eligible study designs.)  [PRISMA item 6] | 7-8 |
| R29 | Mandatory | Eligibility criteria for types of study: study reports | *If studies are excluded on the basis of language of publication*, explain and justify this. | Studies should be included irrespective of their publication status, and electronic availability. If studies are excluded based on their language of publication, explicit justification for this exclusion should be provided.  *MECCIR C12* (Include studies irrespective of their publication status, unless explicitly justified.)  [PRISMA item 6] | 8 |
| R30 | Mandatory | Eligibility criteria for types of participants | State eligibility criteria for participants, including any criteria around location, setting, status, or definition of condition and demographic factors, and how studies including subsets of relevant participants are handled. | Any notable restrictions on the eligibility criteria of the review should be given and explained (e.g., exclusion of people under or over a certain age, specific settings of intervention).  *MECCIR C5* (Define in advance the eligibility criteria for participants in the studies.)  *MECCIR C6* (Define in advance how studies that include only a subset of relevant participants will be handled.)  [PRISMA item 6] | 8 |
| R31 | Mandatory | Eligibility criteria for types of interventions | State eligibility criteria for interventions and comparators, including any criteria around delivery, dose, duration, intensity, co-interventions, and characteristics of complex interventions. | *MECCIR C7* (Define in advance the eligible interventions and the interventions against which these can be compared in the included studies.)  [PRISMA item 6] | 8-9 |
| R32 | Mandatory | Role of outcomes | *If measurement of particular outcomes is used as an eligibility criterion*, state and justify this. | Studies should never be excluded from a review solely because no outcomes of interest are *reported*. However, on occasion it will be appropriate to include only studies that *measured* particular outcomes. For example, a review of a multi-component school improvement intervention promoting varied increases in student outcomes might legitimately exclude studies that do not measure student achievement.  *MECCIR C8* (Clarify in advance whether outcomes listed under ’Criteria for inclusion and exclusion of studies in the review' are used as criteria for including studies (rather than as a list of the outcomes of interest within whichever studies are included).)  [PRISMA item 6] | 9-10 |
| R33 | Mandatory | Outcomes of interest | State primary and secondary outcomes of interest to the review, and define acceptable ways of measuring them. | Explain how multiple variants of outcome measures (e.g., definitions, assessors, scales, time points) are addressed. The *primary outcomes* should be as few as possible (ideally no more than three).  *MECCIR C14* (Define in advance which outcomes are primary outcomes and which are secondary outcomes.)  Also *MECCIR C15 – C18*. | 9-10 |
| Search methods for identification of studies | | | | |  |
| R34 | Mandatory | Search sources | List all sources searched, including: databases, trials registers, web sites, and grey literature. Database names should include platform/provider name and dates of coverage; web sites should include full name and URL. State whether reference lists were searched and whether individuals or organizations were contacted. | *MECCIR C36* (Document the search process in enough detail to ensure that it can be reported correctly in the review.)  *Also MECCIR C24 – C31.*  [PRISMA item 7] | 10-11 |
| R35 | Mandatory | Latest searches | Provide the date of the last search and the issue/version number (where relevant) for each database whose results were evaluated and incorporated into the review. If a search was re-run prior to publication, the results of which were not incorporated, explain how the results were dealt with and provide the date. | The review should provide the search date from which studies have been retrieved and assessed for inclusion. This is the date up to which the conclusions of the review are valid. It should reflect the date of the most recent set of searches from which all records have been screened for relevance and any studies meeting the eligibility criteria have been fully incorporated into the review (studies may be awaiting classification if, for example, the review authors are awaiting translation or clarification from authors or sponsors).  Since the review is likely to have drawn on searches conducted across multiple databases, it is possible that searches were performed on more than one date. The earliest date of the most recent set of searches should be provided in the review text and as the hard-coded date of the last search. The remaining dates for other databases should be reported in an appendix.  If a ‘catch-up’ search was run subsequent to the review being written up, any relevant studies not yet assessed for inclusion should be listed in the section ‘Studies awaiting assessment’.  *MECCIR C37* (Rerun or update searches for all relevant databases within 12 months before publication of the review or review update, and screen the results for potentially eligible studies.)  *MECCIR C38* (Incorporate fully any studies identified in the rerun or update of the search within 12 months before publication of the review or review update.)  [PRISMA item 7] | 10-11 |
| R36 | Mandatory | Search restrictions | Specify and justify any restrictions placed on the time period covered by the search. | *MECCIR C35* (Justify the use of any restrictions in the search strategy on publication date, publication format or language.) | N/A |
| R37 | Mandatory | Searches for different types of evidence | *If the review has specific eligibility criteria to include additional studies such as studies of adverse effects, economics evidence or qualitative research evidence*, describe search methods for identifying such studies. | Some reviews extend beyond a focus on the effects of interventions and address specific additional types of evidence.  *MECCIR C26* (*If the review has specific eligibility criteria around study design to address adverse effects, economic issues or qualitative research questions*, undertake searches to address them.) | N/A |
| R38 | Mandatory | Search strategies for bibliographic databases | Present the exact search strategy (or strategies) used for each database in an Appendix, including any limits and filters used, so that it could be replicated. | Search strategies that are available elsewhere (e.g., standard methodological filters, or strategies used to populate a specialized register) may be referenced rather than reproduced. Including numbers of hits for each line in the strategy is optional.  *MECCIR C36* (Document the search process in enough detail to ensure that it can be reported correctly in the review.)  Also *MECCIR C32 – C35*.  [PRISMA item 8] | 37-47 |
| R39 | Highly desirable | Search strategies for other sources | Report the search terms used to search any sources other than bibliographic databases (e.g., trials registers, the web, direct contact with primary study authors), and the dates of the searches. | Some of this information might be best placed in an Appendix.  *MECCIR C36* (Document the search process in enough detail to ensure that it can be reported correctly in the review.) | 10-11 |
| Data collection and analysis | | | | |  |
| R40 | Mandatory | Inclusion decisions | State how inclusion decisions were made (i.e. from search results to included studies), clarifying how many people were involved and whether they worked independently. | *MECCIR C39* (Highly desirable to use (at least) two people working independently to determine whether each study meets the eligibility criteria, and define in advance the process for resolving disagreements.)  [PRISMA item 9] | 11 |
| R41 | Mandatory | Data collection process | State how data were extracted from reports of included studies, clarifying how many people were involved (and whether independently), and how disagreements were handled. Describe data collection process for any reports requiring translation. | *MECCIR C43* (Use a data collection form, which has been piloted.)  *MECCIR C45* (Highly desirable to use (at least) two people working independently to extract study characteristics from reports of each study, and define in advance the process for resolving disagreements.)  [PRISMA item 10] | 11 |
| R42 | Highly desirable | Requests for data | Describe attempts to obtain or clarify data from individuals or organizations. | *MECCIR C48* (Seek key unpublished information that is missing from reports of included studies.)  [PRISMA item 10] | 8; 12; 14 |
| R43 | Mandatory | Data items | List the types of information that were sought from reports of included studies. | *MECCIR C44* (Collect characteristics of the included studies in sufficient detail to populate final tables.)  [PRISMA item 11] | 11 |
| R44 | Mandatory | Transformations of data | Explain any transformations of reported data prior to presentation in the review, along with any assumptions made. Explain any procedures for extracting numeric data from graphs. | *MECCIR C46* (Collect and utilize the most detailed numerical data that might facilitate similar analyses of included studies. Where 2×2 tables or means and standard deviations are not available, this might include effect estimates (e.g., odds ratios, regression coefficients), confidence intervals, test statistics (e.g., t, F, Z, chi-squared), or p-values, or even data for individual participants.) | 11-12 |
| R45 | Highly desirable | Missing outcome data | Explain how missing outcome data were handled. | Describe how assumptions are applied for missing data, e.g., last observation carried forward, or assumptions of particular values such as worst-case or best-case scenarios. | 12-13 |
| R46 | Mandatory | Tools to assess risk of bias/study quality in individual studies | State the tool(s) or coding strategies used to assess the primary study quality/risk of bias for included studies, how the tool(s) or coding strategies were implemented, and the criteria used to assign studies, for example, to judgments of low risk, high risk, and unclear risk of bias; low quality or high quality. | *MECCIR C51* (Assess the study quality or risk of bias for each included study. For randomized trials, the Cochrane 'Risk of bias' tool might be used, involving judgments and supports for those judgments across a series of domains of bias  *MECCIR C52 – C60.*  [PRISMA item12] | 11-12 |
| R47 Mandatory | | Using effect measures | Explain what effect measures were used in the review | State the effect measures (and their corresponding variances) used by the review authors to describe effect sizes (e.g., risk ratio, mean difference) in any included studies and/or meta-analyses. | 12 |
| R48 | Mandatory | Quantitative synthesis | Describe any methods for combining results across studies (e.g., meta-analysis, subgroup analysis, meta-regression, sensitivity analysis), including methods for assessing heterogeneity (e.g., I2, tau-squared, statistical test). Reference the software and command/macro/program used for analyses. | *MECCIR C62* (Undertake (or display) a meta-analysis only if participants, interventions, comparisons and outcomes are judged to be sufficiently similar to ensure an answer that is meaningful.)  *MECCIR C63* (Assess the presence and extent of between-study variation when undertaking a meta-analysis.)  [PRISMA items 12, 13, 14 and 16] | 13-14 |
| R49 | Mandatory | Addressing risk of bias/study quality | Describe how studies with low quality or high/variable risks of bias are addressed in the synthesis. | *MECCIR C*59 (Address risk of bias/study quality in the synthesis (whether qualitative or quantitative). For example, present analyses stratified according to key risk of bias/quality items, or restricted to studies at low risk of bias/with high quality). | 12-13 |
| R50 | Mandatory | Non-standard designs | *If designs other than individually randomized, parallel-group randomized trials are included*, describe any methods used to address clustering, matching or other design features of the included studies. | *MECCIR C70* (Consider the impact on the analysis of clustering, matching or other non-standard design features of the included studies.) | 12 |
| R51 | Mandatory | Studies with more than two groups | *If multi-arm studies are included*, explain how they are addressed and incorporated into syntheses. | *MECCIR C66* (*If multi-arm studies are included*, analyses multiple intervention groups in an appropriate way that avoids arbitrary omission of relevant groups and double-counting of participants.) | 12 |
| R52 | Highly desirable | Risk of reporting bias across studies | Describe any methods used for assessing the risk of reporting biases such as publication bias. | [PRISMA item 15] | 13 |
| R53 | Mandatory | Moderator analyses | *If moderator analysis (subgroup or meta-regression analyses) was performed*, state the potential effect modifiers with rationale for each, stating whether each was defined *a priori* or *post hoc*. | *MECCIR C22* (Pre-define potential effect modifiers (e.g., used in subgroup or meta-regression analyses) at the protocol stage; restrict these in number; and provide rationale for each.)  [PRISMA item 16] | 14 |
| R54 | Highly desirable | Summary of findings | State any methods for summarizing the findings of the review, including the assessment of the quality of the body of evidence for each outcome. | *MECCIR C75* (If applicable, include a ‘Summary of Findings’ table according to recommendations described in the Cochrane Handbook (version 5 or later). Specifically:  •include results for one population group (with few exceptions);  •indicate the intervention and the comparison intervention;  •include seven or fewer participant-important outcomes;  •describe the outcomes (e.g., scale, scores, follow-up);  •indicate the number of participants and studies for each outcome;  •present at least one baseline risk for each dichotomous outcome (e.g., study population or median/medium risk) and baseline scores for continuous outcomes (if appropriate);  •summarize the intervention effect (if appropriate); and  •include a measure of the quality of the body of evidence)  *MECCIR C76* (Use the five GRADE considerations (study limitations, consistency of effect, imprecision, indirectness and publication bias) to assess the quality of the body of evidence for each outcome, and to draw conclusions about the quality of evidence within the text of the review.)  [PRISMA item 12] | 4-5; 14 |
| Results | | | | |  |
| Description of studies | | | | |  |
| R55 | Mandatory | Flow of studies | Provide information on the flow of studies from the number(s) of references identified in the search to the number of studies included in the review, ideally using a flow chart. Clarify how multiple references for the same study relate to the individual studies. | *MECCIR C41* (Document the selection process in sufficient detail to complete a PRISMA flow chart and a table of ‘Characteristics of excluded studies’.  *(MECCIR C42* (Collate multiple reports of the same study, so that each study rather than each report is the unit of interest in the review.)  [PRISMA item 17] | 104-105 |
| R56 | Highly desirable | Lack of included studies | *If a review identifies no eligible studies*, restrict the Results section to a description of the flow of studies and any brief comments about reasons for exclusion of studies. | Under ‘Risk of bias/quality in included studies’ and ‘Effects of interventions’, state “No study met the eligibility criteria’. Any discussion of evidence not meeting the eligibility criteria of the review should be in the Discussion section. | N/A |
| R57 | Mandatory | Excluded studies | List key excluded studies (i.e., those a reader might reasonably have expected to find) and provide justification for each exclusion. | The table of ‘Characteristics of excluded studies’ is intended as an aid to users rather than a comprehensive list of studies that were identified but not included. List here any studies that a user might reasonably expect to find in the review to explain why it is excluded. | 16 |
|  | | | | |  |
| R58 | Highly desirable | Studies awaiting classification | List the characteristics of any studies that have been identified as potentially eligible but have not been incorporated into the review. | Users of the review will be interested to learn of any potentially relevant studies that have been conducted which are known to the review team but have not yet been incorporated in to the review. This will help them to assess the stability of the review findings. These should be listed in the table of ‘Characteristics of studies awaiting classification’, along with any details that are known. | 31-35 |
| R59 | Highly desirable | Ongoing studies | Provide details of any identified studies that have not been completed. | Users of the review will be interested to learn of any potentially relevant studies that have not been completed. This will help them to assess the stability of the review findings. These should be listed in the table of ‘Characteristics of ongoing studies’, along with any details that are known. | 37 |
| R60 | Highly desirable | Table of ‘Characteristics of included studies’ | It is highly desirable to present a table of ‘Characteristics of included studies’ using a uniform format across all studies. For large reviews including hundreds of studies, such a table may not be appropriate, in which case the review should include detailed descriptive statistics for the included studies. | *MECCIR C44* (Collect characteristics of the included studies in sufficient detail to populate final tables)  [PRISMA item 18] | 97-103 |
| R61 | Mandatory | Included studies | Provide a brief narrative summary of any included studies. This should include the number of participants and a summary of the characteristics of the study populations and settings, interventions, comparators and funding sources. |  | 14-16; 97-103 |
| R62 | Highly desirable | Table of ‘Characteristics of included studies’: sample sizes | Include the sample size (and effective sample size for cluster assigned studies) for each included study in the table of ‘Characteristics of included studies’. | If sample sizes are available for each intervention group, these should be included. A convenient place is often within the box for Interventions (e.g., inserting “(n=50))” after each listed intervention group. | 97-103 |
| R63 | Highly desirable | Table of ‘Characteristics of included studies’: methods | Provide the basic study design or design features (e.g., parallel group randomized trial, cluster-randomized trial, controlled before and after study). | Even if the review is restricted to one study design, these tables should provide a comprehensive summary of each study.  It is important that labels used to describe study designs are clearly defined in the review.  [PRISMA item 18] |  |
| R64 | Highly desirable | Table of ‘Characteristics of included studies’: participants | Provide sufficient information about the study populations to enable a user of the review to assess the applicability of the review’s findings to their own setting. | Information presented in this table should reflect the baseline demographics of the study sample. [PRISMA item 18] | 97-103 |
| R65 | Highly desirable | Table of ‘Characteristics of included studies’: interventions | Provide sufficient information to enable users of the review to assess the applicability of the intervention to their own setting, and if possible in a way that allows the intervention to be replicated. | For example, for education interventions consider intervention, educational context, frequency and duration of doses; or for complex interventions, specify the core components of the intervention. Lengthy explanations of interventions should be avoided. Citations to sources of detailed descriptions can be included.  [PRISMA item 18] | 97-103 |
| R66 | Highly desirable | Table of ‘Characteristics of included studies’: outcomes | Provide clear and consistent information about outcomes measured (or reported), how they were measured and the times at which they were measured. | It should be clear whether main outcomes of interest in the review were measured in the study. | 97-103 |
| R67 | Highly desirable | Table of ‘Characteristics of included studies’: dates | Include the dates when the study was conducted in the table of ‘Characteristics of included studies’. | If dates are not available then this should be stated (e.g., “Study dates not reported”).  [PRISMA item 18] | 97-103 |
| R68 | Highly desirable | Table of ‘Characteristics of included studies’: funding source | Include details of funding sources for the study, where available. | Details of funding sources should be placed in this table. Including an extra row in the table of ‘Characteristics of included studies’ is encouraged. | 97-103 |
| R69 | Highly desirable | Table of ‘Characteristics of included studies’: declarations of interest | Include details of any declarations of interest among the primary researchers. | Declarations of interest should be placed in this table. Including an extra row in the table of ‘Characteristics of included studies’ is encouraged. |  |
| R70 | Highly desirable | Choice of intervention groups in multi-arm studies | *If a study is included with more than two intervention arms*, restrict comments on any irrelevant arms to a brief comment in the table of ‘Characteristics of included studies’. | Intervention arms that are not relevant to the review question should not be discussed in detail, although it is useful to clarify (in this table) that such arms were present.  *MECCIR C49* (*If a study is included with more than two intervention arms*, include in the review only intervention and control groups that meet the eligibility criteria.) |  |
| R71 | Mandatory | References to included studies | List all reports of each included study under the relevant Study ID. | [PRISMA item 18] | 58-64 |
| Study quality/risk of bias in included studies | | | | |  |
| R72 | Mandatory | ‘Risk of bias’ and/or study quality table | Present a ‘Risk of bias’ and/or “Study Quality” table for each included study, with judgments about risks of bias, and explicit supports for these judgments. | *MECCIR C51:* Assess the risk of bias/study quality for each included study. If the review is co-registered or is reviewing randomized trials, the Cochrane 'Risk of bias' tool should be used, involving judgments and supports for those judgments across a series of domains of bias, as described the Cochrane Handbook (version 5 or later).If not, then one of the many other risk of bias and/or study quality tools should be utilized and detailed within the protocol prior to implementation.  Also *MECCIR C53 – C60*  [PRISMA item 19] | 107-110 |
| R73 | Highly desirable | Summary assessments of risk of bias/study quality | Summarize the study quality/risk of bias across domains for each key outcome for each included study, and ensure that these are supported by the information presented in the ‘Risk of bias’ and/or “Study Quality” tables. | *MECCIR C58* (Summarize the risk of bias/study quality for each key outcome for each study.)  [PRISMA item 22] | 107 |
| R74 | Mandatory | Study quality/risk of bias in included studies | Provide a brief narrative summary of the quality/risks of bias among the included studies [PRISMA items 22 and 25] | It may be helpful to identify any studies considered to be at low risk of bias for particular key outcomes or with high quality for specific methodological characteristics. | 16-17 |
| Effects of interventions | | | | |  |
| R75 | Mandatory | Use of ‘Data and analysis’ headings | Ensure appropriate use of the hierarchy of Comparisons / Outcomes / Subgroups / Study data in the ‘Data and analysis’ section. | Appropriate use of the hierarchy ensures consistency of structure across reviews. It is confusing for the user if outcomes are listed against the heading ‘Comparison’ and interventions listed against the heading ‘Outcome or subgroup’. | 17-19 |
| R76 | Highly desirable | Presenting data | Ensure that simple summary data for each intervention group, as well as estimates of effect size (comparing the intervention groups), are available for each study for each outcome of interest to the review. | Simple summaries such as numbers of events, means and standard deviations should be presented for each treatment group when available. This is achieved primarily by using the ‘Data and analyses’ section of the review, for dichotomous and continuous outcomes. For other outcomes, these should typically be presented in tables of ‘Other data’.  [PRISMA item 20] |  |
| R77 | Mandatory | Number of studies and participants | State how many studies and how many participants contributed data to results for each outcome, along with the proportion of the included studies and recruited participants potentially available for the relevant comparison. | It is unlikely that the same number of studies will contribute data to every outcome of interest. Specific studies may contribute different numbers of participants for different outcomes. Therefore, for each comparison, it is helpful to indicate to readers what proportion of the relevant included studies and recruited participants contribute data to each outcome. Failing to disclose this may be misleading.  [PRISMA item 9] | 4-5; 15-16; 105 |
| R78 | Highly desirable | Source of data | State the source of all data presented in the review, in particular, whether it was obtained from published literature, by correspondence, from a trials register, from a web-based data repository, etc. | Transparency of data source enables validation or verification of data by others including editors or readers of the review. |  |
| R79 | Mandatory | Multiple outcome data | Describe any *post hoc* decisions that might give rise to accusations of selective outcome reporting, for example when there are multiple outcome measures (e.g., different scales), multiple time points or multiple ways of presenting results. | Transparent disclosure of post-hoc decisions will enable readers of the review to assess the credibility of the results of the review for themselves.  *MECCIR C16* (Define in advance details of what are acceptable outcome measures (e.g., diagnostic criteria, scales, composite outcomes).)  *MECCIR C17* (Define in advance how outcome measures will be selected when there are several possible measures (e.g., multiple definitions, assessors, or scales)).  *MECCIR C18* (Define in advance the timing of outcome measurement.) | 9 |
| R80 | Highly desirable | Ordering of results and ‘Data and analysis’ section | Organize results to follow the order of comparisons and outcomes specified in the protocol, following in particular the distinction between primary and secondary outcomes. | Review authors must avoid selectively reporting analysis results in a way that depends on the findings. The best way to achieve this is to follow a well-structured protocol and present results as outlined in that protocol. However, sometimes a pragmatic decision needs to be made that an alternative arrangement is preferable, particularly with regard to comparisons. This choice should be explicitly justified. | 17-19 |
| R81 | Mandatory | Pre-specified outcomes | Report synthesis results for all pre-specified outcomes, irrespective of the strength or direction of the result. Indicate whether data were not available for outcomes of interest, including whether harms were identified. | To avoid selective outcome reporting (in truth or in perception), the review should address all outcomes specified in the protocol.  [PRISMA item 20] | 17-19 |
| R82 | Mandatory | Statistical uncertainty | Accompany all effect size estimates with a measure of statistical uncertainty (e.g., a confidence interval with a specified level of confidence such as 90%, 95% or 99%). | Confidence intervals are the preferred method for expressing statistical uncertainty.  [PRISMA item 20] | 4-5; 17-19 |
| R83 | Highly desirable | P values | *If reporting p-values*, provide exact p-values (e.g., p = 0.08 rather than p > 0.05). | Effect estimates with confidence intervals are the preferred method of presenting numeric results. P-values should not be used as an alternative to confidence intervals and should not be used to divide results into ‘significant’ or ‘non-significant’. | N/A |
| R84 | Mandatory | Tables and Figures | Link to each Table and Figure. |  | 9-19 |
| R85 | Highly desirable | Number of Tables and Figures | Restrict the number of Tables and Figures to a small number (six or less) to convey key findings without affecting the readability of the review text. | Tables and Figures may be added to reviews and included in the body of the text. Reviews should try to avoid including a large number of Tables and Figures, unless they are necessary and appropriate to display review findings. Additional or supplementary Tables and Figures can be included as appendices, as appropriate. |  |
| R86 | Mandatory | Consistency of results | Ensure that all statistical results presented in the main review text are consistent between the text and the ‘Data and analysis’ tables. | MECCIR C47, C50 | 17-19; 103-117 |
| R87 | Mandatory | Different scales | Explain how studies measuring an outcome of interest using different scales (such as alternative rating scales that measure symptoms or behavior) were combined, stating whether positive or negative values reflect benefit or harm. | If data from different scales are combined and presented on a standardized scale (such as a standardized mean difference), it is important to clarify that a positive effect size has the same meaning for every study. The direction of benefit or harm must be stated.  *MECCIR C61* (*If studies are combined with different scales*, ensure that higher scores for continuous outcomes all have the same meaning for any particular outcome; explain the direction of interpretation; and report when directions were reversed. ) | 2; 12 |
| R88 | Mandatory | Interpretability of results | Ensure that key findings are interpretable, or are re-expressed in an interpretable way. For instance, they might be re-expressed in absolute terms (e.g., assumed and corresponding risks, NNTs, group means), and outcomes combined with a standardized scale (e.g., SMD) might be re-expressed in units that are more naturally understood. If substantively or clinically important effect sizes are well understood, these should be provided to aid interpretation. Relying on Cohen’s (1988) guidelines is not recommended given that effect sizes are generally context specific. | Absolute effects provide a useful illustration of the likely impact of intervention, and are usually easier to understand than relative effects. They may need to be accompanied, however, with information about assumed baseline risks. Confidence intervals should be presented for NNTs and similar summary measures. Re-expressing relative effects as absolute effects often requires the specification of assumed (e.g., untreated) risks, and the source of these should be provided. Results expressed as standardized mean differences reflect the number of standard deviations’ difference between mean responses. This is not intuitive to many readers who may be more familiar with specific scales. Substantively or clinically important effect sizes should ideally be specified in the protocol.  MECCIR C69 (consider statistical heterogeneity in interpretation)  MECCIR C72 (interpret p-values correctly) | 19 |
| R89 | Mandatory | Studies without usable data | Comment on the potential impact of studies that apparently measured outcomes but did not contribute data that allowed the study to be included in syntheses. | There is good evidence of selective outcome reporting among clinical trials. Outcomes that are believed to have been measured but are not reported in a usable format may therefore be systematically different from those that are usable, introducing bias. ‘Usable’ in this sense refers both to incorporation in a meta-analysis and to consideration in non-statistical syntheses of findings. Authors might consider using a table to indicate which studies contribute data to the outcomes of interest in the review.  *MECCIR C40* (Include studies in the review irrespective of whether measured outcome data are reported in a ‘usable’ way. | 16 |
| R90 | Highly desirable | Missing outcome data | Discuss the implications of missing outcome data from individual participants (due to losses to follow up or exclusions from analysis). | *MECCIR C64* (Consider the implications of missing outcome data from individual participants (due to losses to follow up or exclusions from analysis).) | 17 |
| R91 | Highly desirable | Skewed data | Discuss the possibility and implications of skewed data when analyzing continuous outcomes. | *MECCIR C65* (Consider the possibility and implications of skewed data when analyzing continuous outcomes) |  |
| R92 | Highly desirable | Forest plots | Present data from multiple studies in forest plots wherever possible, providing it is reasonable to do so. | Presenting data in forest plots can be useful even if the studies are not combined in a meta-analysis.  [PRISMA item 20] | 111-117 |
| R93 | Highly desirable | Multiple subgroup analyses and sensitivity analyses | *If presenting multiple sensitivity analyses or different ways of subgrouping the same studies*, present these in summary form (e.g., a single Table or Figure) and not in multiple forest plots. | [PRISMA item 23]  MECCIR C67, C68, C71 | 117 |
| R94 | Mandatory | Labels on plots | Label the directions of effect and the intervention groups in forest plots with the interventions being compared. | Directions of effect should be used as consistently as possible within a review. | 111-117 |
| R95 | Highly desirable | Study quality/risk of bias across studies | Present results of the assessment of study quality/risk of bias across studies (and across domains) for each key outcome and state whether this leads to concerns about the validity of the review’s findings. | Considerations of study quality/risk of bias across studies are required for assessments of the quality of the body of evidence (e.g., using GRADE).  [PRISMA item 22] | 16-17 |
| R96 | Highly desirable | Reporting biases | Present results of any assessment of the potential impact of reporting biases on the review’s findings. | *MECCIR C73* (Consider the potential impact of reporting biases on the results of the review or the meta-analyses it contains.)  [PRISMA item 22] | 17 |
| R97 | Optional | ‘Summary of findings’ table | If the Cochrane risk of bias tool is used, present a ‘Summary of Findings’ table according to recommendations described in the Cochrane Handbook (version 5 or later). Specifically:  include results for one clearly defined population group (with few exceptions); indicate the intervention and the comparison intervention;  include seven or fewer participant-important outcomes;  describe the outcomes (e.g., scale, scores, follow-up);  indicate the number of participants and studies for each outcome;  present at least one baseline risk for each dichotomous outcome (e.g., study population or median/medium risk) and baseline scores for continuous outcomes (if appropriate);  summarize the intervention effect (if appropriate); and include a measure of the quality of the body of evidence for each outcome. | *MECCIR C74* (Include a ‘Summary of Findings’ table according to recommendations described in the Cochrane Handbook (version 5 or later). Specifically:  •include results for one population group (with few exceptions);  •indicate the intervention and the comparison intervention;  •include seven or fewer participant-important outcomes;  •describe the outcomes (e.g., scale, scores, follow-up);  •indicate the number of participants and studies for each outcome;  •present at least one baseline risk for each dichotomous outcome (e.g., study population or median/medium risk) and baseline scores for continuous outcomes (if appropriate);  •summarize the intervention effect (if appropriate); and  •include a measure of the quality of the body of evidence.)  [PRISMA item 24] | 4-5 |
| R98 | Optional | Assessments of the quality of the body of evidence | Provide justification or rationale for any measures of the quality of the body of evidence for each key outcome. If a ‘Summary of findings’ table is used, use footnotes to explain any downgrading or upgrading. | *MECCIR C75* (Use the five GRADE considerations (study limitations, consistency of effect, imprecision, indirectness and publication bias) to assess the quality of the body of evidence for each outcome, and to draw conclusions about the quality of evidence within the text of the review.)  *MECCIR C76* (Justify and document all assessments of the quality of the body of evidence (for example downgrading or upgrading if using the GRADE tool).) | 17-19 |
| Discussion | | | | |  |
| R99 | Highly desirable | Discussion headings | Include the standard headings when writing the Discussion. | Six standard headings are recommended (‘Summary of main results’, ‘Overall completeness and applicability of evidence’, ‘Quality of the evidence’, ‘Potential biases in the review process, ‘Agreements and disagreements with other studies or reviews’). | 19-20 |
| R100 | Mandatory | Limitations | Discuss limitations of the review (e.g., incomplete identification of studies, reporting bias), and the implications of any study-level or outcome-level risk of bias/quality assessments on the review findings. | Review authors must explicitly state the limitations of their review. These limitations should be addressed in the discussion headings of ‘Quality of the evidence’ and ‘Potential biases in the review process.’ If those two headings are not used, then at minimum, the review should include a ‘Limitations’ heading that addresses all potential review limitations.  *MECCIR C73* (Consider the potential impact of reporting biases on the results of the review or the meta-analyses it contains.)  [PRISMA item 25] | 19-20 |
| Authors’ conclusions | | | | |  |
| R101 | Mandatory | Conclusions: implications for practice | Provide a general interpretation of the evidence so that it can inform practice or policy decisions. Avoid making recommendations for practice. | *MECCIR C77-78* (Avoid providing recommendations for practice.) | 20 |
| R102 | Mandatory | Conclusions: implications for research | *If recommending further research*, structure the implications for research to address the nature of evidence required, including population, intervention comparison, outcome, and type of study. | Researchers and research funders are an important user group of Campbell reviews. Recommendations for future research should offer constructive guidance on addressing the remaining uncertainties identified by the review. This is particularly important for reviews that identify few or no studies.  *MECCIR C79* (Structure the implications for research to address the nature of evidence required, including population intervention comparison, outcome, and type of study). | 20-21 |
| Acknowledgements | | | | |  |
| R103 | Mandatory | Acknowledgements | Acknowledge the contribution of people not listed as authors of the review, including any assistance from Campbell Coordinating Groups, non-author contributions to searching, data collection, study appraisal or statistical analysis, and the role of any funders. | [PRISMA item 27] | 21 |
| Contributions of authors | | | | |  |
| R104 | Mandatory | Contributions of authors | Describe the contributions of each author. |  | 21 |
| Declarations of interest | | | | |  |
| R105 | Mandatory | Declarations of interests | Report any present or past affiliations or other involvement in any organization or entity with an interest in the review’s findings that might lead to a real or perceived conflict of interest. | The nature and extent of the affiliation or involvement (whether financial or non-financial) should be described. An additional consideration for authors of systematic reviews is the declaration of involvement in studies that were included in the review. It is important to note that authors who were involved in primary studies must not be involved in the data extraction/coding/critical appraisal of those studies as part of the systematic review process. | 21 |
| Differences between protocol and review | | | | |  |
| R106 | Mandatory | Changes from the protocol | Explain and justify any changes from the protocol (including any *post hoc* decisions about eligibility criteria or the addition of subgroup or moderator analyses). | *MECCIR C13* (Justify any changes to eligibility criteria or outcomes studied. In particular, post hoc decisions about inclusion or exclusion of studies should keep faith with the objectives of the review rather than with arbitrary rules.) | 21-22 |
| R107 | Highly desirable | Methods not implemented | Document aspects of the protocol that were not implemented (e.g., because no studies, or few studies, were found) in the section ‘Differences between protocol and review’, rather than in the Methods Section. |  | 22 |
| Sources of support | | | | |  |
| R108 | Mandatory | Sources of support | List sources of financial and non-financial support for the review and the role of the funder, if any. | [PRISMA item 28] | 21 |
