# Supplementary material for: A comparison of the effectiveness of cognitive behavioural interventions based on delivery features for elevated symptoms of depression in adolescents: A systematic review
Source: Campbell Syst Rev. 2024 Jan 7;20(1):e1376. doi: 10.1002/cl2.1376 (PMC10771715; doi:10.1002/cl2.1376)
Supplement: Supplementary file 2 — Supporting information. [file CL2-20-e1376-s002.docx]

**Characteristics of excluded studies [ordered by study ID]**

| Study | Reason for exclusion |
| --- | --- |
| Aboustate 2019 | Wrong analysis |
| Ackerson 1993 | Paper not found |
| ACTRN12610000074099 | Wrong analysis |
| ACTRN12616001626459 | Wrong study design |
| Afuwape 2010 | Wrong patient population |
| Aitken 2019 | Wrong analysis |
| Alavi 2016 | Wrong patient population |
| Alegria 2014 | Wrong patient population |
| Alvarez 2008 | Wrong patient population |
| Amaya 2011 | Wrong analysis |
| Ammerman 2012 | Wrong patient population |
| Ammerman 2013a | Wrong patient population |
| Ammerman 2013b | Wrong patient population |
| Ammerman 2015 | Wrong patient population |
| Anderson 2014 | Wrong patient population |
| Appleby 1998 | Wrong patient population |
| Aqabozorg 2012 | Paper not found |
| Arnarson 2011 | Wrong patient population |
| Arnow 2003 | Wrong patient population |
| Asarnow 2002 | Wrong patient population |
| Azevedo 2017 | Wrong patient population |
| Barnes 2013 | Wrong patient population |
| Barry 2017 | Wrong patient population |
| Bass 2006 | Wrong patient population |
| Beattie 2009 | Wrong study design |
| Bedoya 2014 | Wrong patient population |
| Beeber 2010 | Wrong patient population |
| Bernal 2005 | Wrong comparator |
| Bernhardsdottir 2013 | Wrong patient population |
| Beutel 2012 | Wrong study design |
| Blanco 2014 | Wrong patient population |
| Bollenbach 1983 | Wrong intervention |
| Boogar 2012 | Paper not found |
| Boschloo 2019 | Wrong patient population |
| Boylan 2006 | Wrong analysis |
| Brent 1996 | Wrong analysis |
| Brent 1997a | Wrong analysis |
| Brent 1998 | Wrong analysis |
| Brent 1999 | Wrong analysis |
| Brent 2015 | Wrong study design |
| Brière 2014 | Wrong analysis |
| Brière 2016 | Wrong analysis |
| Browning 2011 | Wrong patient population |
| Bruijniks 2015 | Wrong patient population |
| Brunwasser 2018 | Wrong patient population |
| Burns 2014 | Wrong analysis |
| Buszewicz 2010 | Wrong patient population |
| Butler 1980 | Wrong study design |
| Cabiya 2008 | Wrong patient population |
| Calear 2009 | Wrong patient population |
| Calear 2013 | Wrong analysis |
| Calear 2018 | Wrong analysis |
| Carta 2012 | Wrong patient population |
| Carty 2001 | Wrong study design |
| Cavanagh 2006 | Wrong patient population |
| Cavanagh 2011 | Wrong study design |
| Chan 2015 | Wrong patient population |
| Chaplin 2006 | Wrong patient population |
| Chen 2015 | Wrong patient population |
| Chen 2018 | Wrong intervention |
| ChiCTR1900023145 | Paper not found |
| Chirita 2006 | Wrong patient population |
| Christensen 2004 | Wrong patient population |
| Christensen 2006 | Wrong patient population |
| Christensen 2013 | Wrong patient population |
| Chu 2016 | Wrong intervention |
| Clarke 1992 | Wrong analysis |
| Clarke 2001a | Wrong analysis |
| Clarke 2005 | Wrong intervention |
| Clarke 2009 | Wrong patient population |
| Clarke 2014 | Wrong patient population |
| Coats 1982 | Paper not found |
| Collado 2016 | Wrong intervention |
| Compas 2011 | Wrong patient population |
| Compas 2015 | Wrong patient population |
| Conradi 2008 | Wrong patient population |
| Cook 2016 | Wrong patient population |
| CTRI/2012/10/003043 | Paper not found |
| Curry 2006 | Wrong analysis |
| Curry 2011 | Wrong outcomes |
| Dana 1998 | Wrong patient population |
| David 2018 | Wrong patient population |
| David 2019 | Wrong patient population |
| Day 2013 | Wrong patient population |
| de Graaf 2009 | Wrong patient population |
| de Graaf 2010 | Wrong patient population |
| De Jonge-Heesen 2020 | Wrong patient population |
| Dean 2016 | Wrong patient population |
| Dear 2018 | Wrong patient population |
| Dickerson 2018 | Wrong outcomes |
| Dietz 2014 | Wrong comparator |
| DiFonte 2016 | Wrong analysis |
| Donker 2013 | Wrong patient population |
| DRKS00020941 | Wrong analysis |
| Duong 2016 | Wrong patient population |
| Emslie 2015 | Wrong intervention |
| Ezeudu 2019 | Wrong patient population |
| Fawcett 2020 | Wrong patient population |
| Feehan 1996 | Wrong analysis |
| Fine 1993 | Wrong study design |
| Fletcher 2005 | Wrong patient population |
| Forehand 2012 | Wrong patient population |
| Freres 2002 | Paper not found |
| Gau 2012 | Wrong analysis |
| Gega 2013 | Wrong study design |
| Geisner 2006 | Wrong patient population |
| Geisner 2015 | Wrong patient population |
| Gerhards 2010 | Wrong patient population |
| Gijzen 2018 | Wrong patient population |
| Gillham 1995 | Wrong study design |
| Gillham 1999 | Wrong patient population |
| Gillham 2006 | Wrong intervention |
| Gillham 2006a | Wrong patient population |
| Gillham 2007 | Wrong patient population |
| Gillham 2012 | Wrong patient population |
| Givi 2012 | Wrong study design |
| Gladstone 2020 | Wrong comparator |
| Gollan 2003 | Wrong analysis |
| Gonzalez 2007 | Wrong patient population |
| Goodyer 2011 | Wrong analysis |
| Goodyer 2017 | Wrong comparator |
| Goossens 2016 | Wrong intervention |
| Gordon 2011 | Wrong study design |
| Griffiths 2012 | Wrong patient population |
| Haeffel 2010 | Wrong patient population |
| Haeffel 2017 | Wrong patient population |
| Hallgren 2015 | Wrong patient population |
| Hamdan-Mansour 2009 | Wrong patient population |
| He 2019 | Wrong patient population |
| Hoek 2011 | Wrong comparator |
| Hoek 2012 | Wrong intervention |
| Hollandare 2013 | Wrong patient population |
| Horowitz 2007 | Wrong patient population |
| Hur 2018 | Wrong patient population |
| Hvenegaard 2015 | Wrong study design |
| Hyun 2005 | Wrong patient population |
| Hyun 2010 | Wrong patient population |
| Iftene 2015 | Wrong comparator |
| Imber 1990 | Wrong patient population |
| Ingram 2012 | Wrong study design |
| Isaacs 2017 | Wrong analysis |
| ISRCTN95425657 | Wrong analysis |
| Jaycox 1994 | Wrong study design |
| John 2011 | Wrong analysis |
| Kahn 1990 | Paper not found |
| Karami 2013 | Paper not found |
| Kennard 2009 | Wrong analysis |
| Kennard 2009a | Wrong comparator |
| Keshi 2013 | Paper not found |
| Kessler 2009 | Wrong patient population |
| Kim 2011 | Wrong study design |
| Kim 2015 | Wrong study design |
| Kindt 2014 | Wrong patient population |
| Kindt 2016 | Wrong outcomes |
| Kirkpatrick 1977 | Wrong study design |
| Koeser 2013 | Wrong study design |
| Kowalenko 2005 | Wrong study design |
| Kratochvil 2006 | Wrong analysis |
| Kumar 2015 | Wrong patient population |
| Kumara 2016 | Paper not found |
| Kuosmanen 2017 | Wrong patient population |
| Kurki 2011 | Paper not found |
| Langley 2015 | Wrong patient population |
| Lee 2020 | Wrong study design |
| Lewinsohn 1994 | Wrong study design |
| Lewinsohn 1996 | Wrong analysis |
| Lewis 2013 | Wrong intervention |
| Lillevoll 2014 | Wrong patient population |
| Lopes 2014 | Wrong patient population |
| Lorentzen 2020 | Wrong intervention |
| Lucassen 2012 | Wrong intervention |
| Luty 2007 | Wrong patient population |
| Ly 2015 | Wrong patient population |
| Lynch 1997 | Wrong patient population |
| Lynch 2005 | Wrong outcomes |
| Manassis 2010 | Wrong patient population |
| March 2006 | Wrong outcomes |
| March 2006a | Wrong analysis |
| Marcotte 1993a | Paper not found |
| Martinez 2014 | Wrong comparator |
| Matos 2019 | Wrong study design |
| McCarty 2013 | Wrong comparator |
| McCauley 2016 | Wrong intervention |
| McCloud 2020 | Wrong patient population |
| McKee 2014 | Wrong patient population |
| McNamara 1985 | Wrong patient population |
| Melnyk 2013 | Wrong patient population |
| Melnyk 2015 | Wrong patient population |
| Michelson 2016 | Wrong analysis |
| Miller 1999 | Wrong study design |
| Minor 1988 | Paper not found |
| Mohr 2013 | Wrong patient population |
| Moldovan 2013 | Wrong patient population |
| Mondin 2015 | Wrong patient population |
| Moreira 2015 | Wrong patient population |
| Muller 2015 | Wrong analysis |
| Mullin 2015 | Wrong patient population |
| Munoz 1995 | Wrong patient population |
| Murphy 1995 | Wrong patient population |
| Musiat 2014 | Wrong patient population |
| Naeem 2014 | Wrong patient population |
| Narimany 2002 | Paper not found |
| Nauta 2012 | Wrong analysis |
| Naylor 2010 | Wrong patient population |
| NCT00061698 | Wrong intervention |
| NCT00071513 | Wrong patient population |
| NCT00374439 | Wrong patient population |
| NCT00611052 | Wrong analysis |
| NCT00641368 | Wrong patient population |
| NCT01220635 | Wrong patient population |
| NCT01228890 | Wrong analysis |
| NCT02072304 | Paper not found |
| NCT02266693 | Wrong patient population |
| NCT02332239 | Wrong patient population |
| NCT02377011 | Wrong patient population |
| NCT02780232 | Wrong intervention |
| NCT03047512 | Wrong intervention |
| NCT03438331 | Wrong patient population |
| NCT03655067 | Wrong patient population |
| NCT04264585 | Wrong patient population |
| NCT04290754 | Wrong comparator |
| Nelson 2006 | Wrong study design |
| Newby 2014 | Wrong patient population |
| Nobel 2012 | Wrong patient population |
| Noel 2013 | Paper not found |
| O'Kearney 2006 | Wrong patient population |
| O'Kearney 2009 | Wrong study design |
| Ofoegbu 2020 | Wrong patient population |
| Oikawa 2006 | Paper not found |
| Onuigbo 2019 | Wrong patient population |
| Ouyang 2001 | Paper not found |
| Pace 1993 | Wrong patient population |
| Pardini 2014 | Wrong patient population |
| Parker 2016 | Wrong intervention |
| Patras 2016 | Wrong patient population |
| Pattison 2001 | Wrong patient population |
| Peden 2000 | Wrong patient population |
| Pile 2018 | Wrong analysis |
| Pinto 2013 | Wrong patient population |
| Platt 2014 | Wrong analysis |
| Poppelaars 2016 | Wrong patient population |
| Possel 2006 | Wrong patient population |
| Possel 2008 | Wrong patient population |
| Possel 2013 | Wrong patient population |
| Proudfoot 2013 | Wrong patient population |
| Puskar 2003 | Wrong patient population |
| Quilty 2013 | Wrong patient population |
| Rasing 2013 | Wrong study design |
| Redzic 2014 | Wrong patient population |
| Reivich 1996 | Wrong patient population |
| Ren 2016 | Paper not found |
| Rhodes 2014 | Wrong patient population |
| Richards 2003 | Wrong patient population |
| Richards 2013 | Wrong patient population |
| Rivet-Duval 2011 | Wrong patient population |
| Roberts 2003 | Wrong patient population |
| Roberts 2004 | Wrong patient population |
| Robinson 2005 | Paper not found |
| Rohde 2001 | Wrong analysis |
| Rohde 2008 | Wrong analysis |
| Rohde 2012 | Wrong analysis |
| Rohde 2012a | Wrong analysis |
| Rossello 1996 | Paper not found |
| Rossello 2008 | Wrong analysis |
| Rossello 2012 | Wrong analysis |
| Rush 1978 | Wrong patient population |
| Ruwaard 2009 | Wrong patient population |
| Saelid 2017 | Wrong patient population |
| Salamanca-Sanabria 2020 | Wrong patient population |
| Sanchez-Hernandez 2019 | Wrong patient population |
| Saravanan 2017 | Wrong patient population |
| Saulsberry 2013 | Wrong intervention |
| Saulsberry 2013a | Wrong intervention |
| Saw 2020b | Paper not found |
| Schramm 2011 | Wrong patient population |
| Scott 1997 | Wrong patient population |
| Scott 1999 | Paper not found |
| Sekizaki 2019 | Wrong patient population |
| Seligman 2007 | Wrong patient population |
| Selmi 1990 | Wrong patient population |
| Shakehnia 2012 | Paper not found |
| Shandley 2010 | Wrong study design |
| Shatte 1996 | Wrong patient population |
| Shin 2020 | Paper not found |
| Shiraishi 2005 | Wrong patient population |
| Shirk 2014 | Wrong intervention |
| Singhal 2014 | Wrong study design |
| Smith 2015 | Wrong patient population |
| Snow 2000 | Paper not found |
| Songprakun 2012 | Wrong patient population |
| Spence 2003 | Wrong patient population |
| Spirito 2015 | Wrong comparator |
| Stallard 2010 | Wrong patient population |
| Stallard 2011 | Wrong patient population |
| Stant 2008 | Wrong patient population |
| Stark 1987 | Wrong intervention |
| Stikkelbroek 2013 | Wrong analysis |
| Stiles-Shields 2014 | Wrong patient population |
| Stoppelbein 2003 | Wrong patient population |
| Straub 2015 | Paper not found |
| Szigethy 2007 | Wrong patient population |
| Szigethy 2014 | Wrong patient population |
| Tak 2016 | Wrong patient population |
| Takagaki 2016 | Wrong patient population |
| Tellier 1999 | Paper not found |
| Thomas 1987 | Wrong patient population |
| Thompson 2000 | Wrong patient population |
| Titov 2010 | Wrong patient population |
| Tomyn 2016 | Wrong study design |
| Topper 2017 | Wrong patient population |
| Treatment 2009 | Wrong comparator |
| Treutiger 2013 | Paper not found |
| van der Zanden 2011 | Wrong patient population |
| van der Zanden 2012 | Wrong patient population |
| van der Zanden 2014 | Wrong patient population |
| van Rensburg 1988 | Paper not found |
| Van Schaik 2008 | Paper not found |
| van Straten 2008 | Wrong patient population |
| VanVoorhees 2008 | Wrong intervention |
| Vernmark 2010 | Wrong patient population |
| Vitiello 2004 | Paper not found |
| Wagner 2014 | Wrong patient population |
| Walker 2014 | Wrong patient population |
| Wang 2016 | Wrong study design |
| Ward 2000 | Wrong patient population |
| Weersing 2003 | Paper not found |
| Weersing 2017 | Wrong patient population |
| Weinberg 1978 | Wrong patient population |
| Weisz 1997 | Wrong patient population |
| Weisz 2009 | Wrong patient population |
| Weisz 2012 | Wrong patient population |
| Whittaker 2017 | Wrong patient population |
| Wierzbicki 1987 | Wrong study design |
| Wilde 1994 | Wrong patient population |
| Willemse 2004 | Wrong patient population |
| Williams 1988 | Paper not found |
| Wong 2008 | Wrong patient population |
| Wood 1996 | Wrong comparator |
| Wood 1999 | Wrong comparator |
| Wright 2005 | Wrong patient population |
| Xu 2017 | Wrong patient population |
| Yang 2014 | Paper not found |
| Yu 2002 | Wrong intervention |
| Yusoff 2015 | Wrong patient population |
| Zamirinejad 2014 | Wrong patient population |
| Zaunműller 2014 | Wrong patient population |
| Zemestani 2017 | Wrong patient population |
| Zou 2017 | Wrong patient population |

**Characteristics of studies awaiting classification [ordered by study ID]**

**Demir 2019**

| Methods | Randomised controlled trial |
| --- | --- |
| Participants | 18-25 year olds with mild or moderate depressive symptoms as measured by the Beck Depression Inventory |
| Interventions | 1) Cognitive Behavioral Therapy-Based Group Counseling  2) Control - no treatment |
| Outcomes | Primary: depressive symptoms measured by the Beck Depression Inventory, anxiety symptoms measured by the Beck Anxiety Inventory, thought patterns measured by the Automatic Thoughts Questionnaire, and thought patterns measured by the Ways of Coping Questionnaire - all measured at 6 weeks and 14 weeks post-baseline. |
| Notes | ClinicalTrials.gov Identifier: NCT04192721 |

**NCT04111887**

| Methods | Randomised controlled trial |
| --- | --- |
| Participants | University students aged 18-28 years with symptoms of depression as indicated by a CES-D depression score of 20 or greater |
| Interventions | 1. Cognitive behavioural prevention intervention 2. Cognitive behavioural prevention intervention with counter-attitudinal elements 3. Educational brochure control |
| Outcomes | Change from baseline Depressive Disorder Diagnostic using the Kiddie Schedule for Affective Disorders and Schizophrenia at 6 weeks, 3 months and 6 months.  Difference in attendance in Change Ahead vs Blues Program groups using group leader notes.  Difference in future onset of Major Depressive Disorder using the Kiddie Schedule for Affective Disorders and Schizophrenia. |
| Notes | ClinicalTrials.gov identifier: NCT04111887 |

**NCT04117789**

| Methods | Randomised controlled trial |
| --- | --- |
| Participants | Adolescents aged 13 to 17 years with a primary diagnosis of mild to moderate major depressive disorder |
| Interventions | 1) Therapist-guided internet-delivered cognitive behavior therapy for depression in adolescents  2) Self-guided internet-delivered cognitive behavior therapy for depression in adolescents  3) Control - Treatment as usual |
| Outcomes | Primary: Depressive symptoms on the Children's Depression Rating Scale - Revised (CDSR-R) at week 10 and 3 months follow-up |
| Notes | ClinicalTrials.gov Identifier: NCT04117789 |

**NCT04192721**

| Methods | Randomised controlled trial |
| --- | --- |
| Participants | Undergraduate nursing students aged 18-25 years with mild to moderate depressive symptoms |
| Interventions | 1. Cognitive behavioural therapy -based group counselling 2. No treatment |
| Outcomes | Beck Depression Inventory  Beck Anxiety Inventory  Automatic Thoughts Questionnaire  Ways of Coping Questionnaire |
| Notes | ClinicalTrials.gov Identifier: NCT04192721 |

**Oregon Research Institute 2018**

| Methods | Randomised Controlled Trial |
| --- | --- |
| Participants | University students aged 18-28 years with depressive symptoms as measured by the CES-D. |
| Interventions | 1) Change Ahead group-based cognitive behavioural prevention programme  2) Blues Program cognitive behavioural group-based prevention programme  3) Placebo - educational brochure about depression |
| Outcomes | Primary: Depressive symptoms on the Kiddie Schedule for Affective Disorders and Schizophrenia (KSADS) at 6 weeks, 3 months, and 6 months, attendance, and future onset of Major Depressive Disorder using the KSADS  Secondary: Activity level, emotions, positive and negative thoughts, assessments using writing samples, depression and anxiety symptoms using the PHQ-9, depression and anxiety levels using the GAD-7, social adjustment in school, work, peer, spare time and family domains, negative life events, health care utilisation, negative automatic thoughts, negative attributional style, and substance use. |
| Notes | ClinicalTrials.gov Identifier: NCT04111887 |

**Salamanca-Sanabria 2018**

| Methods | Randomised controlled trial |
| --- | --- |
| Participants | Adults with mild to moderate depressive symptoms as measured by the Patient Health Questionnaire (PHQ-9) |
| Interventions | 1) Space from Depression online cognitive behavioural therapy intervention  2) Control - waiting list |
| Outcomes | Primary: Depressive symptoms measured by the PHQ-9  Secondary: Anxiety symptoms measured by the Generalized Anxiety Disorder 7  Other: Satisfaction with Treatment Quesionnaire, Helpful Aspects of Therapy Questionnaire |
| Notes | ClinicalTrials.gov Identifier: NCT03062215 |

**Yokomitsu 2020**

| Methods | Randomised controlled trial |
| --- | --- |
| Participants | University students aged 18-30 years with depressive symptoms |
| Interventions | 1) SPARX computerised self-help CBT intervention  2) Wait-list control |
| Outcomes | Primary: Depressive symptoms measured by the Patient Health Questionnaire (PHQ-9) at post-treatment and 1-month follow-up  Secondary: Satisfaction with the programme and satisfaction with life, positive and negative moods, social functioning, rumination, and coping. |
| Notes | Japan Primary Registries Network UMIN000034354; https://tinyurl.com/uu7xd77 |

**Characteristics of ongoing studies [ordered by study ID]**

**Baldofski 2019**

| Study name | E.motion trial |
| --- | --- |
| Methods | Randomised controlled trial |
| Participants | 363 children and adolescents at least 12 years old with subsyndromal symptoms of depression (PHQ-A scores between 5-9). |
| Interventions | 1) Clinicial-guided self-management programme (iFightDepression)  2) Clinicial-guided group chat intervention  3) Control intervention - psycho-educational website on depressive symptoms |
| Outcomes | Primary: Depression symptomatology at the end of the intervention as measured by the PHQ-A.  Secondary: depression symptomatology at all follow-ups, help-seeking attitudes, actual face-to-face help seeking, adherence to and satisfaction with interventions, depression stigma, and utilisation and cost of interventions. |
| Starting date | 21 November 2018 |
| Contact information | Universitätsklinikum Leipzig, Klinik und Poliklinik für Psychiatrie und Psychotherapie  Ms. PD Dr. Christine Rummel-Kluge  Semmelweisstraße 10, Haus 13  04103 Leipzig  Germany |
| Notes | German Register for Clinical Trials (DRKS), DRKS00014668. Registered on 4 May 2018. |

**IRCT20160404027216N7**

| Study name | Effect of Cognitive-Behavior Therapy on the prevention of depression |
| --- | --- |
| Methods | Randomised controlled trial |
| Participants | First semester nursing students |
| Interventions | Intervention: Group cognitive behavioural therapy focused on social skills - six sessions  Control: Free discussion group sessions |
| Outcomes | Depression score on Beck Depression Inventory |
| Starting date | 10 June 2018 |
| Contact information | Fatemeh Vizeshfar, Shiraz University of Medical Sciences, +98 71 3647 4258, vizeshfarf@sums.ac.ir |
| Notes | Author contacted - results not published.  Iranian Registry of Clinical Trials - https://en.irct.ir/trial/33315 |

**IRCT20171231038158N1**

| Study name | A Comparative Study on the Efficacy of a Positive Cognitive-Behavioral therapy and a Cognitive Behavioral Therapy, in depression, subjective well-being, resiliency, self-efficacy and self-esteem for adolescences with depression signs. |
| --- | --- |
| Methods | Randomised controlled trial |
| Participants | Middle school boys aged 13-16 years with mild to moderate depression |
| Interventions | Positive Cognitive-Behavioral therapy and Cognitive Behavioral Therapy |
| Outcomes | Depression, subjective well-being, resiliency, self-efficacy , self-esteem |
| Starting date | 12 December 2017 |
| Contact information | Ali Beygi, Semnan University of Medical Science, +98 23 3263 6889, ali.beygi@semnan.ac.ir |
| Notes | Iranian Registry of Clinical Trials - https://en.irct.ir/trial/28734 |
